# Tracing glacial refugia of *Triturus* newts based on mitochondrial DNA phylogeography and species distribution modeling

**DOI:** 10.1186/1742-9994-10-13

**Published:** 2013-03-20

**Authors:** Ben Wielstra, Jelka Crnobrnja-Isailović, Spartak N Litvinchuk, Bastian T Reijnen, Andrew K Skidmore, Konstantinos Sotiropoulos, Albertus G Toxopeus, Nikolay Tzankov, Tanja Vukov, Jan W Arntzen

**Affiliations:** 1 Naturalis Biodiversity Center, P. O. Box 9517, 2300 RA, Leiden, The Netherlands; 2 University of Twente, Faculty of Geo-Information Science and Earth Observation – ITC, P.O. Box 6, 7500 AA, Enschede, The Netherlands; 3 Faculty of Sciences and Mathematics, University of Niš, Višegradska 33, Niš, 18000, Serbia; 4 Institute for Biological Research, University of Belgrade, Bulevar Despota Stefana 142, Beograd, 11000, Serbia; 5 Institute of Cytology, Russian Academy of Sciences, Tikhoretsky pr. 4, St. Petersburg, 194064, Russia; 6 Department of Biological Applications and Technology, School of Science and Technology, University of Ioannina, GR-45110 University Campus of Ioannina, Ioannina, Greece; 7 National Museum of Natural History, Tsar Osvoboditel blvd. 1, Sofia, 1000, Bulgaria

**Keywords:** Colonization, Contact zone, Historical biogeography, Ice Age, Introgression, Quaternary

## Abstract

**Introduction:**

The major climatic oscillations during the Quaternary Ice Age heavily influenced the distribution of species and left their mark on intraspecific genetic diversity. Past range shifts can be reconstructed with the aid of species distribution modeling and phylogeographical analyses. We test the responses of the different members of the genus *Triturus* (i.e. the marbled and crested newts) as the climate shifted from the previous glacial period (the Last Glacial Maximum, ~21 Ka) to the current interglacial.

**Results:**

We present the results of a dense mitochondrial DNA phylogeography (visualizing genetic diversity within and divergence among populations) and species distribution modeling (using two different climate simulations) for the nine *Triturus* species on composite maps.

**Conclusions:**

The combined use of species distribution modeling and mitochondrial phylogeography provides insight in the glacial contraction and postglacial expansion of *Triturus*. The combined use of the two independent techniques yields a more complete understanding of the historical biogeography of *Triturus* than both approaches would on their own. *Triturus* newts generally conform to the ‘southern richness and northern purity’ paradigm, but we also find more intricate patterns, such as the absence of genetic variation and suitable area at the Last Glacial Maximum (*T. dobrogicus*), an ‘extra-Mediterranean’ refugium in the Carpathian Basin (*T. cristatus*), and areas where species displaced one another postglacially (e.g. *T. macedonicus* and western *T. karelinii*). We provide a biogeographical scenario for *Triturus*, showing the positions of glacial refugia, the regions that were postglacially colonized and the areas where species displaced one another as they shifted their ranges.

## Introduction

The Quaternary Ice Age (~2.59 Ma-present) is associated with large climatic oscillations [1-4]. Long, cold and dry glacial cycles are alternated by relatively short, warm and wet interglacials. The transition between the two takes place in a geological blink of an eye [5,6]. The climatic oscillations have a major impact on species distribution patterns [1]. Generally, impacts of glacial cycles are more extreme further away from the equator (and at higher elevations): areas at a higher latitude (and elevation) become inhospitable, whereas climate at a lower latitude (and elevation) remains habitable [1,7]. Populations at higher latitude may cope with climate change *in situ*, through adaptation or phenotypic plasticity, or alternatively they may track suitable habitat [6,8]. However, a more likely outcome is that such populations go extinct [8-10]. At lower latitudes, populations can endure glacial periods relatively unimpaired, in so called glacial refugia [2,10,11]. During subsequent interglacials, species can reclaim their former distribution, by rapidly recolonizing the large tracts of still uninhabited, but freshly habitable land from glacial refugia [1].

The fluctuating climate during the Quaternary has left its mark on patterns of genetic diversity [1-3,5,12]. Populations persisting in glacial refugia have a relatively long and stable demographical history compared to those in areas claimed postglacially. As a result, populations in glacial refugia are characterized by high levels of genetic diversity, whereas populations established after the most recent glacial cycle typically show little genetic variation. This is the concept – devised from a northern hemisphere perspective – of ‘southern richness and northern purity’ [1]. Furthermore, species displacement after postglacial secondary contact regularly coincides with introgression of genetic material (especially of cytoplasmic DNA) [12,13]. By uncovering the spatial structuring of genetic lineages between glacial refugia and along recolonization routes, and by detecting mismatches between genetic markers and species boundaries, phylogeographical surveys provide insights in past distribution rearrangement [9].

Independent from genetics, species distribution modeling can be applied to answer historical biogeographical questions [14]. Species distribution modeling involves the approximation of the ecological requirements of a species, based on the range of environmental conditions experienced at known localities [15]. The constructed model can then be extrapolated on current climate layers, to determine the species’ potential distribution. Similarly, the model can be projected on climatological reconstructions of the past. Niches evolve over time, questioning the validity of predicting past distributions based on present day models. However, considering the relatively short time period spanning the shift from the last glacial cycle to the present day, niche conservatism is a realistic assumption [16,17]. Comparison of present and past potential distribution provides information on range shifts.

**Figure 1:**
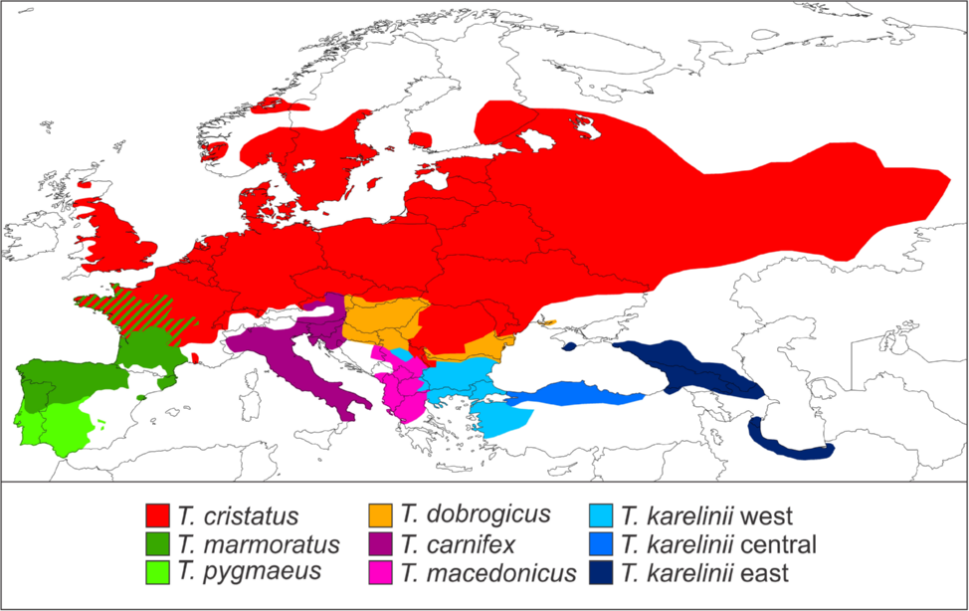
**The distribution of the genus *Triturus*.** Species mostly meet at parapatric contact zones, but note the area of sympatry of *T. marmoratus* and *T. cristatus* in western and central France. This map is adapted from [25].

The distribution of amphibians is tightly linked to environmental conditions and thus has to follow suit in the face of rapid climate change [18-21]. The marbled and crested newts (genus *Triturus*) are a group of nine closely related species [22,23] (Table 1). *Triturus* newts are distributed across most of Europe and adjacent Asia (Figure 1). They are found in regions generally regarded as important glacial refugia, such as the Iberian, Italian and Balkan Peninsulas, Anatolia, Caucasia and the southern Caspian basis [3,9,10]. On the other hand, *Triturus* newts occupy large tracts of land which would have been uninhabitable during glacial periods, particularly temperate Eurasia [24]. Thus, within this single model system, we expect to observe varying responses to glaciation.

**Table 1:** **Systematics of the genus *Triturus***

**Species**	**Authority**
**Marbled newts**	
*Triturus marmoratus*	(Latreille, 1800)
*Triturus pygmaeus*	(Wolterstorff, 1905)
**Crested newts**	
*Triturus cristatus*	(Laurenti, 1768)
*Triturus carnifex*	(Laurenti, 1768)
*Triturus macedonicus*	(Karaman, 1922)
*Triturus dobrogicus*	(Kiritzescu, 1903)
*Triturus karelinii* *	(Strauch, 1870)
eastern species	
central species	
western species	

*The taxon traditionally referred to as *T. karelinii* actually comprises three distinct species for which the taxonomy still has to be worked out fully [22,23]. For now these three species are referred to as the western, central and eastern *T. karelinii* species.

We first conduct a dense phylogeographical survey: employing mitochondrial DNA, we determine geographical genetic structuring (i.e. diversity within and divergence among populations) for each of the different *Triturus* species. We also delineate areas where mitochondrial DNA has been asymmetrically introgressed. Subsequently, we approximate the distributions of the different *Triturus* species at the Last Glacial Maximum (~21Ka) using species distribution modeling. The outcome of the two independent approaches is visualized on composite maps, which show the nine different species side by side. Our hypothesis is that those areas predicted suitable at the Last Glacial Maximum based on species distribution models are also the ones showing the highest genetic diversity. Vice versa, we expect areas suggested to have been unsuitable at the time are genetically depleted. We summarize the signatures of past distribution dynamics as inferred from mitochondrial DNA phylogeography and species distribution modeling on a map.

## Methods

### Genetic approach

Generally the identification of *Triturus* newts is straightforward based on geography (Figure 1). For individuals occurring near the contact zone more care should be taken. The *Triturus* individuals included were in the first place identified based on morphology [26,27] and for a subset nuclear genetic data confirming their identity [23] (Arntzen et al., submitted; Wielstra et al., unpublished data).

**Figure 2:**
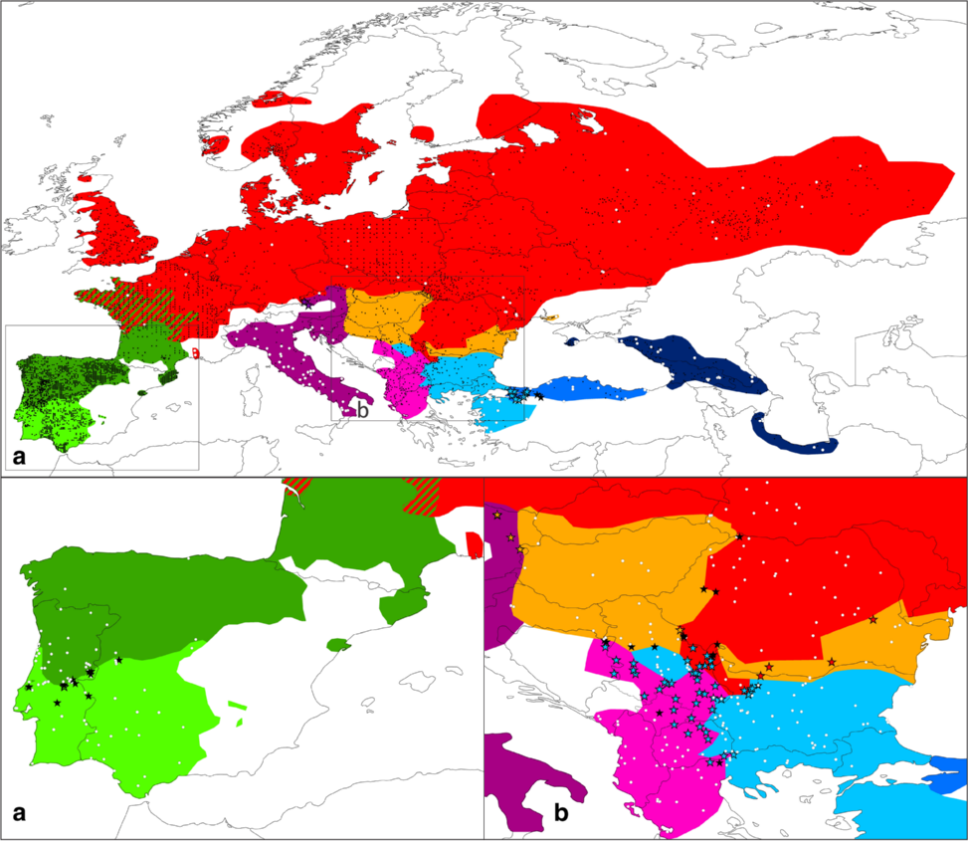
**Maps showing the *Triturus *localities used for the mitochondrial DNA phylogeography and species distribution modeling.** The inset shows part of the localities used in the genetic analyses (white circles) and the additional ones used for species distribution modeling (black circles). Two cut-outs (A and B) show more details on introgressed mitochondrial DNA: populations containing foreign mitochondrial DNA are labeled with a colored star (with the color denoting the ‘donor’ species) and populations containing two mitochondrial DNA types (of which one mostly, but not exclusively, is of the original species) as a black star; populations containing original mitochondrial DNA are again labeled with white circles. The colors used correspond to Figure 1. Details on localities can be found in Dataset S1 and S2.

We included genetic data (658 bp of subunit 4 of the NADH dehydrogenase gene complex; ND4) for 2470 *Triturus* newts, representing 493 populations (Figure 2 and Additional file 1). A large proportion of these individuals (n = 1795) was taken from previous studies (published [25,28-32] or submitted [Arntzen et al.]; see Additional file 1 for details). The remainder (n = 675) was newly sequenced for the current paper, using the primers in Table 2 and following the protocol outlined in [33]. Sequences were manually aligned and identical ones merged into haplotypes using MacClade 4.08 [34]. Our purpose was 1) to test for mitochondrial DNA introgression between species, and 2) to infer the spatial genetic variation within each species.

**Table 2:** Primers used for amplification and sequencing of the ND4 mitochondrial DNA fragment

**Species or groups of species with their forward and reverse primers**	**Source**
***T. marmoratus***	
MARF1: CACCTGTGATTACCTAAAGCTCATGTAGAAGC	This study
ND4R2: CCCTGAAATAAGAGAGGGTTTAA	[33]
***T. pygmaeus***	
PYGF1: CACCTCTGATTGCCTAAAGCCCACGTAGAGGC	This study
ND4R2: CCCTGAAATAAGAGAGGGTTTAA	[33]
***T. karelinii *group of crested newts**	
KARF4: AGCGCCTGTCGCCGGGTCAATA	[33]
KARR1: AACTCTTCTTGGTGCGTAG	[33]
**Other crested newts**	
KARF4: AGCGCCTGTCGCCGGGTCAATA	[33]
DOBR2: GTGTTTCATAACTCTTCTTGGT	[33]

To detect which individuals retain their ‘original’ mitochondrial DNA and which possess introgressed mitochondrial DNA (derived from one of the other species), we first assigned mitochondrial DNA haplotypes to species by conducting a Neighbor Joining analysis in MEGA 5.05 [35]. *Calotriton asper* was used as outgroup (sequence taken from [28]; GenBank accession number: GU982378).

To infer interspecific geographical structuring, we first excluded introgressed mitochondrial DNA (as it does not properly reflect the evolutionary history of either ‘host’ or ‘donor’). Subsequently, we determined – for each species – 1) the genetic diversity within populations and 2) the genetic distance among populations. The mean number of pairwise differences among haplotypes (π), as determined with Arlequin 3.5 [36], was used as a measure of genetic diversity within populations. To determine the genetic distance among populations we used Alleles in Space 1.0 [37]. Alleles in Space connects the populations in a network, based on Delaunay triangulation. Subsequently, the program produces values of average genetic distance among the populations that are connected by the network (the proportion of mismatched nucleotide sites; *Z*_*i*_). These values are positioned at the midpoints of the connections in the network. For the measure of genetic diversity within populations we only included populations for which more than one sequence was available (π will always be zero for populations with only one sequence).

We interpolated the values for π and *Z*_*i*_ across geographical space using inverse distance weighting in the spatial analyst extension of ArcGIS (http://www.esri.com) [38]. The output for each species was cropped according to its distribution range (Figure 1). For both π and *Z*_*i*_, we compiled a single composite map from the nine crops (i.e. one for each *Triturus* species). We used a color scheme running from blue to red to reflect low to high values of π and *Z*_*i*_. Using a single scale for all *Triturus* species facilitates comparison among them, but runs the risk that variation in genetically poor species is overshadowed by that of genetically rich species. Hence, we additionally apply a species specific scale to better express intraspecific structuring. This way we managed to visualize 1) regions of relatively high and low intraspecific genetic diversity and 2) the relative genetic divergence among populations within species.

### Species distribution modeling approach

We composed a database of 4532 *Triturus* localities (incorporating the 493 populations included in the genetic analysis; see Figure 2 and Additional file 2). This database is based on [39] and updated in the light of the taxonomic developments [23] (see Table 1). For most species the majority of localities concern accurate locations, but in particularly for the wide ranging *T. cristatus* we mostly only had the name of the nearest village of a locality or digitized atlas data to our disposal. Species distribution models are still expected to perform relatively accurately if spatial error in localities is introduced and the method we use (Maxent, see below) is robust against such errors [40]. We used the bioclimatic variables available at a 2.5 arcminute resolution (c. 5 × 5 km) from the WorldClim database version 1.4 [41] to calibrate our species distribution models. In order to prevent model overfitting, which would negatively influence transferability [16,42], we minimized multicolinearity among data layers by selecting a subset that showed a Pearson’s correlation of r < 0.7. Furthermore, focusing on climate layers that are deemed biologically meaningful based on life history knowledge of the model system yields the most appropriate species distribution models [43]. Variation in the availability of standing water during the breeding season appears to have driven the ecological radiation among *Triturus* newts [25]. Taking these two points into consideration, we included a set of layers that encompasses seasonal variation in evaporation and precipitation, in casu bio10 = mean temperature of warmest quarter, bio11 = mean temperature of coldest quarter, bio15 = precipitation seasonality, bio16 = precipitation of wettest quarter, and bio17 = precipitation of driest quarter. Bioclimatic variables are also available from the WorldClim database for the Last Glacial Maximum (~21 Ka). These data are derived from the Paleoclimate Modelling Intercomparison Project phase 2 [44]; [http://pmip2.lsce.ipsl.fr/] and based on two climate simulations: the Model for Interdisciplinary Research on Climate version 3.2 (MIROC) and the Community Climate System Model version 3 (CCSM) [45].

Species distribution models were created with Maxent 3.3.3 k [46]. We restricted the feature type to hinge features as this produces smoother model fits, so forcing models to be focused on key trends rather than potential idiosyncrasy in the data. This approach facilitates extrapolation to a different time (or place) [47]. The environmental range covered by the pseudo-absence data, used to discriminate presence data from background, should neither be too narrow nor too broad [48-50]. Too narrow a range results in complex models that do not generalize well, whereas too broad a range results in too simple models that focus on coarse-scale and neglect fine-scale variation. A practical solution is to focus on area that is potentially accessible to the species of interest if it were not for abiotic factors, i.e. an area where spread would not be hampered by major physical barriers. However, competition could lead to further exclusion of the target species, even though the area would be suited in the absence of such biotic interactions [51]. Taking these considerations into account, we restricted the area from which pseudo-absence was drawn to the distribution of the entire genus *Triturus*. This area was broadly defined as a 200 km buffer zone [48] around known *Triturus* localities (Additional file 2).

The species distribution models were tested for statistical significance against a null model derived from random localities [52]. For each tested species distribution model, we created a null distribution of 99 AUC values. These AUC values were derived from species distribution models, based on as many random localities as used for the tested species distribution model. The AUC value of the tested species distribution model was treated as a 100^th^ value and deemed statistically significant if it ranked higher than the 95^th^ value (i.e. above the 95% confidence interval). Random point data were created with ENMTools 1.3 [53]; [http://enmtools.blogspot.com/]. The null model approach prevents interpreting model quality based on an arbitrary AUC threshold and precludes the requirement to set aside part of the localities for model testing [52].

The species distribution models were projected on the current and Last Glacial Maximum climate layers. Composite maps were created for each set of climate layers (in the same way as explained for the genetic approach). Maxent provides predicted probability values between zero and one and we used a color scheme running from blue to red to reflect these values.

## Results

**Figure 3:**
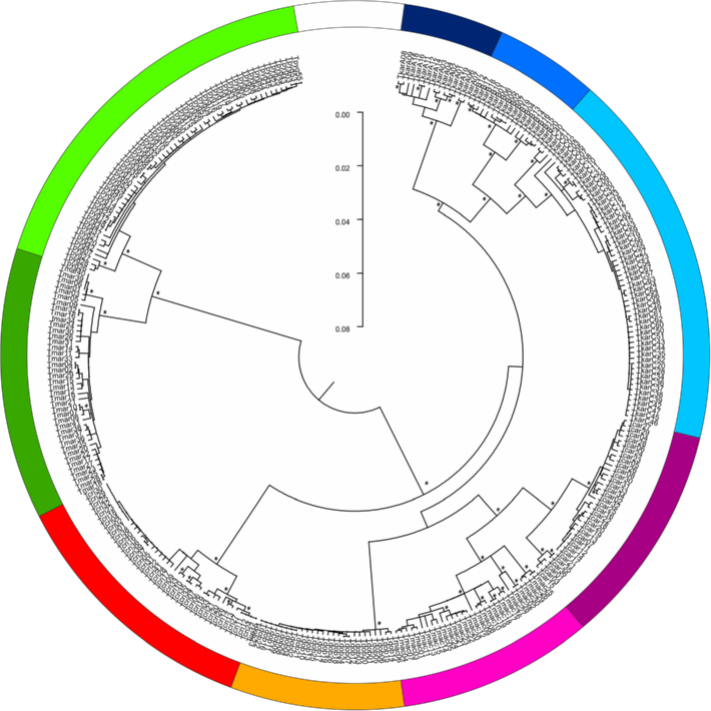
**A Neighbor Joining phylogeny for the *Triturus *ND4 haplotypes.** The *Triturus* haplotypes cluster into nine monophyletic mitochondrial DNA lineages, corresponding to species and colored as in Figure 1. Significantly supported branches (≥ 80%, based on a thousand bootstrap replicates) are denoted with an asterisk. The *Calotriton asper* outgroup used to root the phylogeny is not shown.

The 2470 *Triturus* sequenced newts comprise 315 haplotypes (see Additional file 1 for details and Additional file 3 for GenBank accession numbers). The Neighbor Joining phylogeny shows that haplotypes group in nine reciprocally monophyletic lineages, corresponding to species (Figure 3). A large number of individuals (n = 408; c. 16.5%) that belong to one species, possess mitochondrial DNA characteristic of another (Figure 2 and Additional file 1). This mitochondrial DNA introgression is mostly restricted to near the contact zones; only *T. pygmaeus*, *T. macedonicus*, *T. cristatus* and central *T. karelinii* have extended ranges in which foreign mitochondrial DNA is present (derived from *T. marmoratus* for the first one and from western *T. karelinii* for the other three).

**Figure 4:**
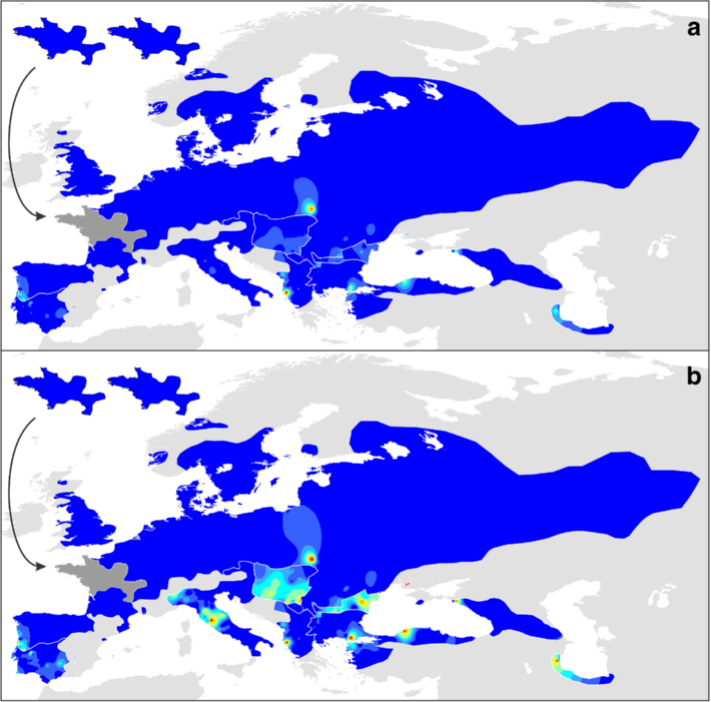
**The geographical distribution of genetic variation for the different *Triturus *species.** These are composite maps for all nine *Triturus* species. For each species, the genetic variation within each population (π) was determined and subsequently interpolated across its distribution range. In Figure 4a we use a single scale for all *Triturus* species (allowing direct comparison among species) whereas in Figure 4b we use a species specific scale (better expressing genetic structure in genetically relatively poor species). Warmer colors refer to a higher genetic diversity. The insets show the situation for *T. cristatus* (left) and *T. marmoratus* (right) in their area of sympatry (shown in dark gray on the main maps).

**Figure 5:**
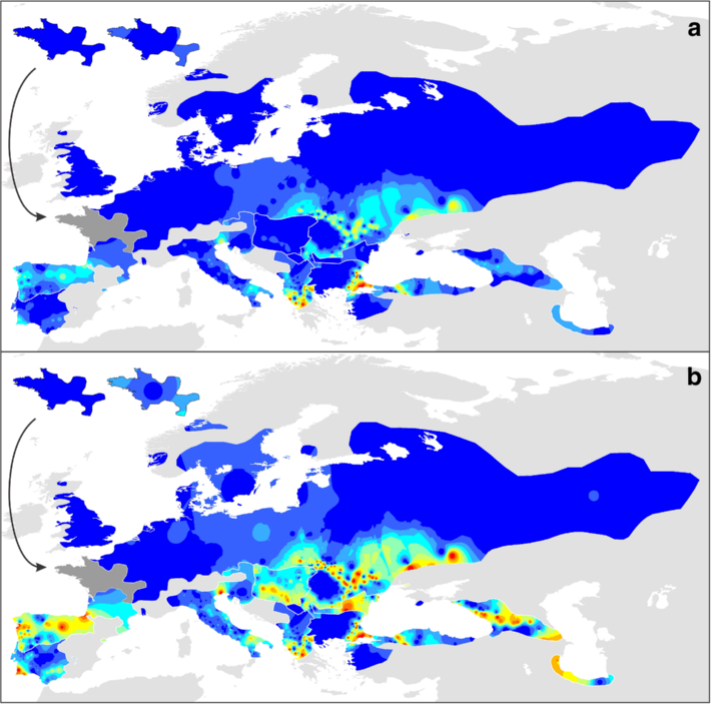
**The genetic (dis)similarity among populations within the different *Triturus *species.** These are composite maps for all nine *Triturus* species. For each species, the genetic divergence among populations (*Z*_*i*_) was determined and subsequently interpolated across its distribution range. In Figure 5a we use a single scale for all *Triturus* species (allowing direct comparison among species) whereas in Figure 5b we use a species specific scale (better expressing genetic structure in genetically relatively poor species). Warmer colors refer to a higher genetic divergence. The insets show the situation for *T. cristatus* (left) and *T. marmoratus* (right) in their area of sympatry (shown in dark gray on the main maps).

The number of populations included to determine genetic diversity within (π) and genetic divergence among (*Z*_*i*_) populations was 44 and 48 out of 52 populations for *T. carnifex*, 60 and 88 out of 104 for *T. cristatus*, 29 and 40 out of 44 for *T. dobrogicus*, 55 and 79 out of 79 for western *T. karelinii*, 13 and 16 out of 24 for central *T. karelinii*, 27 and 31 out of 31 for eastern *T. karelinii*, 32 and 37 out of 72 for *T. macedonicus*, 34 and 36 out of 36 for *T. marmoratus* and 49 and 50 out of 51 for *T. pygmaeus* (populations containing asymmetrically introgressed mitochondrial DNA were excluded completely and populations represented by only a single individual were additionally excluded in the calculation of π). Measures of genetic diversity within and genetic divergence among populations can be found in Additional file 1 and 4. Composite maps visualizing genetic structuring in each species are depicted in Figures 4 and 5. Because Alleles in Space places the values for genetic divergence among populations at the midpoint of the network connecting the populations, information falling outside the current range is lost in Figure 5 (e.g. in the case of two allopatric populations). Uncropped maps for each species, showing a more comprehensive picture per species but at the cost of conciseness, can be found in Additional file 5. *Triturus* species generally show considerable spatial variation in their genetic composition (reflected by ‘warm’ and ‘cold’ areas in Figures 4 and 5).

**Figure 6:**
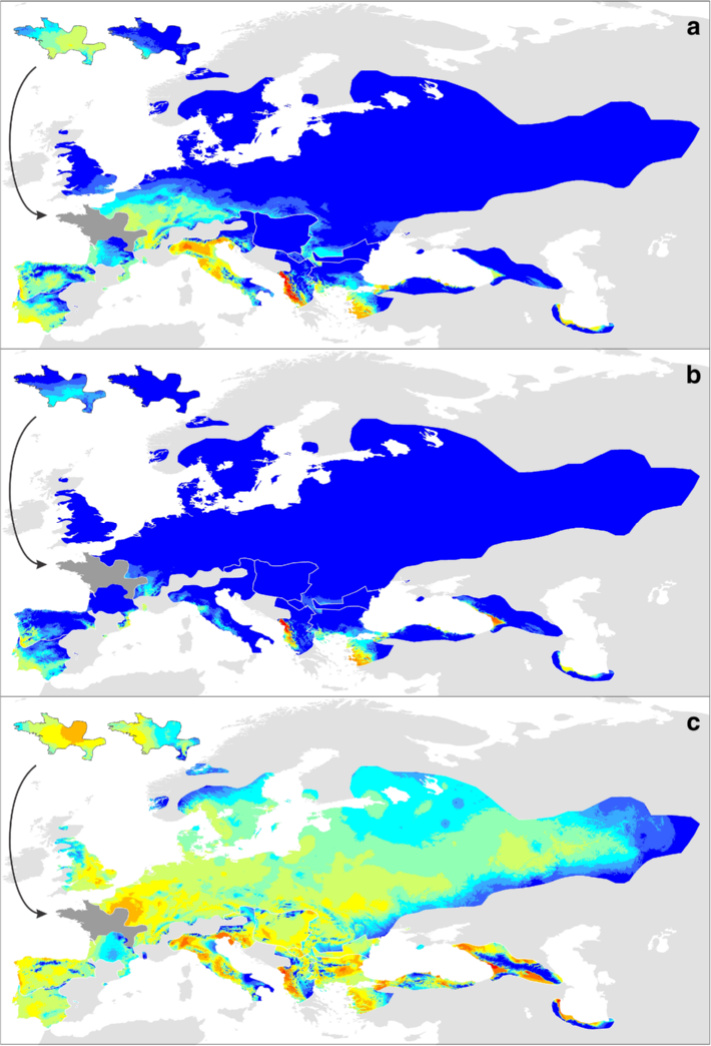
**The predicted suitability of each *Triturus *species’ range at the Last Glacial Maximum (MIROC and CCSM model) and at the present.** This is a composite map for all nine *Triturus* species. For each species, its species distribution model was projected on MIROC (**a**) and CCSM (**b**) Last Glacial Maximum climate layers and on current climate layers (**c**), cut according to its current distribution range. Warmer colors refer to a higher predicted suitability. The insets show the situation for *T. cristatus* (left) and *T. marmoratus* (right) in their area of sympatry (shown in dark gray on the main map).

Species distribution models perform significantly better than random (Additional file 6). Composite maps depicting the predicted suitability of each species’ distribution range at the Last Glacial Maximum, based on the two different climate simulations (MIROC and CCSM), are provided in Figure 6. As species were not necessarily bound to their current ranges through time, projections for each species on a wider area (roughly the distribution of the entire genus *Triturus*) are provided in Additional file 7. The ranges of all *Triturus* species are predicted to have been restricted at the Last Glacial Maximum. A composite map showing species distribution models projected on present day climate layers is provided in Figure 6. Predicted suitability of the distribution range of each species under current conditions shows a considerable overlap with the sketched outlines of their ranges (Figure 1).

## Discussion

Before we discuss our results, we first consider where care should be taken when interpreting genetic or species distribution modeling results in isolation and explain how interpretation of the results of the two independent techniques together at least partially negates these problems. Then we identify the probable glacial refugia and postglacially colonized area for each *Triturus* species. Finally, we summarize our scenario regarding the biogeographical response of *Triturus* newts to the climate shift from the previous glacial period to the current interglacial on a map.

### Considerations and combination of the genetic and species distribution modeling results

A high value for genetic diversity within populations (Figure 4) can result from two distinct processes: postglacial secondary contact from distinct source populations or long term presence *in situ* throughout glacial-interglacial cycles (potentially enforced by a low connectivity between populations due to e.g. xeric environment) [9]. To separate these two processes, phylogenetic relationships among haplotypes provide some insight. For example, the obvious ‘hotspot’ found in *T. cristatus* (SE Poland) likely reflects the comingling of haplotypes belonging to the two distinct clades present in this species (Figure 3; Additional file 1). On the other hand, the hotspot found in eastern *T. karelinii* (SE Azerbaijan) likely represents standing genetic variation, given the lack of phylogeographic structure among the haplotypes involved. Species distribution modeling can further help to distinguish both processes by establishing whether an area became suitable only recently or whether it has continuously been so on the long term.

Maps showing genetic divergence among populations (Figure 5) reflect a different aspect of geographical genetic structuring. On the one hand, areas with a high genetic overturn among populations (e.g. the southern part of the range of *T. macedonicus*) show many hotspots. Such areas would be expected to have been habitable on the long term. On the other hand, genetically homogenous areas, due to high levels of gene flow and/or demographic expansion stand out as cold areas (e.g. both of these processes likely determine the absence of genetic structuring among *T. dobrogicus* populations). These two processes are more difficult to disentangle: whereas demographic expansion would particularly be expected in areas that became suitable postglacially, gene flow could reduce genetic structuring both inside and outside of glacial refugia. In some cases geographical barriers to gene flow stand out, such as the Greater Caucasus mountain range separating eastern *T. karelinii* on its northern and southern side. Such barriers reflect climatically unsuitable regions and can be identified as such in the species distribution models.

The reliability of genetic diversity values for populations depends on sample size, and some populations are relatively poorly sampled in our study. The genetic turnover between populations provides additional insight that counters the adverse effects of low sample sizes. If populations within an area are genetically diverse, but sample sizes per population are low, genetic diversity per population might be underestimated due to chance effects in sampling. However, these effects are random per population. Therefore, the more populations that are being compared within that area, the less likely it is that actual genetic diversity remains hidden by chance. For this same reason determining the genetic turnover between populations is less susceptible to sample size to begin with: low sample size per population adds up to a big sample sizes within a region. So, in short, if multiple populations within a region are found to be genetically similar, even though samples sizes per population are low, together they provide a good indication that genetic diversity in the region really is low. We thus recommend exploring genetic diversity within populations and genetic divergence among populations together to improve the estimate of spatial genetic variation, especially when sample sizes are low.

We present the results of a single (mitochondrial) genetic marker. Although we consider our results indicative (and use the species distribution modeling results as an independent check), it should be taken into account that a single marker may not accurately reflect species history [54]. In fact, we explicitly exploit the mismatch between species history and mitochondrial DNA in the case of asymmetrically introgressed mitochondrial DNA. Asymmetrically introgressed mitochondrial DNA may suggest that species displaced one another (e.g. [29,55]). For *Triturus*, the similarity of the introgressed mitochondrial DNA to that present in the ‘donor’ species suggests a recent (postglacial) transference across the species boundary. Given the climatic oscillations during the Quaternary Ice Age, the *Triturus* newt species are presumed to have been in periodic contact through time. However, we did not identify ancient mitochondrial DNA introgression events (as in e.g. [56-58]). A potential explanation is that introgressed mitochondrial DNA was limited to areas of postglacial expansion and never penetrated those areas that repeatedly acted as glacial refugia. In effect, any mitochondrial DNA that became introgressed during an interglacial would be erased by the next glacial period. Species distribution modeling allows us to test this hypothesis.

A disadvantage of species distribution models is that they cannot distinguish between potential and realized distribution: areas that had suitable conditions at the Last Glacial Maximum may not have actually been inhabited. Genetic information can narrow down the position of glacial refugia (e.g. both France and the Carpathian Basin are suggested to have had suitable conditions during the Last Glacial Maximum for *T. cristatus* but only the latter shows the high genetic variation expected to be present in a former glacial refugium). Another issue is that species distribution models are based on the climatic conditions currently experienced by a species, but the actual conditions under which they could occur might be broader (e.g. no suitable conditions were predicted to have been present at the Last Glacial Maximum for *T. dobrogicus*, but yet it did survive to the present day).

### Glacial refugia and area postglacially colonized for each *Triturus* species

The two marbled newt species illustrate the different effect of glaciations on species with a relatively southern and relatively northern distribution. The northern *T. marmoratus* is predicted to have withdrawn its range at the Last Glacial Maximum more extensively than the southern *T. pygmaeus* (especially according to the CCSM climate simulation; Figure 6). In *T. marmoratus* genetic structuring is highest in the south of its range, but for *T. pygmaeus* it is not (Figures 4 and 5). The presence of *T. marmoratus* mitochondrial DNA in the northern part of the range of *T. pygmaeus* suggests that in this area the former was replaced by the latter [31,59,60]. This is confirmed by the distribution models projected on Last Glacial Maximum climate data outside of the current species ranges (Additional file 7): at the time, area predicted suitable for *T. marmoratus* (but not for *T. pygmaeus*) was present in the northern part of the area currently occupied by *T. pygmaeus*. Striking is that both *T. marmoratus* and *T. pygmaeus* mitochondrial DNA occurs throughout this area (Figure 2). Although a species will often simply show foreign mitochondrial DNA where it replaced another [13] see below for examples in *Triturus*, exceptions similar to the *T. marmoratus*-*T. pygmaeus* case are known (e.g. [61]).

The species distribution models based on the two climate simulations agree that most of the range of *T. cristatus* was unsuitable at the Last Glacial Maximum (Figure 6). Suitable area was suggested to be present at the Last Glacial Maximum in both the Carpathian Basin and in France. The genetic data suggest that only the Carpathian Basin acted as a glacial refugium at the Last Glacial Maximum as genetic diversity here is high (Figures 3, 4, 5). This patterns corresponds to the geographical distribution of genetic variation based on nuclear DNA markers found in previous studies (MHC genes, allozymes and microsatellites [62,63]). This also suggests that the remainder of *T. cristatus*’ current range (including France) was colonized post-glacially, as the species is genetically depleted outside of the Carpathian Basin. Although *T. cristatus* shows asymmetrically introgressed western *T. karelinii* mitochondrial DNA south of the Danube river, relatively distinct *T. cristatus* haplotypes also occur here (Figures 2 and 3, Additional file 1), suggesting a complex pattern of both range expansion at the cost of western *T. karelinii* but also long term presence south of the Danube.

For *T. carnifex* there is a discrepancy between the two climate simulations, with MIROC showing a more extensive predicted distribution at the Last Glacial Maximum than CCSM. Particularly CCSM suggests a more considerable range reduction and no suitable area in the current northern Balkan range (Figure 6). The genetic data better correspond to the modeled distribution based on MIROC, as it shows distinct genetic clusters distributed on both sides of the Adriatic Sea and high genetic diversity in Italy (Figures 3, 4, 5), supporting long term presence in the Balkans and a stable population in Italy (see [32] for more detail). Both climate simulations agree that *T. carnifex* colonized the part of its range east and north of the Alps postglacially. The *T. carnifex* mitochondrial DNA introgressed into *T. cristatus* where the two species meet suggests that Balkan *T. carnifex* were involved in this range expansion.

Compared to the other *Triturus* species, intraspecific genetic diversity (i.e. phylogenetic depth) is low in *T. dobrogicus* (Figure 3). *Triturus dobrogicus* shows a ‘starburst’ phylogeny – a large number of similar haplotypes – suggesting a demographic expansion after a bottleneck. At the Last Glacial Maximum, the range of *T. dobrogicus* is predicted to have been unsuitable (Figure 6). This is in line with a population bottleneck. However, the species distribution models do not make it possible to pinpoint a potential refugium: the entire currently occupied range was predicted unsuitable at the Last Glacial Maximum. Suitable area was also not available outside the current range (Additional file 7). Evidently, *T. dobrogicus* must have had a refugium somewhere and given its specialization on river floodplains (e.g. [64]) this was likely positioned within its current range. Furthermore, compared to the other *Triturus* species *T. dobrogicus* does not show reduced genetic diversity of nuclear DNA (allozymes [65]). This suggests that perhaps the actual ecological niche of *T. dobrogicus* is broader than is currently realized (and used to construct the species distribution models). Another option would be that the niche of *T. dobrogicus* has evolved in a brief time interval to adapt to the environmental shift [66]. We consider this less likely as *T. dobrogicus* must have survived multiple glacial-interglacial cycles *in situ*.

The western part of the current range of *T. macedonicus* is predicted to have been suitable during the Last Glacial Maximum (Figure 6). This does not fully correspond to mitochondrial DNA data, which shows structuring in the southern part of the range, including the southeast, but not in the northwest (Figures 4 and 5). Genetic variation in *T. macedonicus* is the highest of all *Triturus* newts (Figure 3). In a large part of its range, *T. macedonicus* possesses western *T. karelinii* mitochondrial DNA (Figure 2). This can be explained by *T. macedonicus* displacing western *T. karelinii* there after the Last Glacial Maximum as the area only became unsuitable for *T. macedonicus* after the Last Glacial Maximum (Figure 6; see [29] for a detailed analysis).

In western *T. karelinii*, a basal split separates a clade occurring in European Turkey and extreme southeastern Bulgaria (Figure 3). The species distribution modeling approach suggests suitable area was present here during the Last Glacial Maximum (Figure 6). The genetic distinction of this clade is expressed by the ‘barrier’ in Figure 5 surrounding the populations in which it is represented. The other clade shows extensive structuring in Asiatic Turkey, suggesting the region acted as a refugium. Suitability at the Last Glacial Maximum is confirmed by the species distribution modeling approach. A single sub-clade derived from (i.e. nested within) Asiatic Turkey has recently colonized the Balkan part of the range; it shows a starburst phylogeny in line with demographic growth during a range expansion [12]. The species distribution modeling approach agrees that the area only became hospitable after the Last Glacial Maximum.

**Figure 7:**
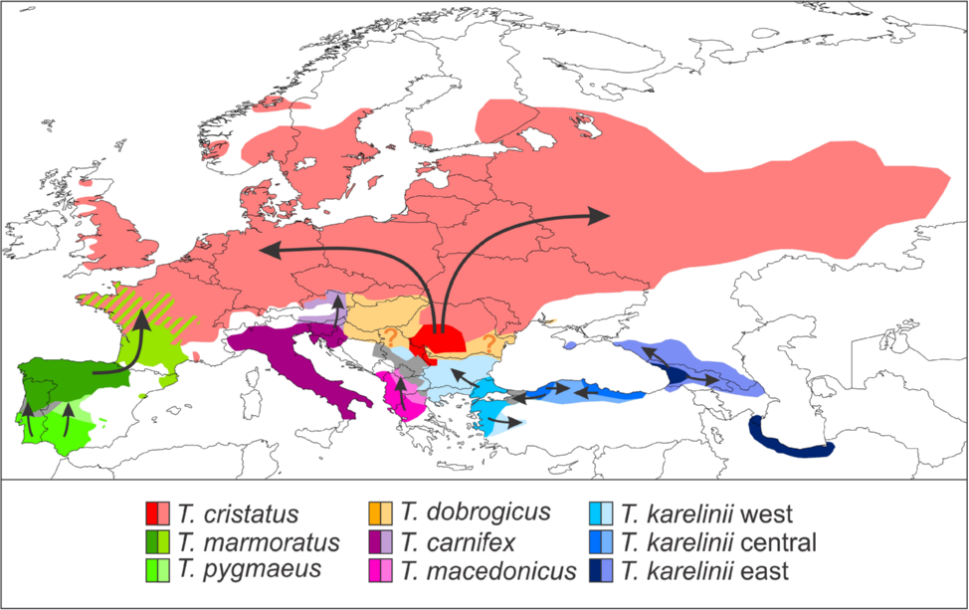
**Biogeographical scenario for *Triturus *in response to the last glacial to current interglacial climate shift.** Shown are the inferred approximate positions of glacial refugia (dark shades) and postglacially colonized area (light shades; arrows reflect approximate colonization routes). Areas where species displaced one another (containing asymmetrically introgressed mitochondrial DNA) are shown in grey. For *T. dobrogicus*, neither the genetic nor species distribution modeling approach manages to recover a refugium (reflected by question marks). See text for further details.

Central *T. karelinii* shows a fragmented distribution at the Last Glacial Maximum (Figures 6 and 7). This is reflected by an east west divide (the ‘barrier’ in Figure 5) between two distinct clades separated by a basal split (Figure 3), which are currently in secondary contact (the ‘warm’ spot in Figure 4). Central *T. karelinii* contains western *T. karelinii* mitochondrial DNA in northwestern Asiatic Turkey (Figure 2), suggesting postglacial species displacement (see [23] for a detailed analysis).

For eastern *T. karelinii*, the southern Caspian basin and western Georgia (Colchis) are suggested to have harbored suitable area at the Last Glacial Maximum according to species distribution models (Figure 6). Genetic diversity is particularly high along the southern Caspian Sea shore (Figures 3, 4, 5). Relatively recently a single nested clade colonized the Caucasus and Crimea from the Caspian part of the range [28]. However, this clade is still distinct (reflected by a relatively deep coalescence; see Figure 3), suggesting that colonization happened during a previous interglacial and thus that the clade persisted here in a glacial refugia during one or several glacial cycles.

### Historical biogeographical scenario

We provide a historical biogeographical scenario for the genus *Triturus* in Figure 7, summarizing glacial refugia, areas of postglacial expansion and regions where species displaced one another. The genetic and species distribution modeling results provide insight into the distribution dynamics of *Triturus* in response to climate change during the Quaternary Ice Age. *Triturus* conforms to the general pattern of the Iberian, Italian and Balkan Peninsulas functioning as glacial refugia [1,67]. Furthermore, several regions that are increasingly appreciated as glacial refugia are also identified as such for *Triturus*: Anatolia, the southern Caspian Basin and the Caucasus region (e.g. [10,68]). The novel idea of extra-Mediterranean refugia, positioned more to the north [69], also applies to *Triturus*: the glacial refugium of *T. cristatus* was situated in the Carpathian Basin.

Similarly, areas typically identified as having been postglacially colonized from glacial refugia are predicted as such for *Triturus*: *T. marmoratus*, *T. carnifex* and (to a lesser extent) ‘eastern *T. karelinii*’ extended their ranges into temperate Europe from their southerly positioned refugia. However, postglacial expansion is best exemplified by *T. cristatus*. The contemporary range of *T. cristatus* outside its glacial refugium in the Carpathian Basin encompasses a huge (c. 4.75 million km^2^) postglacially acquired area, stretching all the way from western Europe to Scandinavia and central Russia. This gives an indication of the speed with which postglacial colonization can be accomplished.

## Conclusions

The combination of phylogeography and species distribution modeling aids locating of glacial refugia [70]. The two approaches to visualize intraspecific geographical genetic structuring together illustrate which areas are genetically rich and thus suspected to reflect long term inhabited area and which areas are genetically poor and thus probably only recently became habitable (Figures 4 and 5). Additionally, asymmetrically introgressed mitochondrial DNA suggests where species displaced one another upon post-glacial secondary contact (Figure 2). Independently, the two reconstructions of potential distribution at the Last Glacial Maximum (based on the MIROC and CCSM climate simulations) provide an indication of which areas were habitable at the time and which were unsuitable for *Triturus* (Figure 6). We provide a qualitative comparison of the results of the two independent techniques (summarized in Figure 7). We anticipate that future developments in the synthesis of phylogeography and species distribution modeling will make it possible to integrate the two approaches into a single analysis even further.

## Competing interests

The authors declare that they have no competing interests.

## Authors’ contributions

BW, AKS, AGT and JWA designed the study. BW, JCI, SNL, BR, KS, NT, TV and JWA collected data. BW analyzed the data. BW drafted the manuscript. All authors read and approved the final manuscript.

## Supplementary Material

Additional file 1 **Genetic sampling.** Details on the *Triturus* populations included in the genetic analysis. Click here for file

Additional file 2 **Locality data.** Details on the *Triturus* populations used for species distribution modeling. Click here for file

Additional file 3 **Haplotype list.** A list of ND4 haplotypes with their GenBank accession numbers. Click here for file

Additional file 4 **Genetic distances.** Values of average genetic distance among populations (Z_i_) within each *Triturus* species. Click here for file

Additional file 5 **Full genetic similarity maps The genetic (dis)similarity among populations within the different *Triturus* species, not cut according to the current species ranges.** Click here for file

Additional file 6 **Model performance.** The AUC values for the species distribution models of each *Triturus* species, tested against null models. Click here for file

Additional file 7 **Full species distribution models.** The species distribution model of each *Triturus* species projected for Last Glacial Maximum and current climate conditions, not cut according to the current species ranges. Click here for file
