# Supplementary material for: Tracing glacial refugia of Triturus newts based on mitochondrial DNA phylogeography and species distribution modeling
Source: Front Zool. 2013 Mar 20;10:13. doi: 10.1186/1742-9994-10-13 (PMC3608019; doi:10.1186/1742-9994-10-13)

Additional file 5. Full genetic similarity maps. The genetic (dis)similarity among populations within the different *Triturus* species, not cut according to the current species ranges. For each species, the genetic divergence among populations (Z_i) was determined and subsequently interpolated across its distribution range. We use both a single scale for all *Triturus* species (allowing direct comparison among species) and aspecies specific scale (better expressing genetic structure in genetically relatively poor species). Warmer colors refer to a higher genetic divergence.

Single scale *T. carnifex*

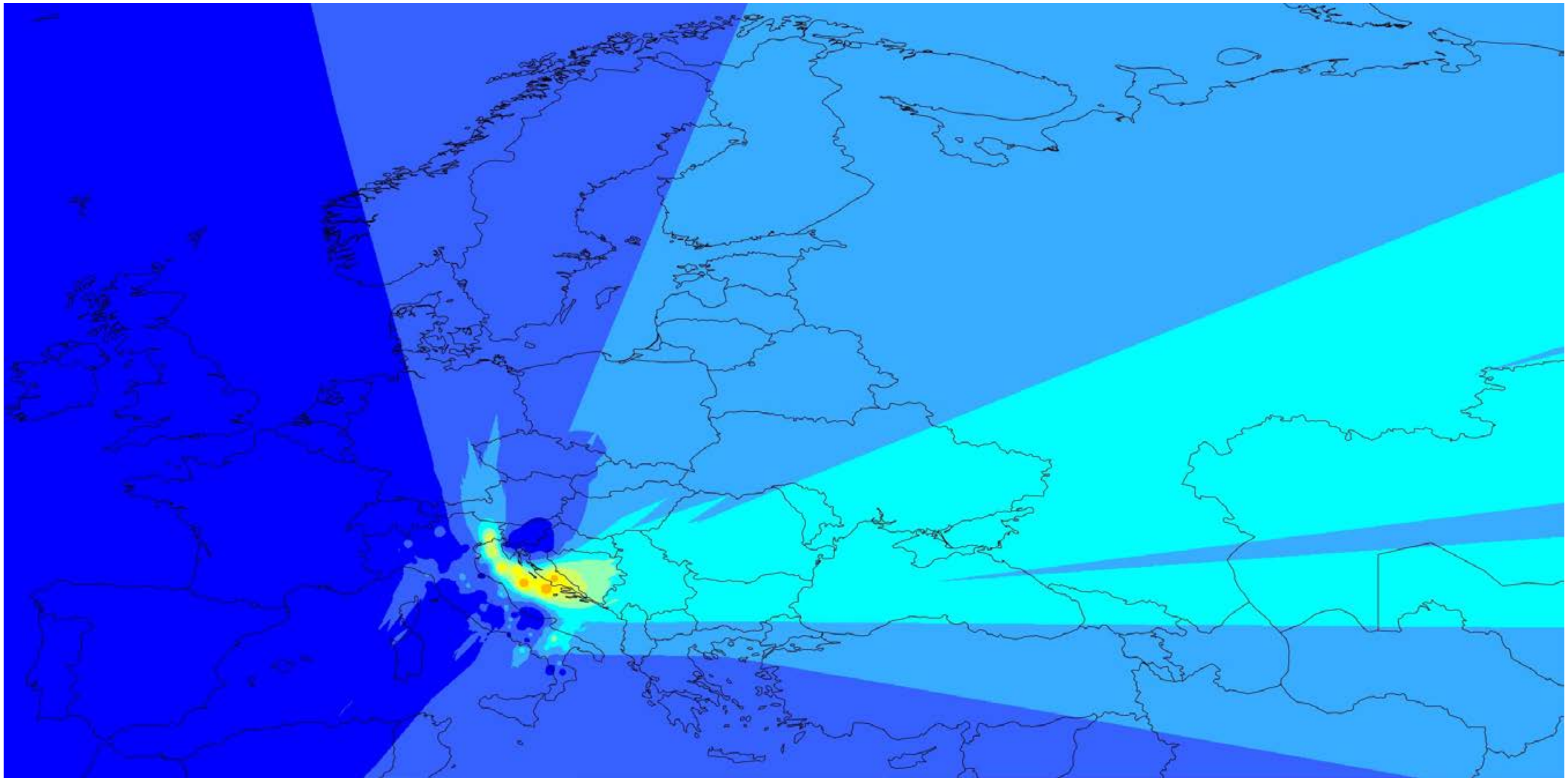

Single scale *T. cristatus*

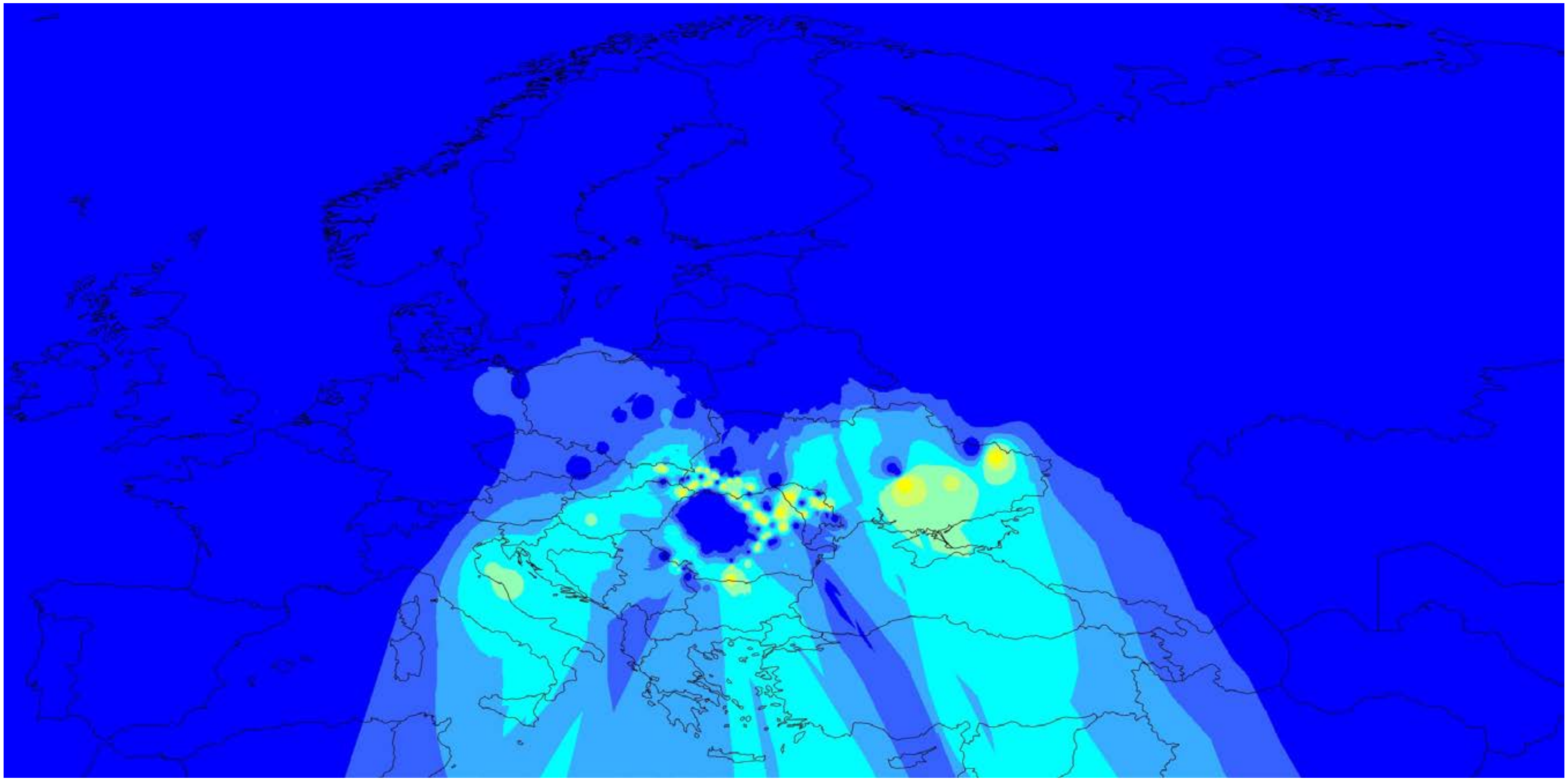

Single scale *T. dobrogicus*

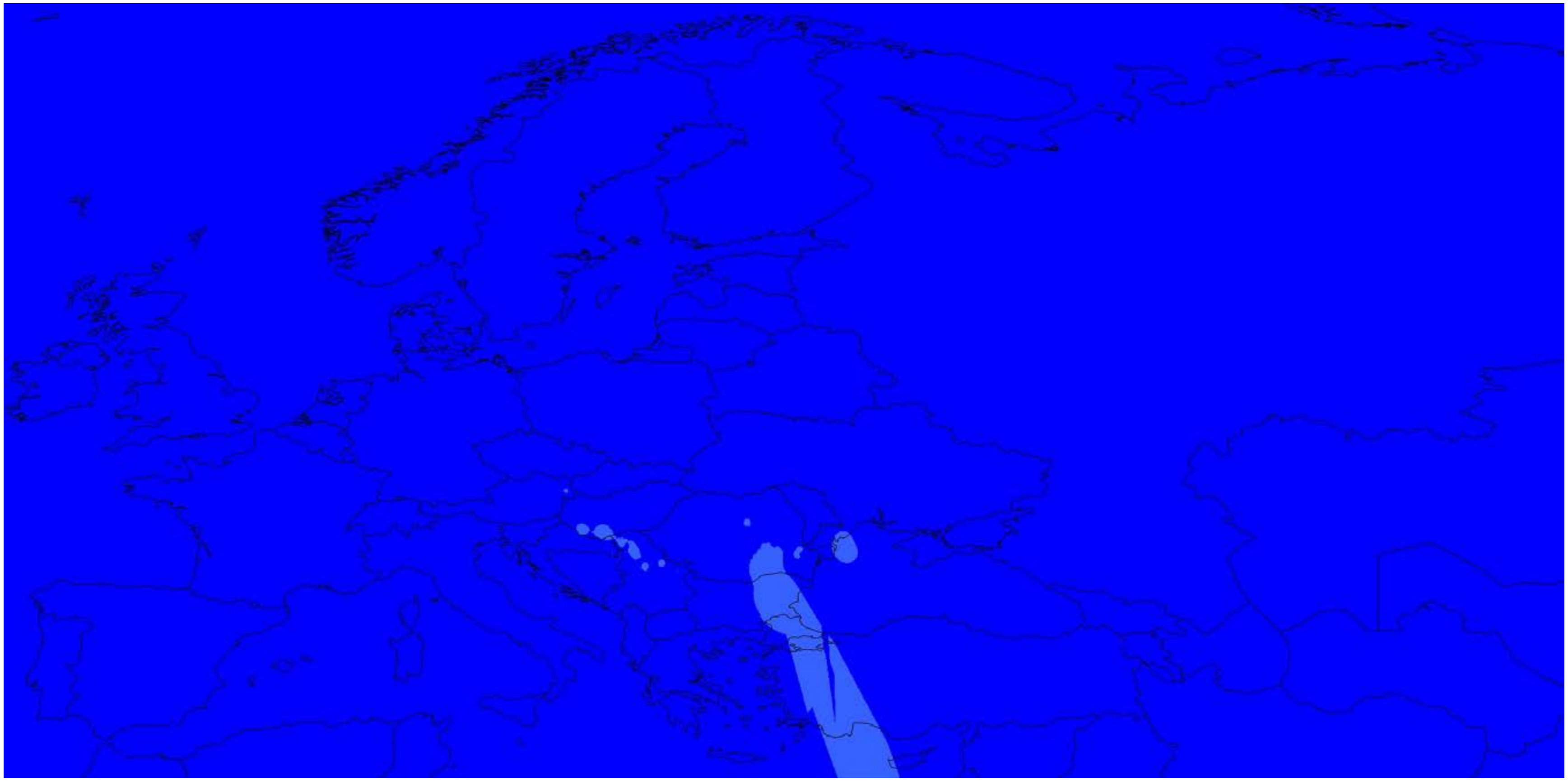

Single scale *T. karelinii* western species

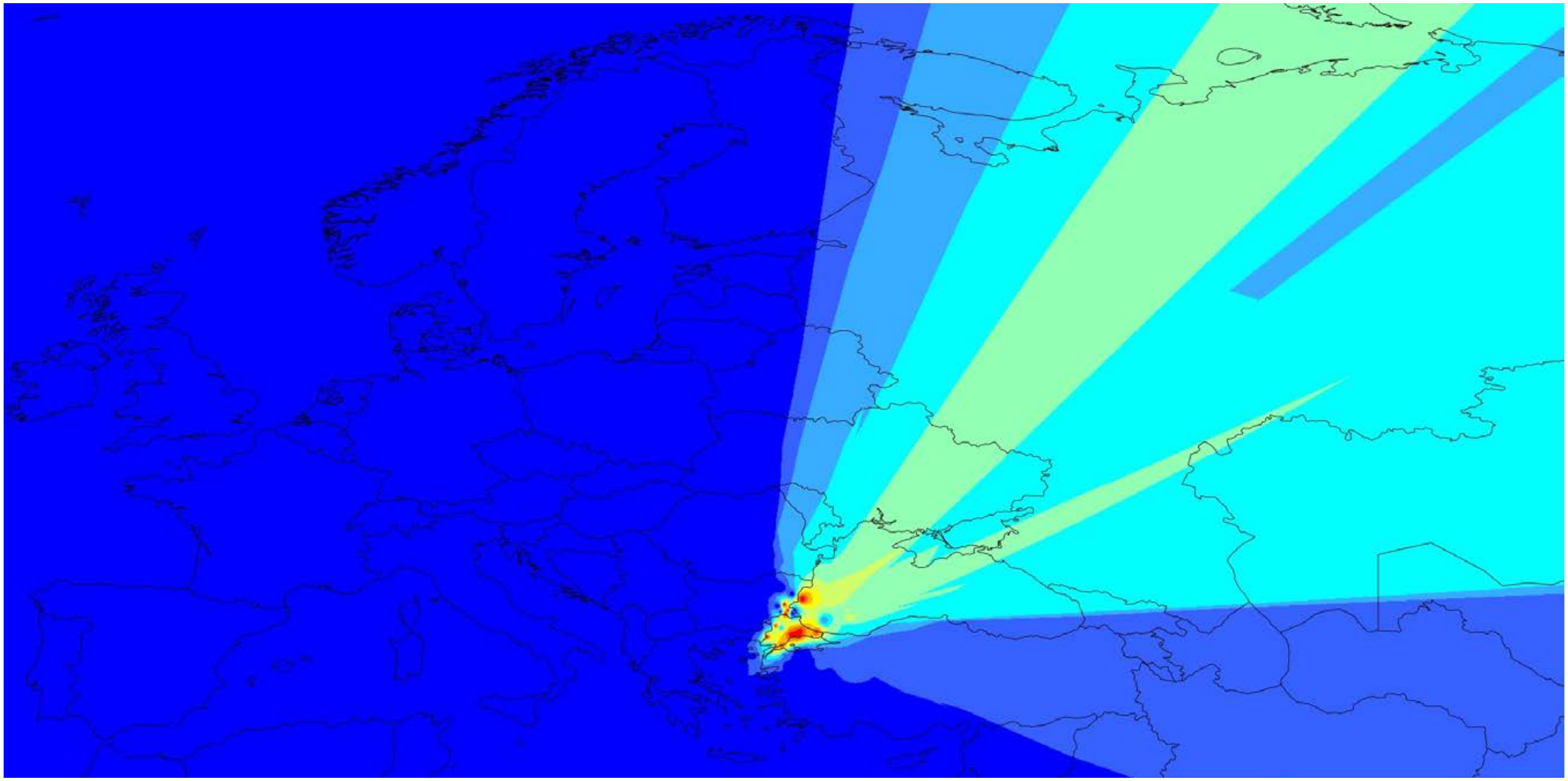

Single scale *T. karelinii* central species

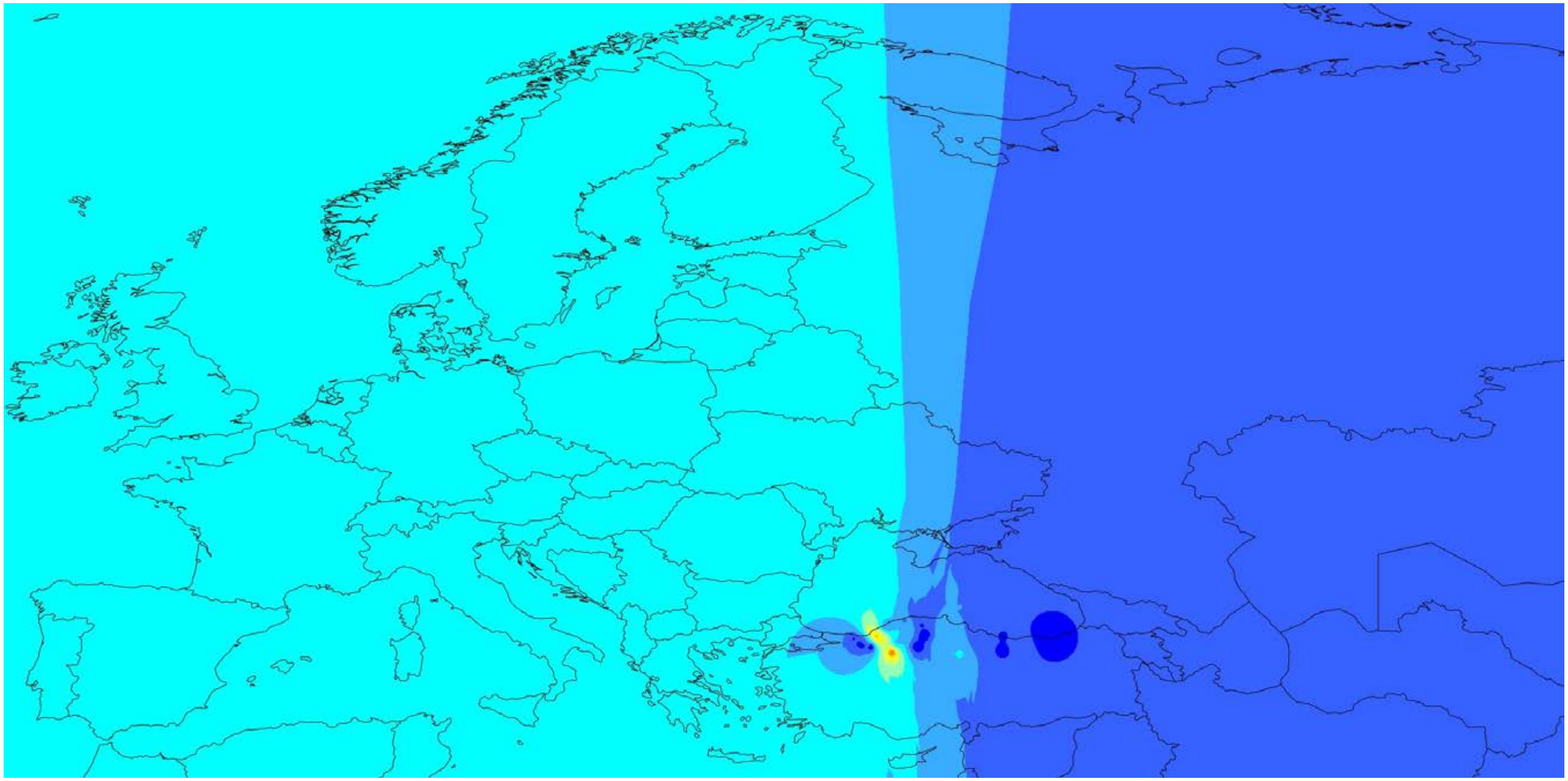

Single scale *T. karelinii* eastern species

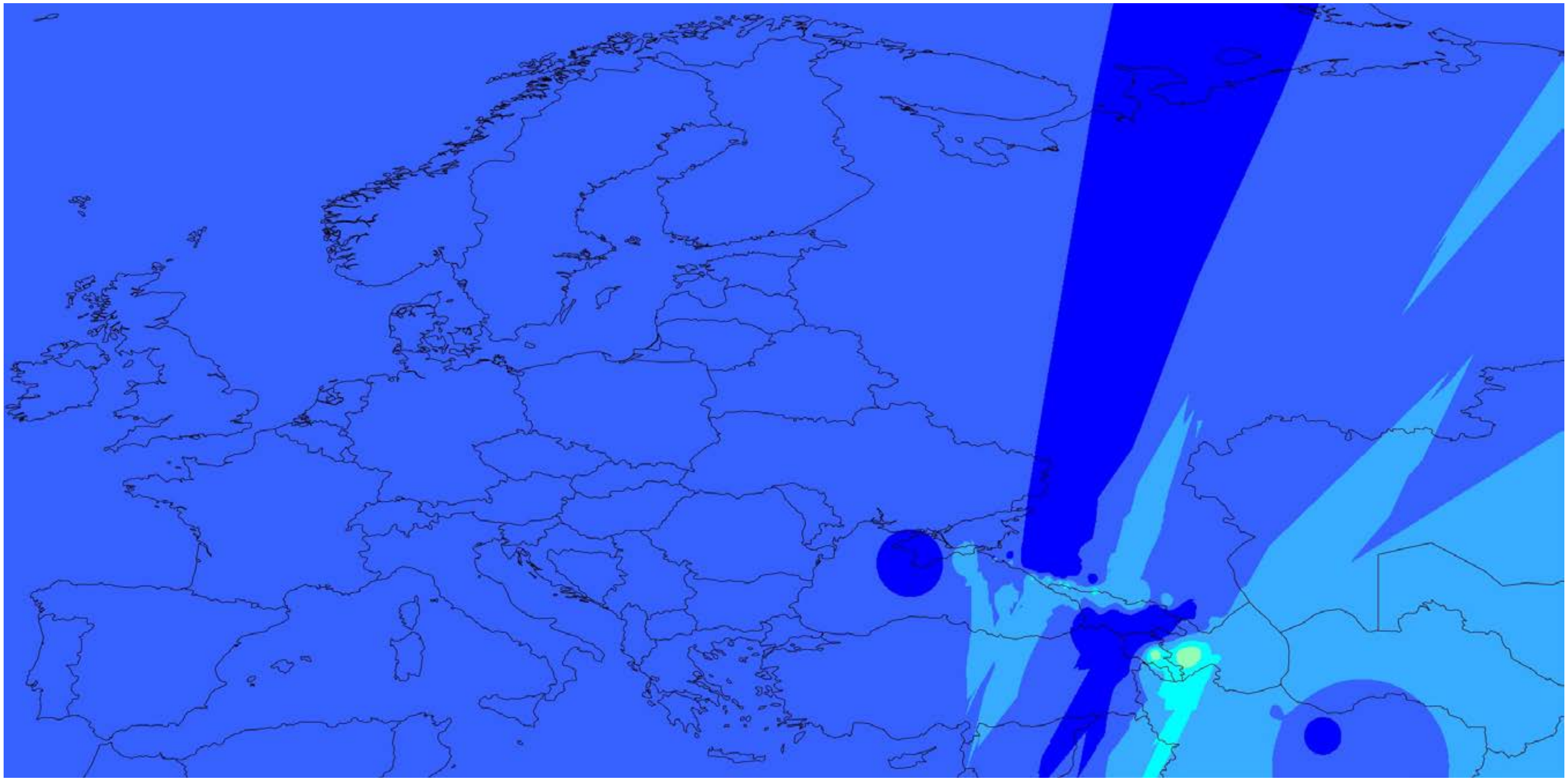

Single scale *T. macedonicus*

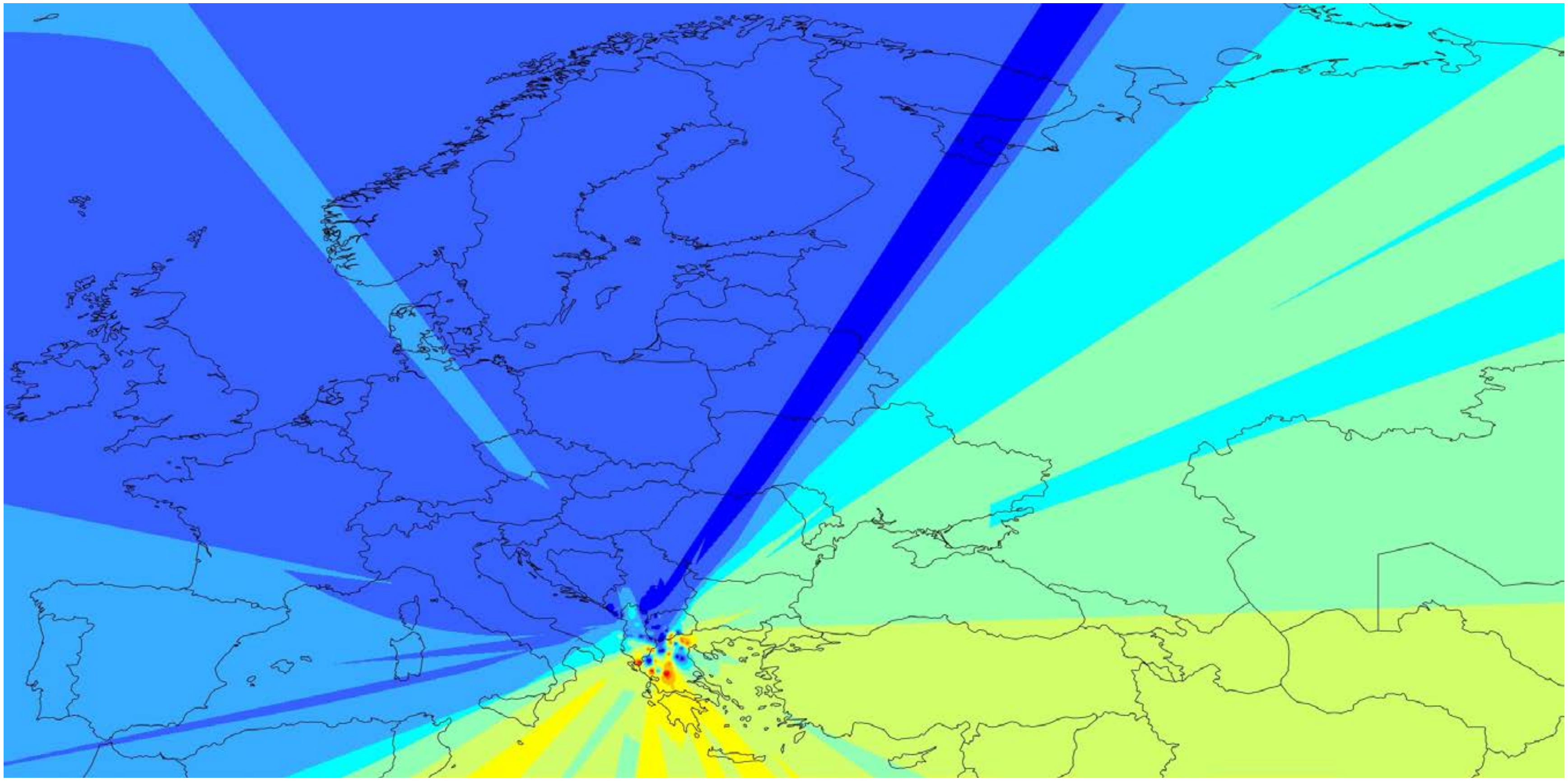

Single scale *T. marmoratus*

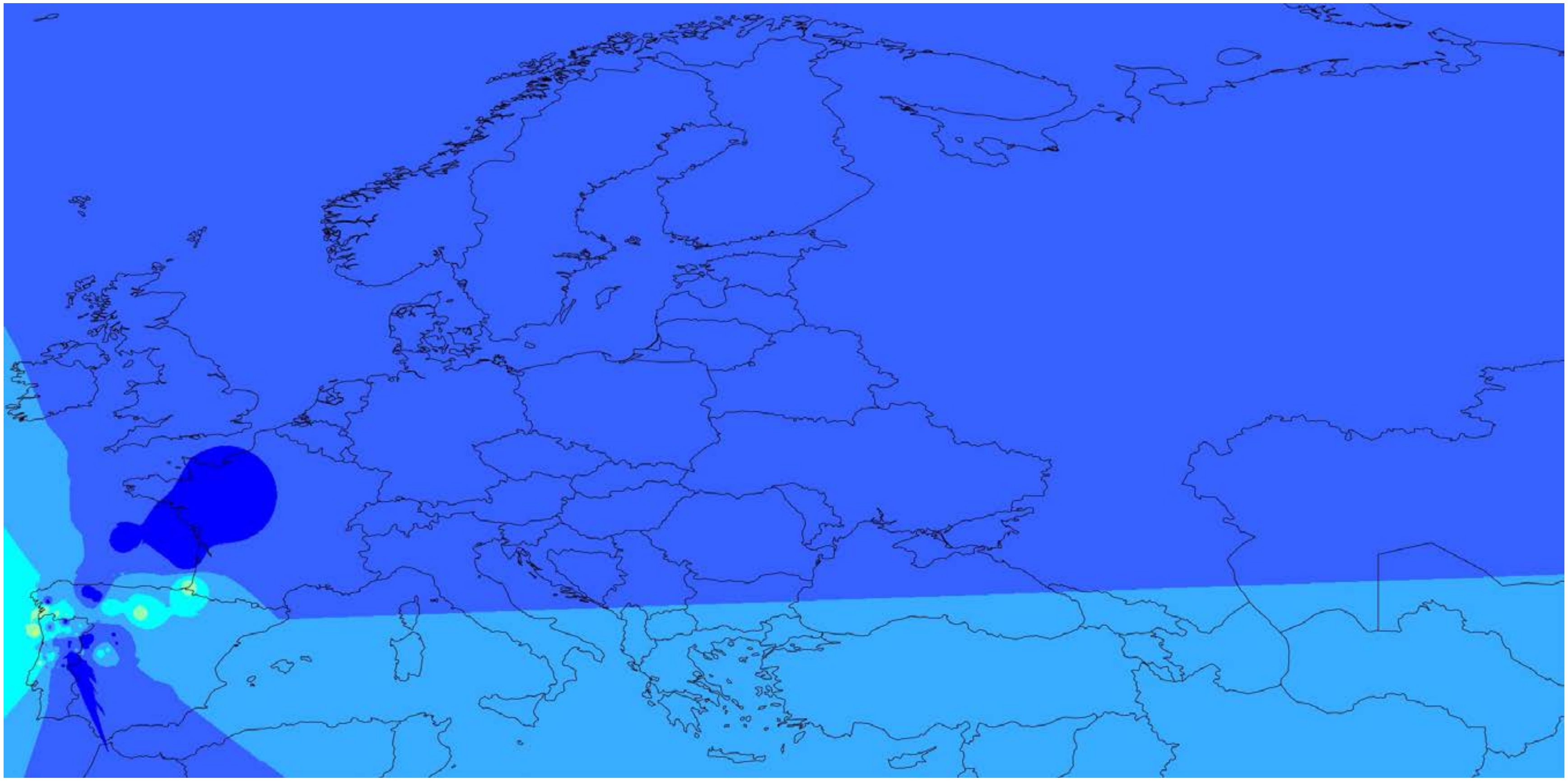

Single scale *T. pygmaeus*

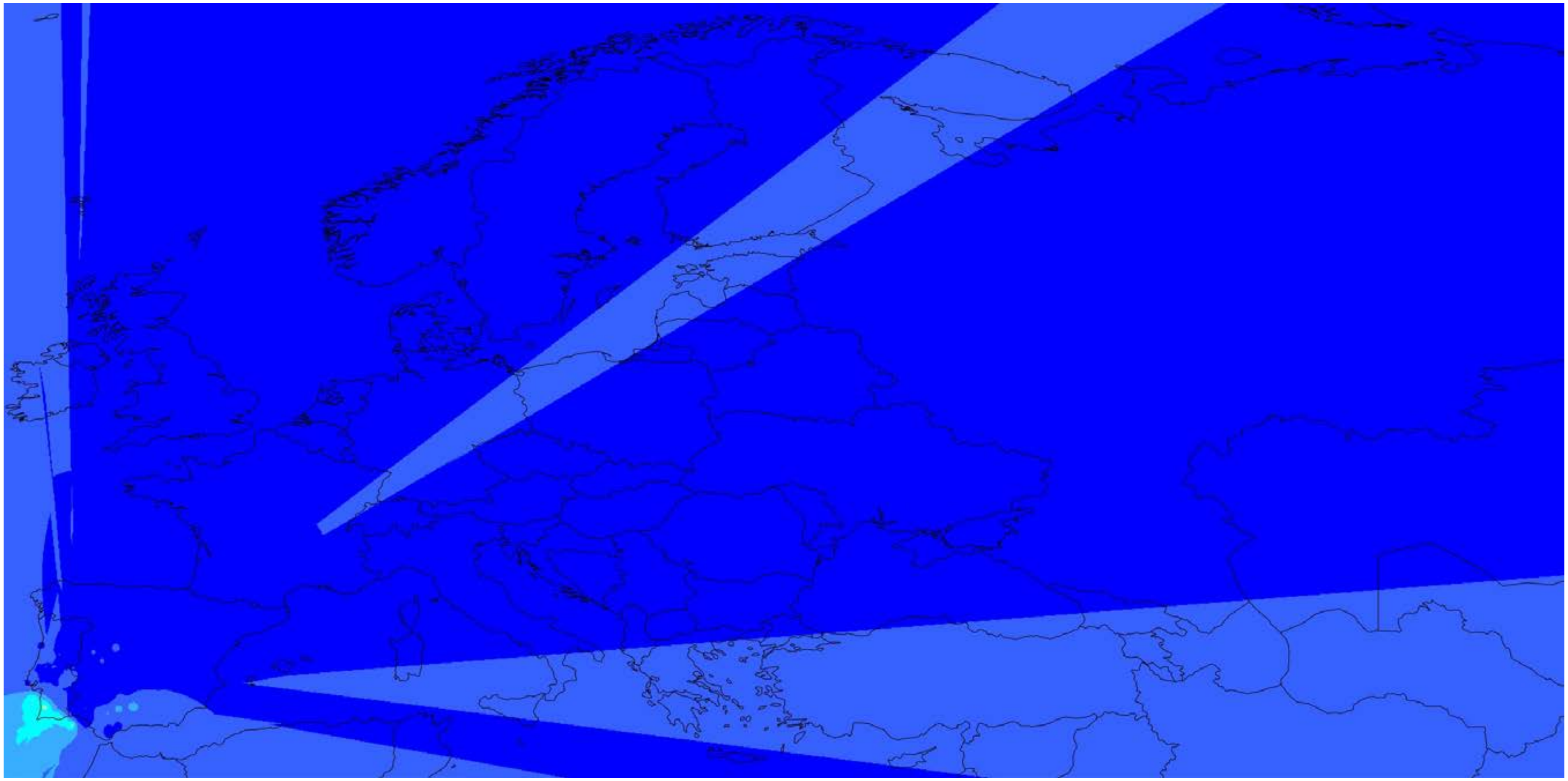

Species specific scale *T. carnifex*

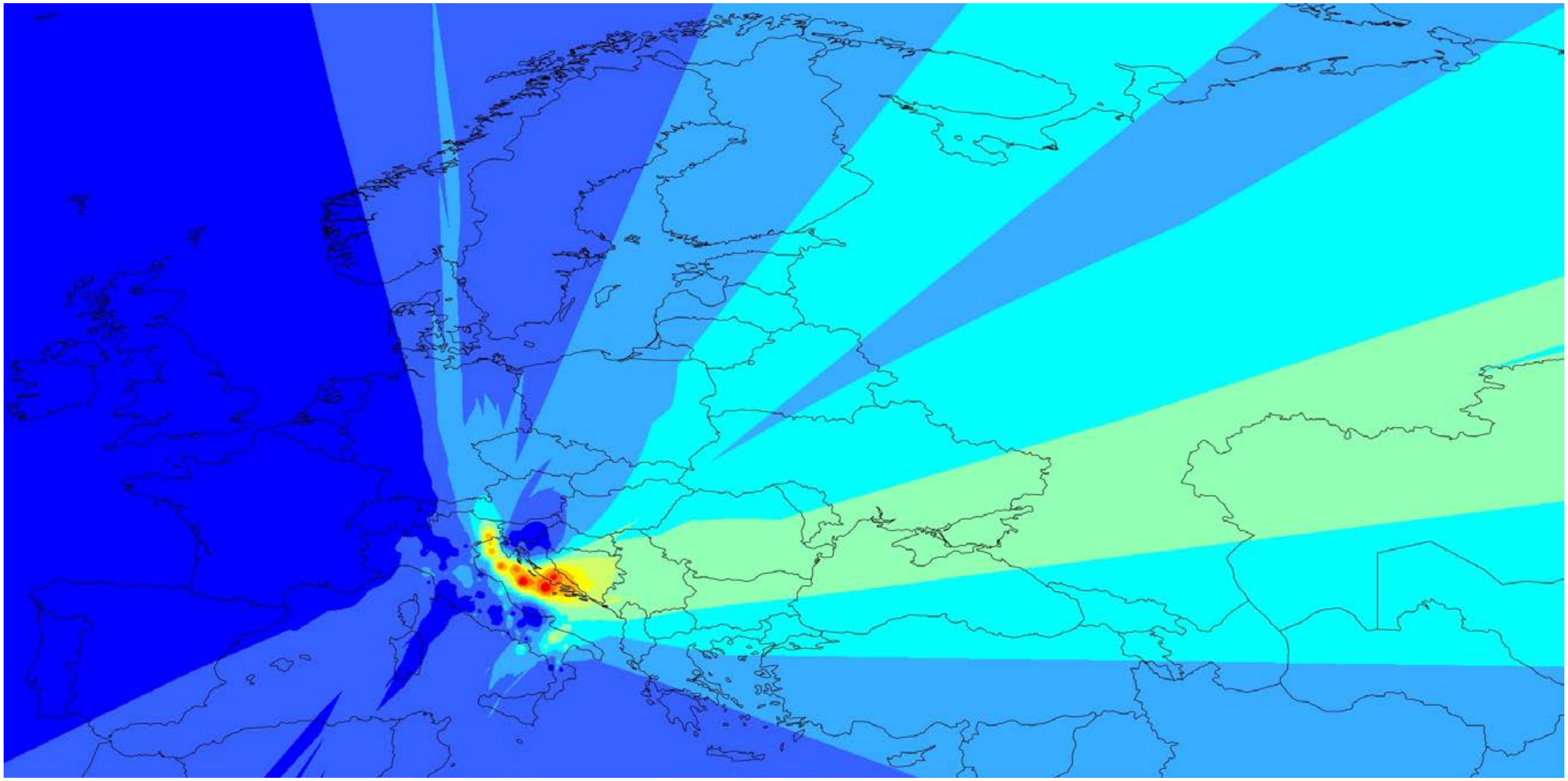

Species specific scale *T. cristatus*

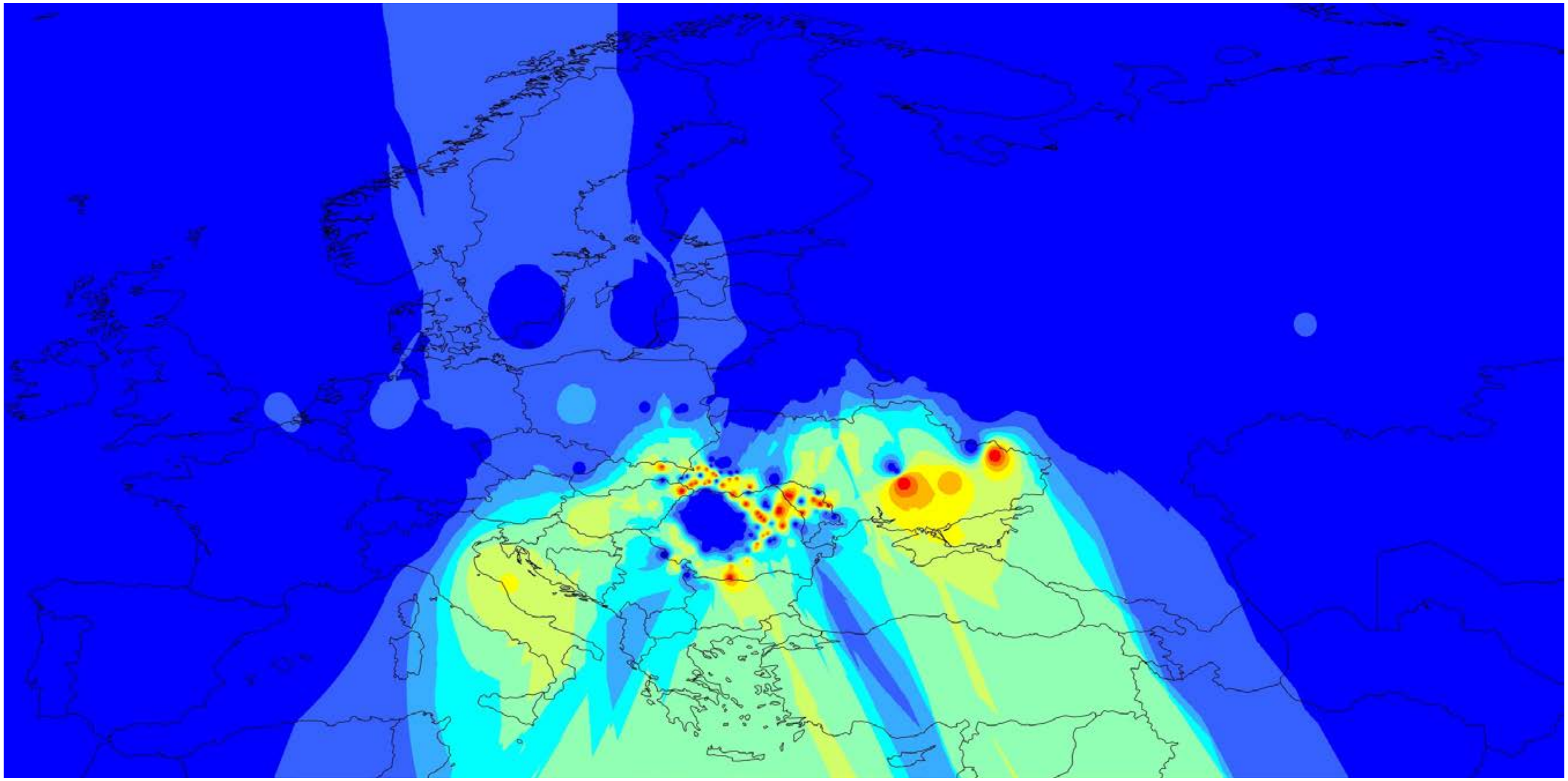

Species specific scale *T. dobrogicus*

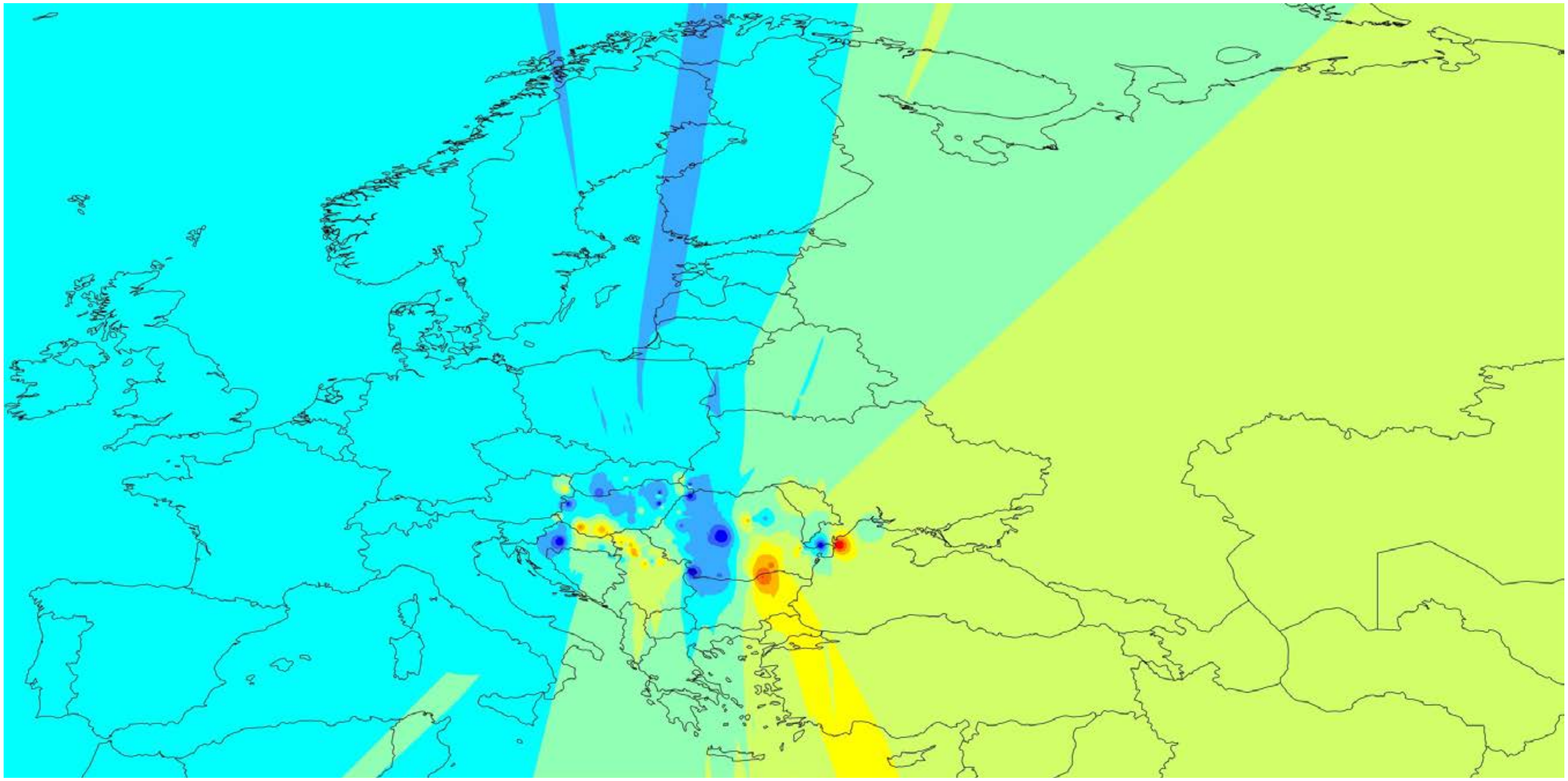

Species specific scale *T. karelinii* western species

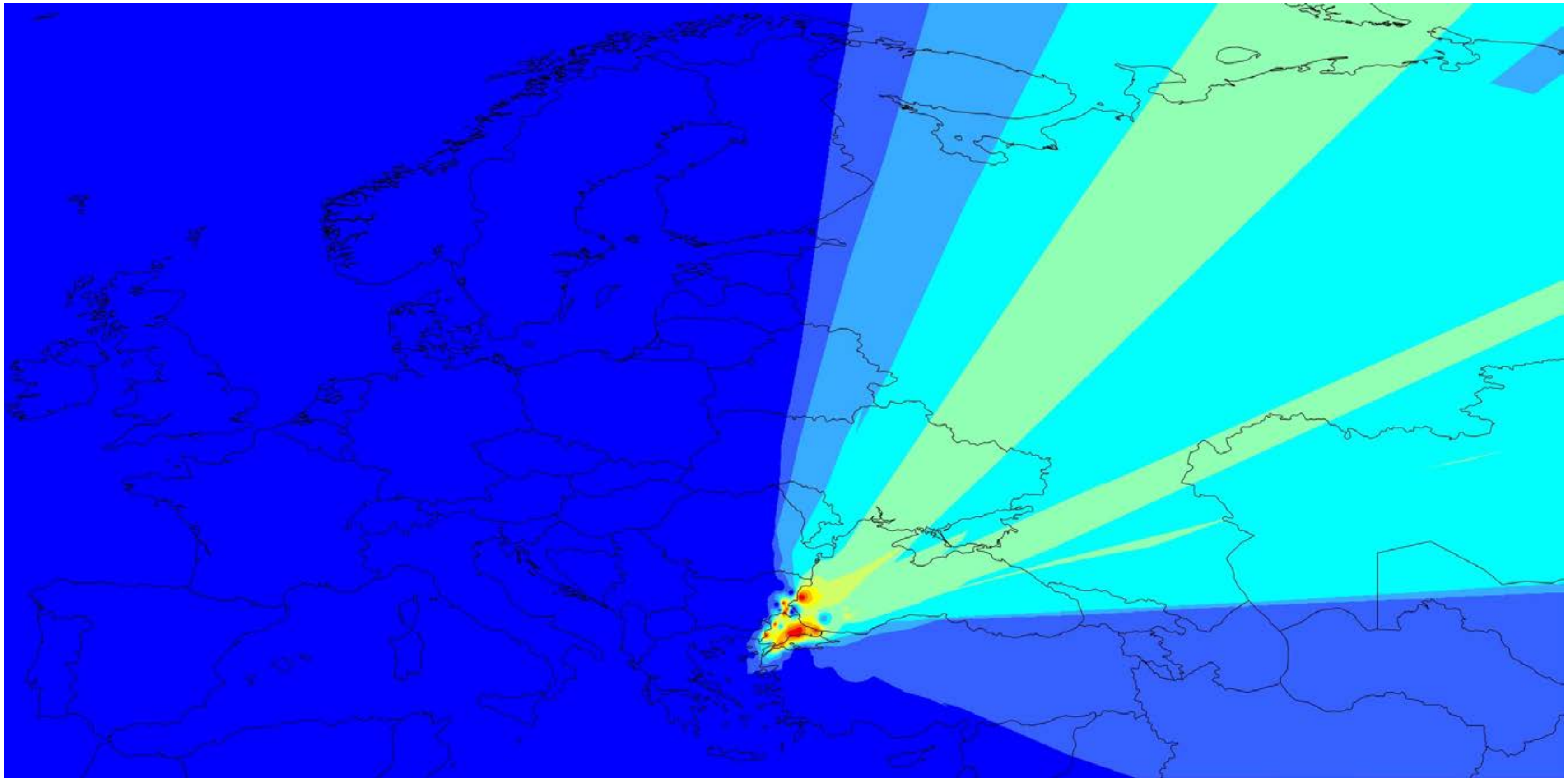

Species specific scale *T. karelinii* central species

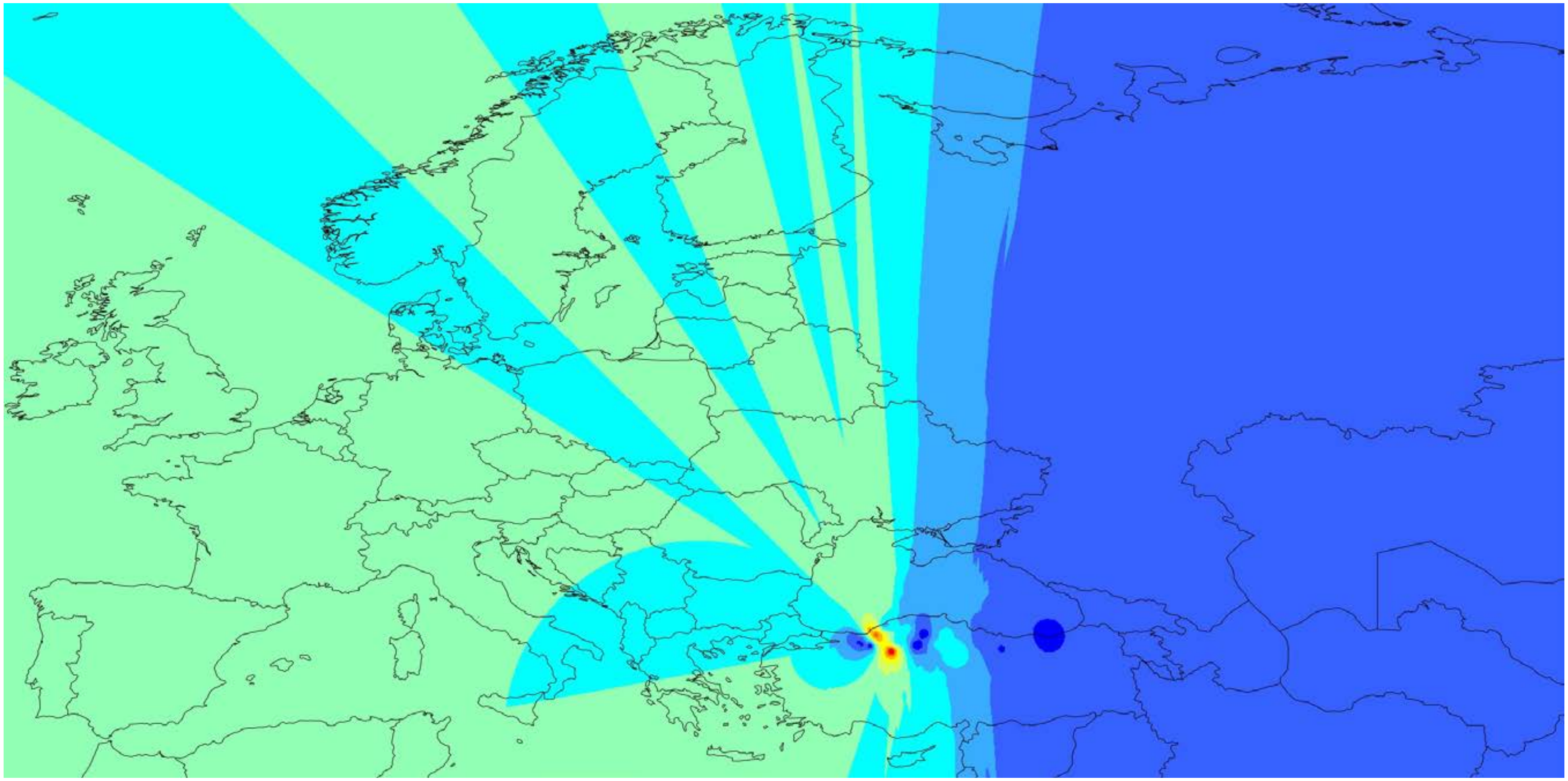

Species specific scale *T. karelinii* eastern species

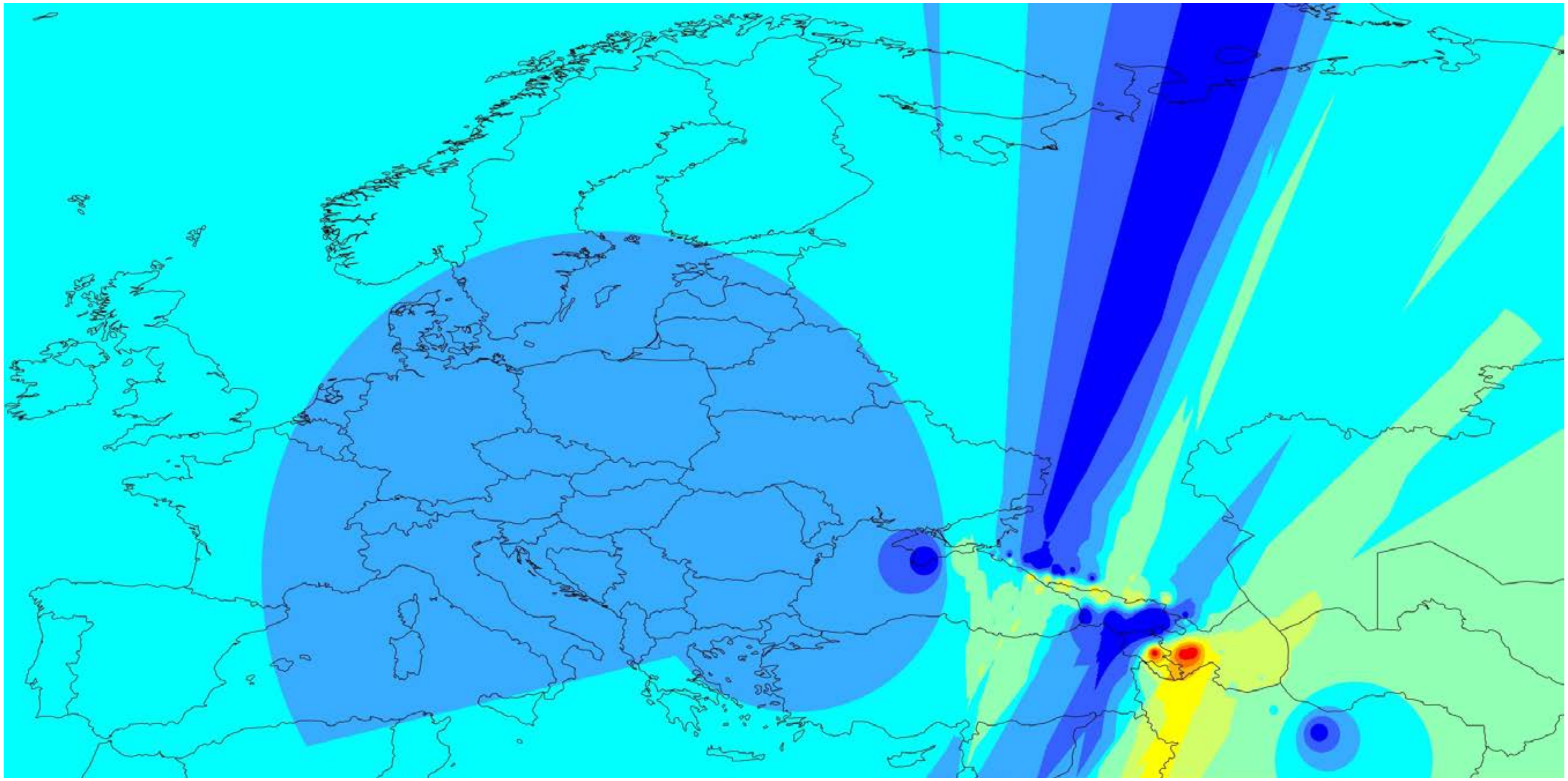

Species specific scale *T. macedonicus*

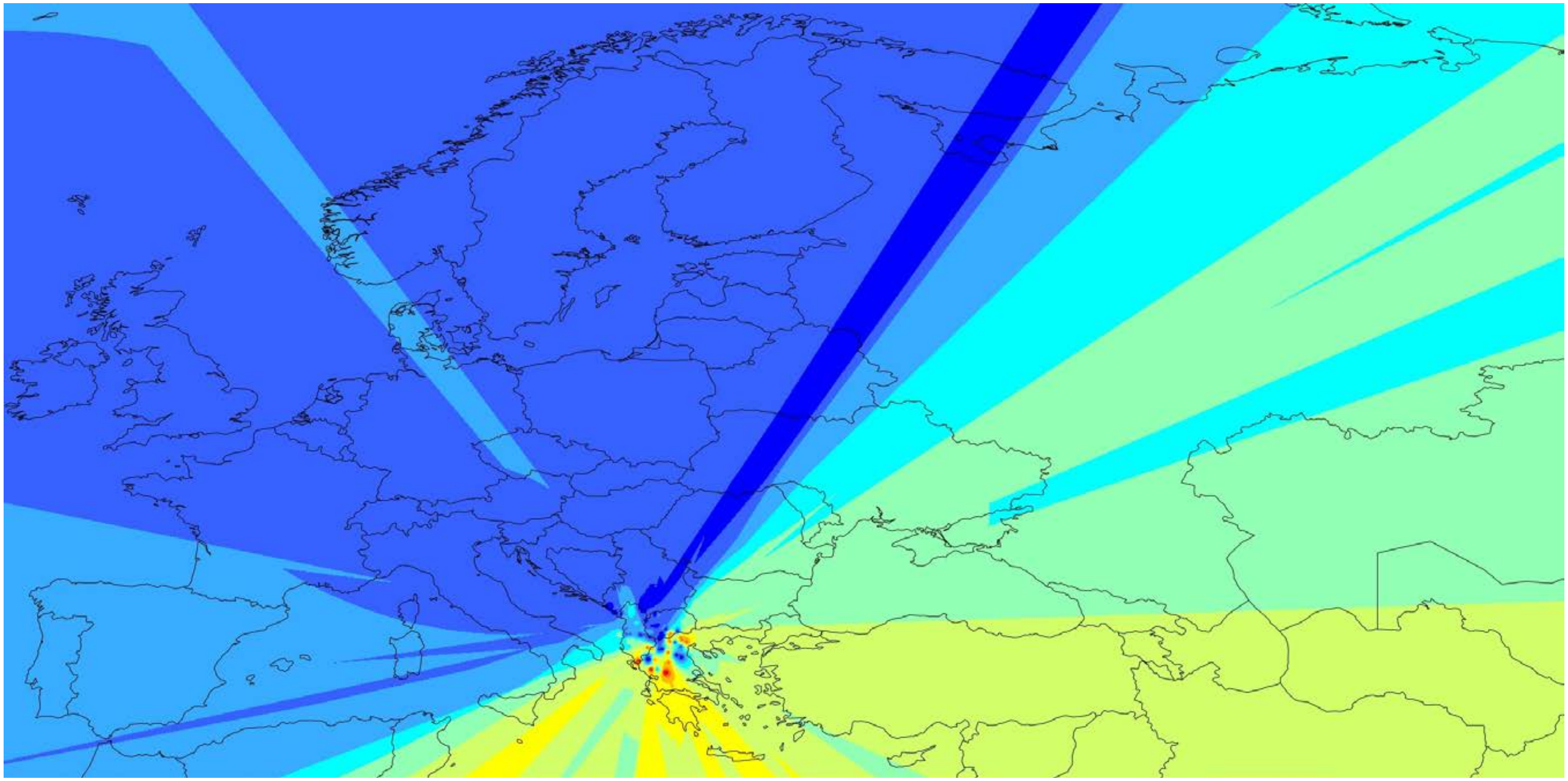

Species specific scale *T. marmoratus*

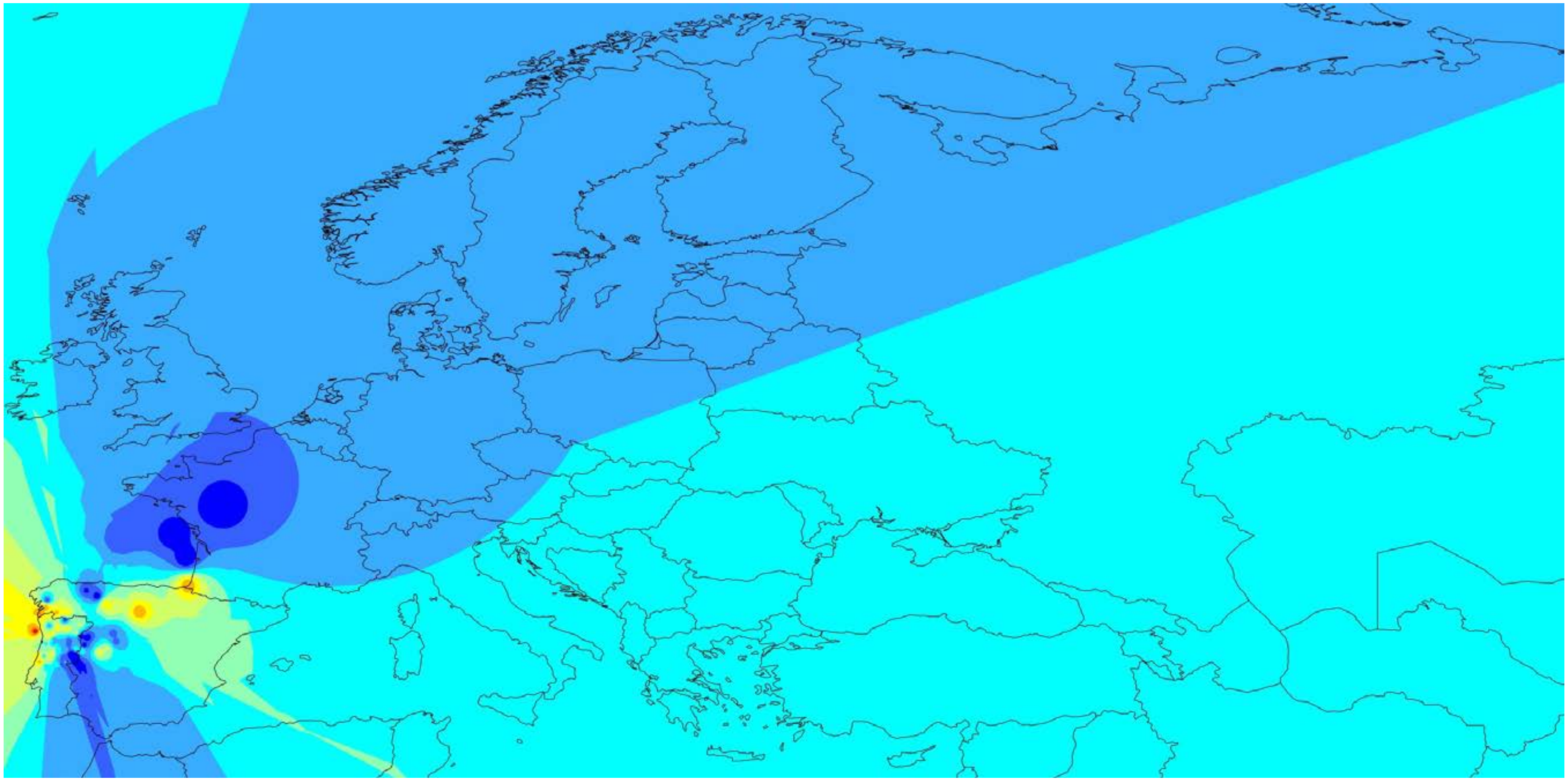

Species specific scale *T. pygmaeus*

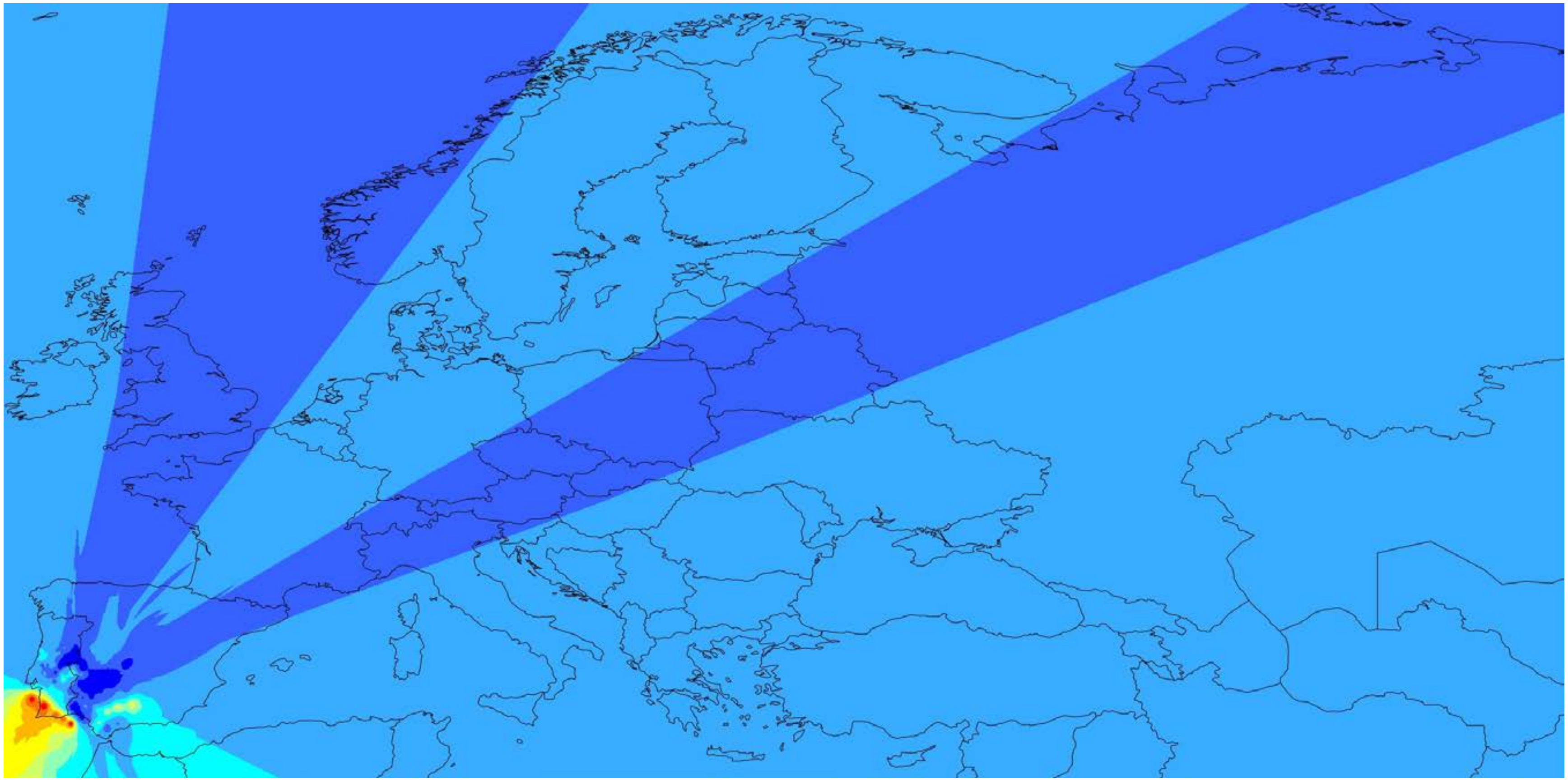

Supplement: Additional file 5 — Full genetic similarity maps The genetic (dis)similarity among populations within the different Triturus species, not cut according to the current species ranges. [file 1742-9994-10-13-S5.pdf]
