# Supplementary material for: Tracing glacial refugia of Triturus newts based on mitochondrial DNA phylogeography and species distribution modeling
Source: Front Zool. 2013 Mar 20;10:13. doi: 10.1186/1742-9994-10-13 (PMC3608019; doi:10.1186/1742-9994-10-13)

Additional file 7. Full species distribution models. The species distribution model of each *Triturus* species projected for Last Glacial Maximum and current climate conditions, not cut according to the current species ranges. For each species, its ecological niche model was projected on Last Glacial Maximum (both the MIROC and CCSM model) and current climate layers. Warmer colors refer to a higher predicted suitability.

MIROC *T. carnifex*

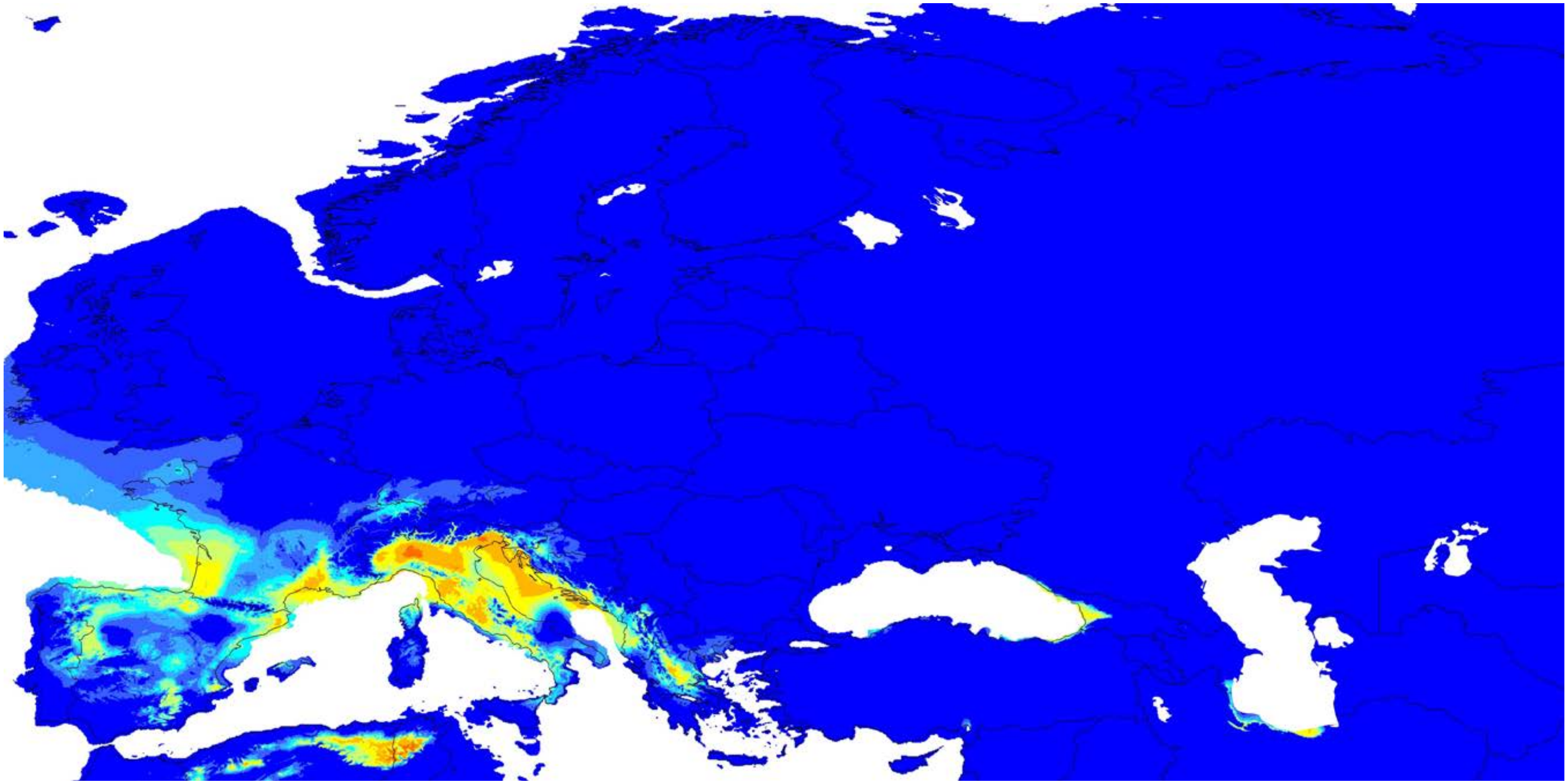

MIROC *T. cristatus*

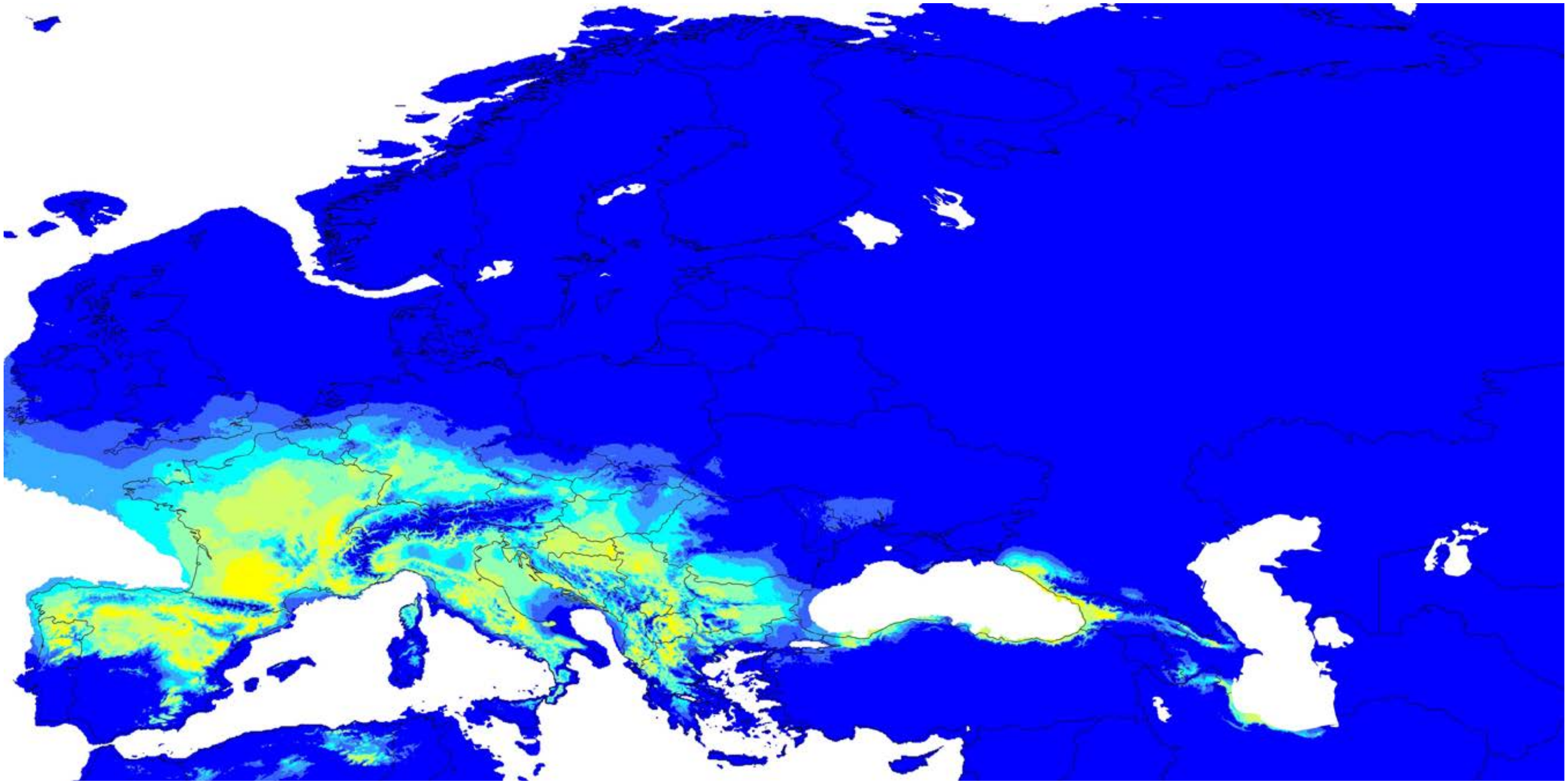

MIROC *T. dobrogicus*

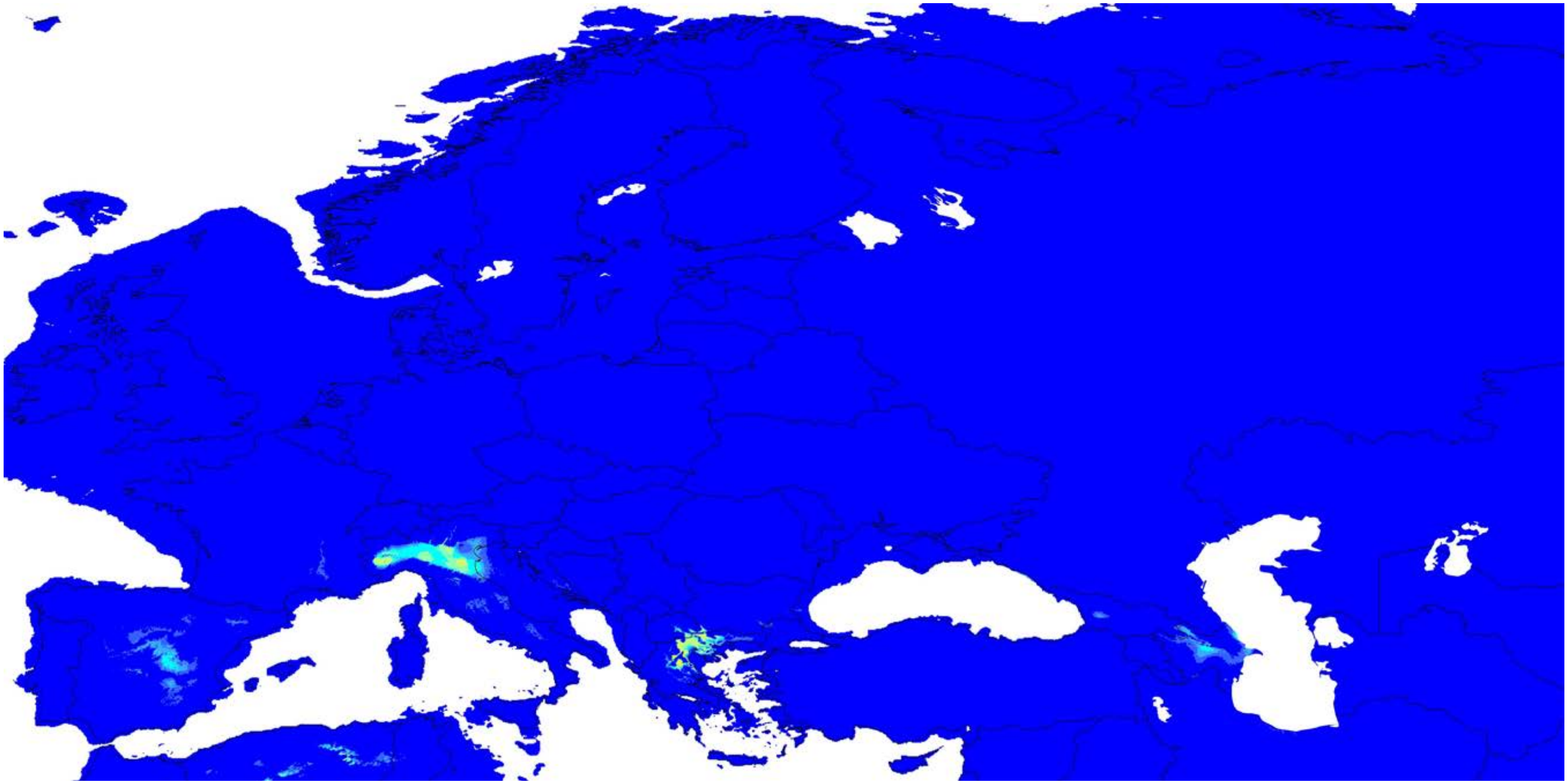

MIROC *T. karelinii* western species

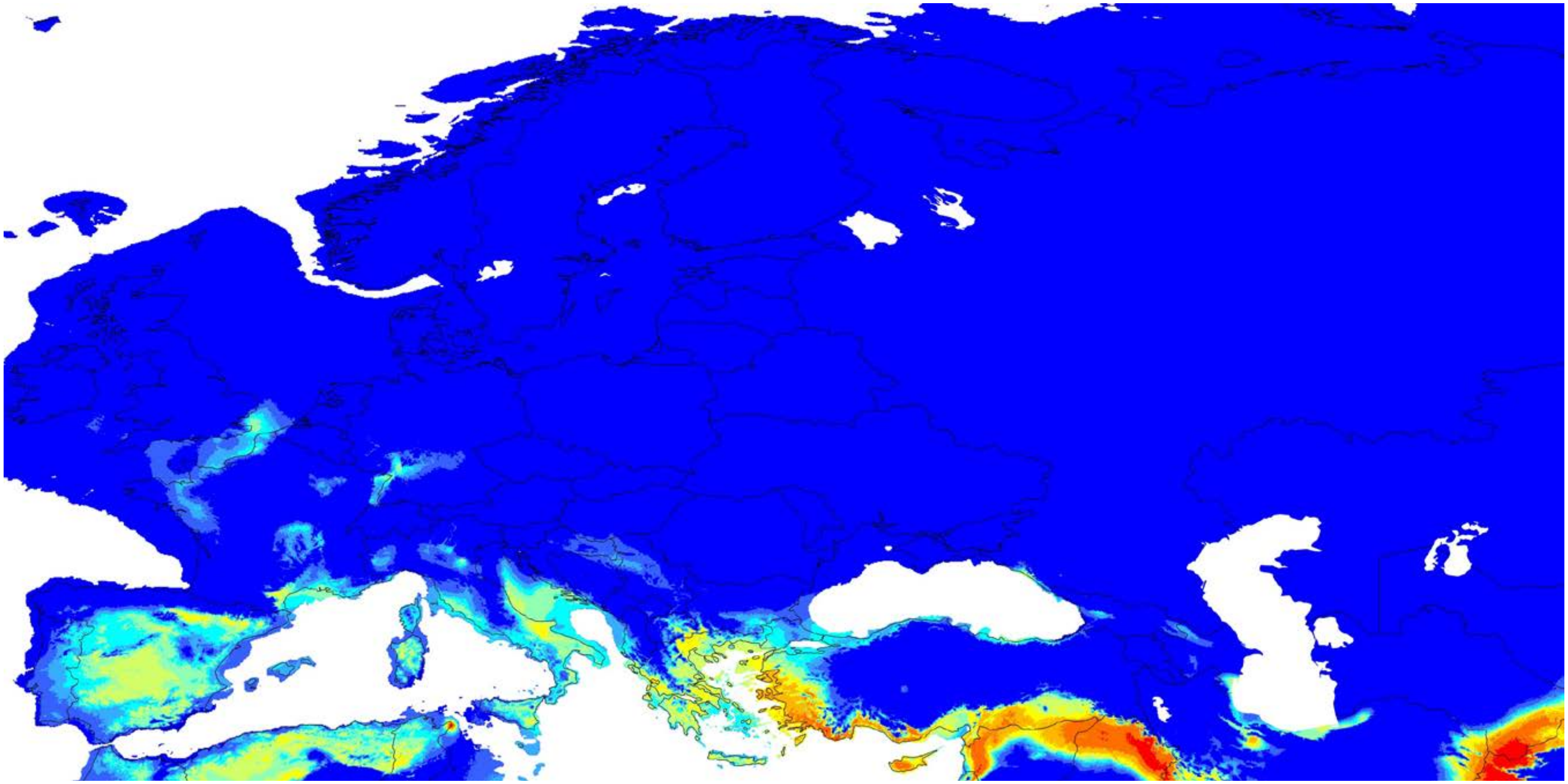

MIROC *T. karelinii* central species

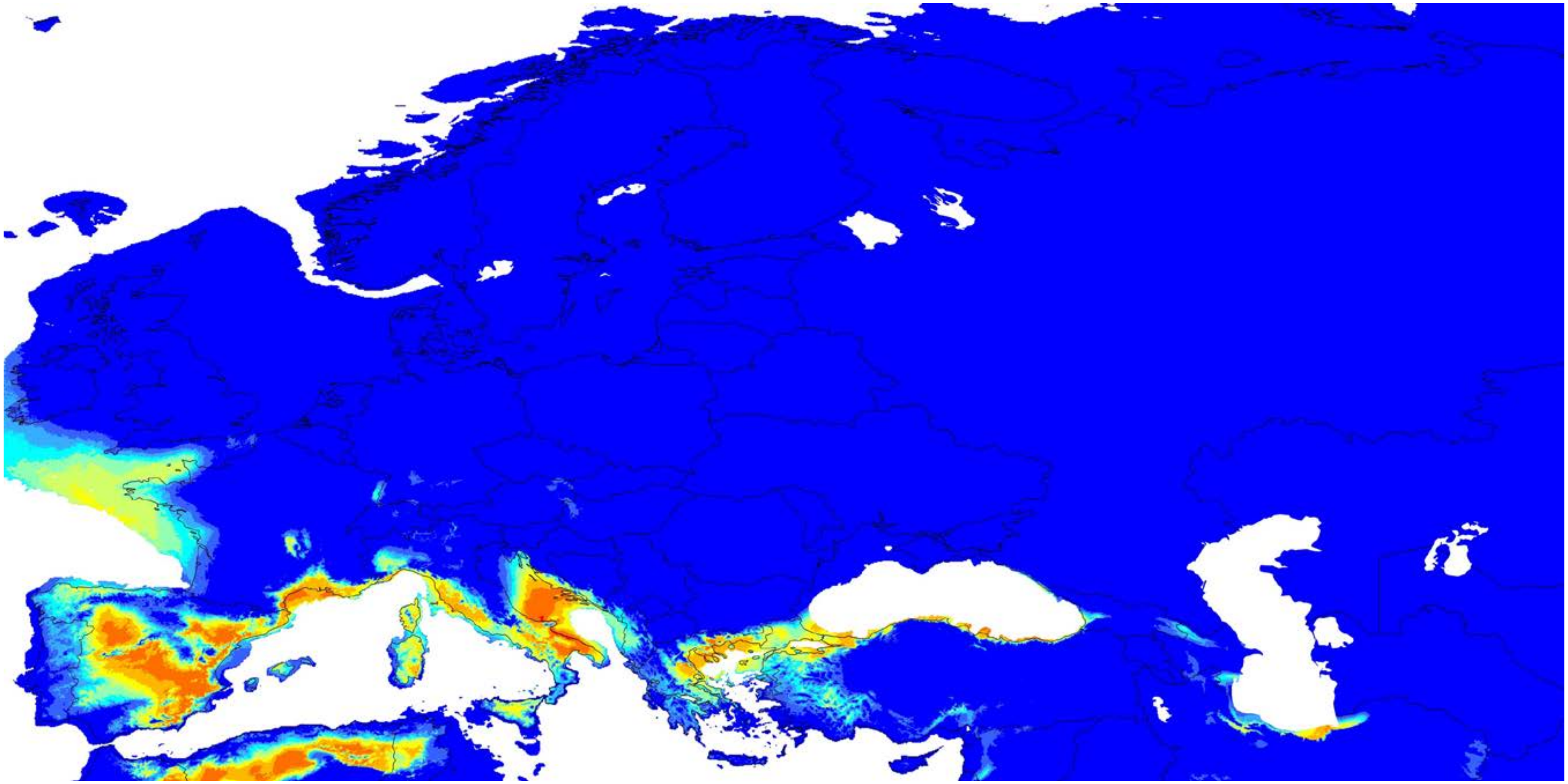

MIROC *T. karelinii* eastern species

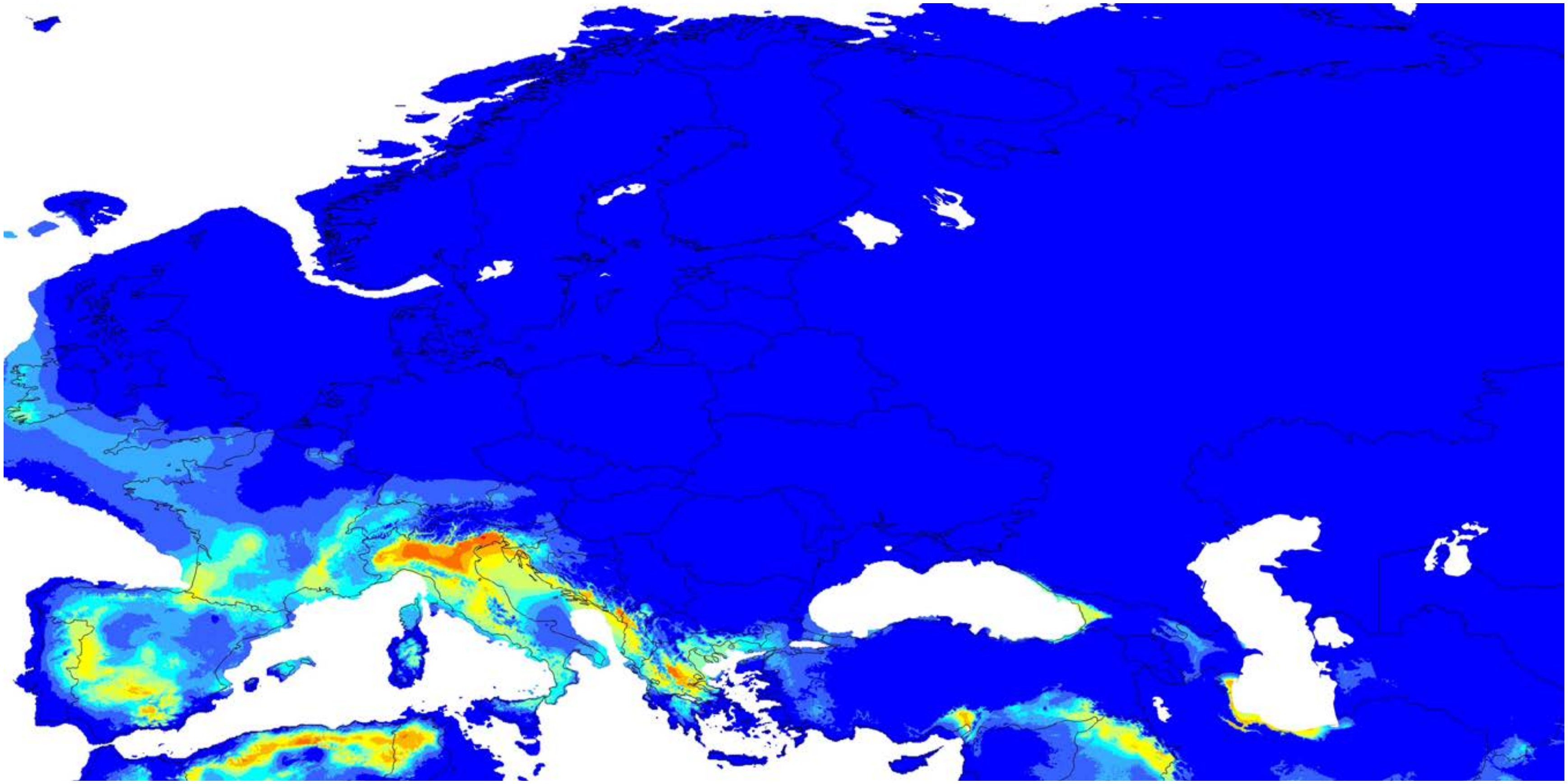

MIROC *T. macedonicus*

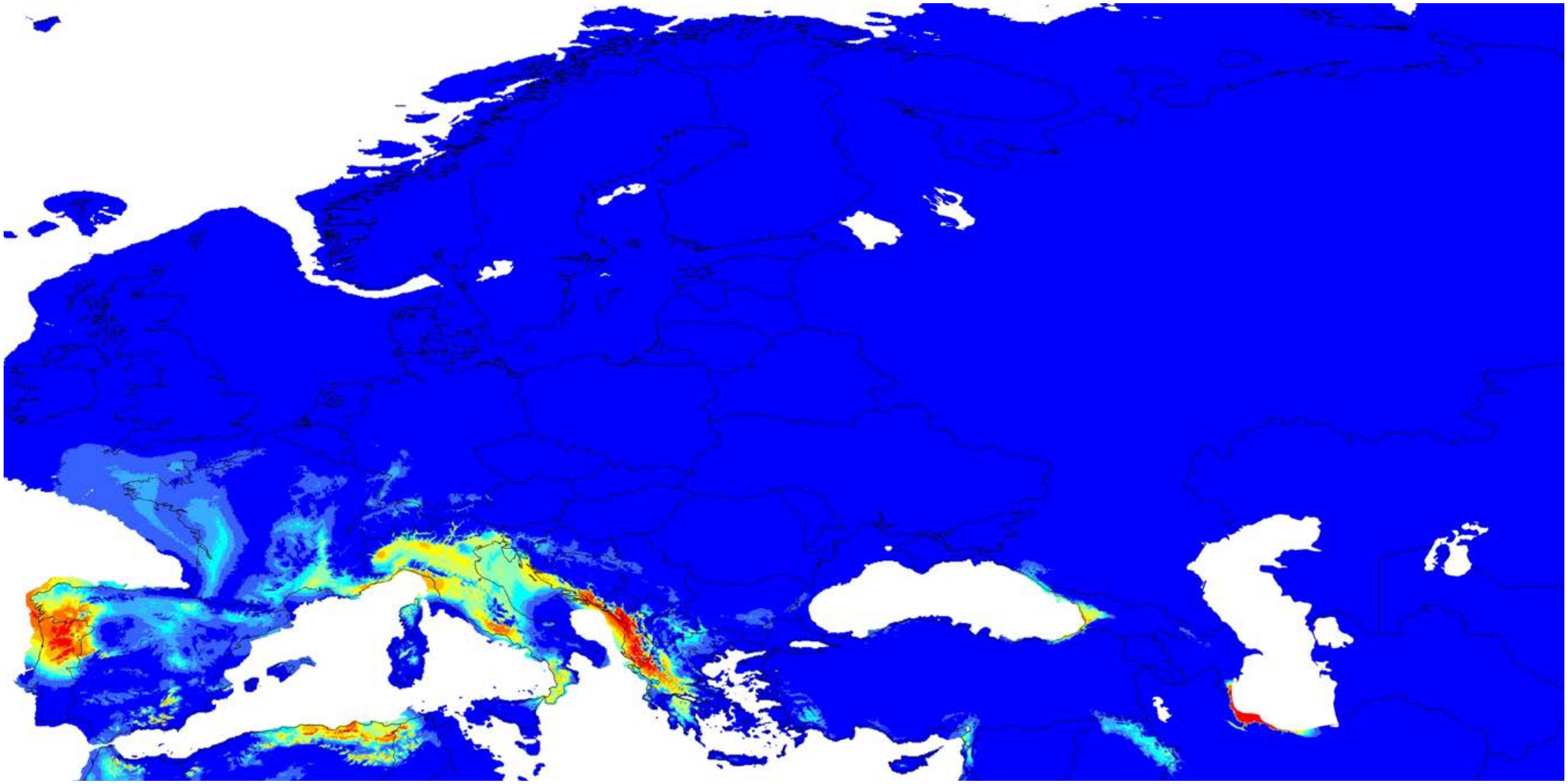

MIROC *T. marmoratus*

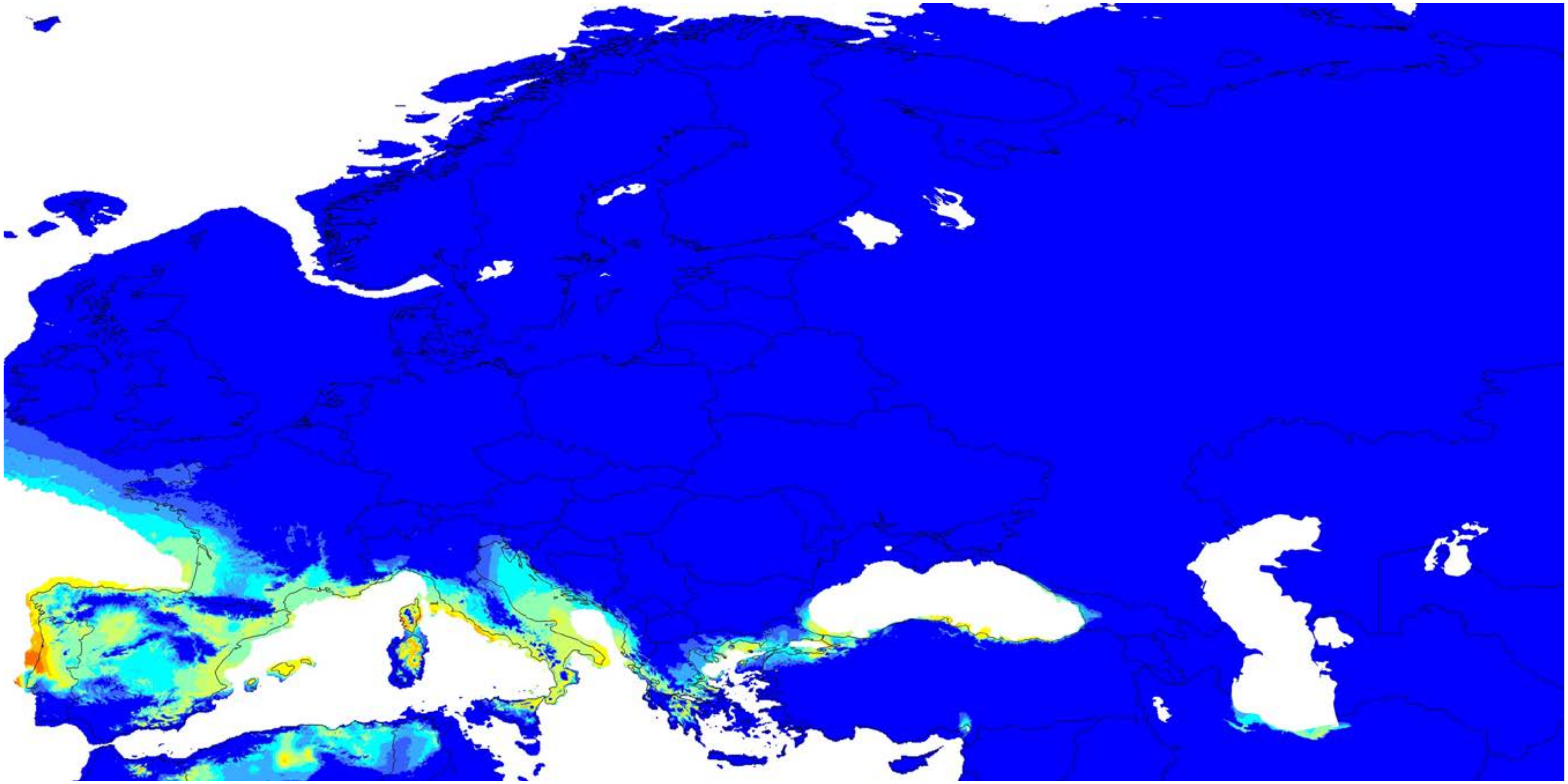

MIROC *T. pygmaeus*

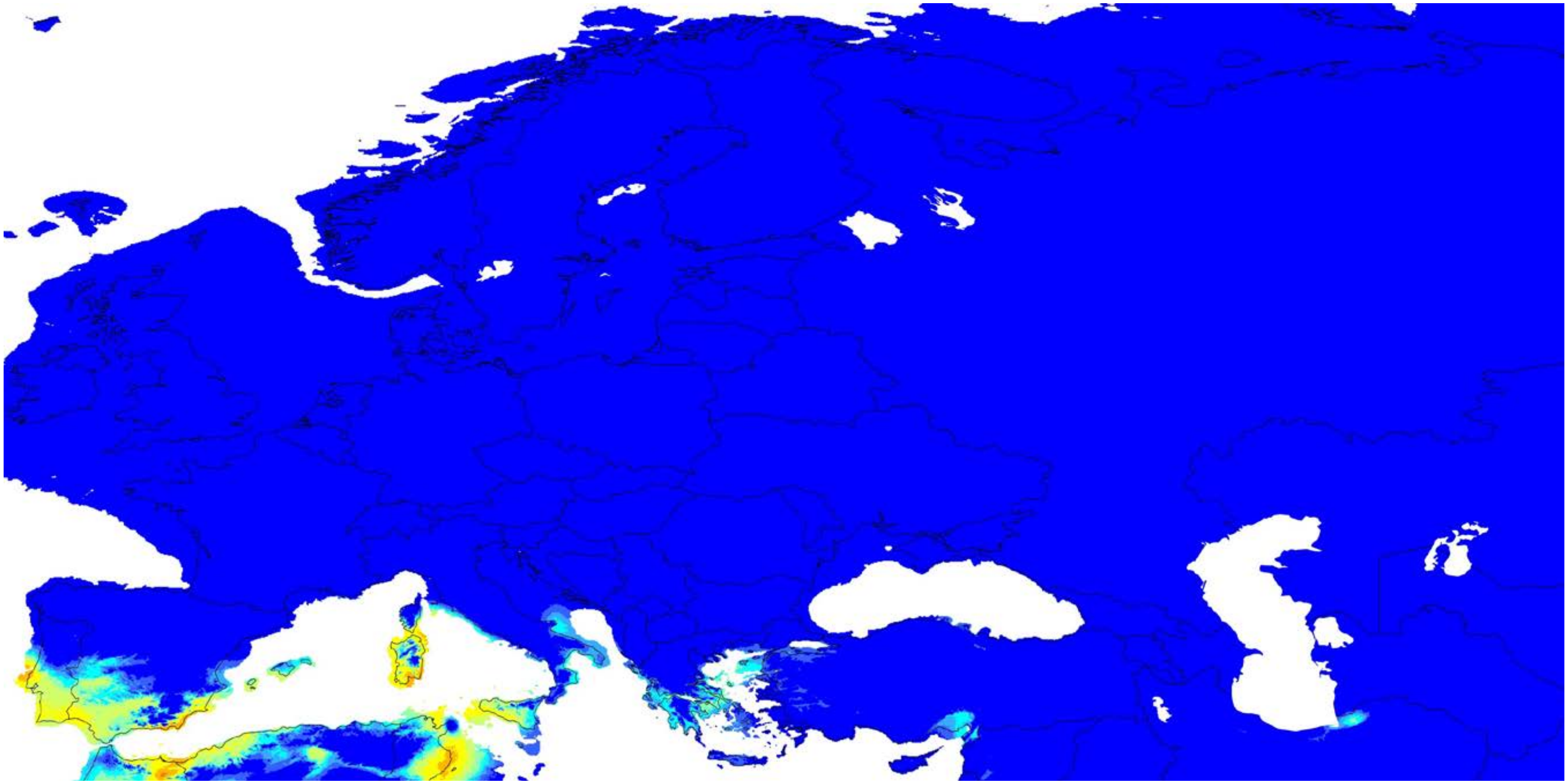

CCSM *T. carnifex*

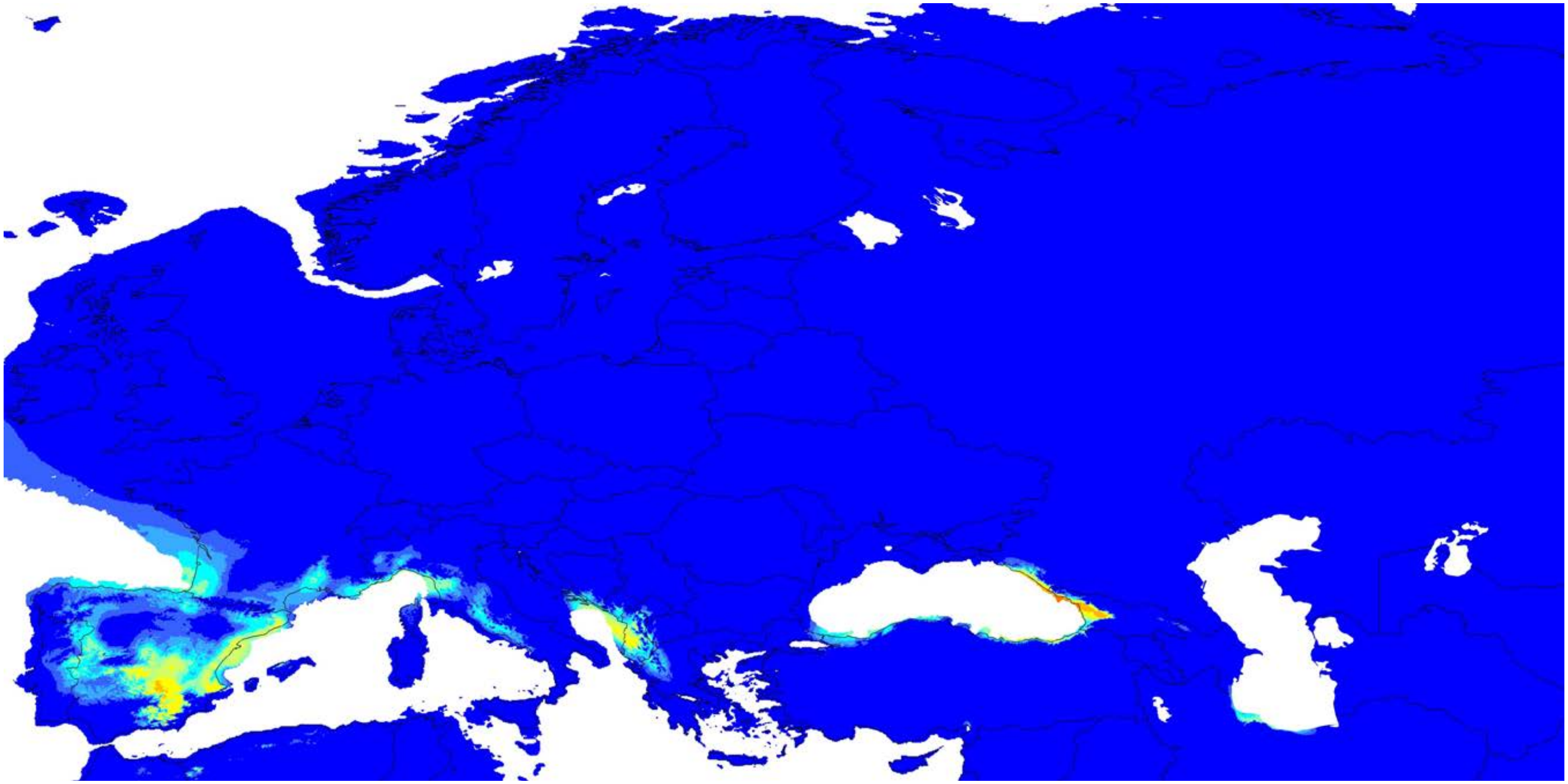

CCSM *T. cristatus*

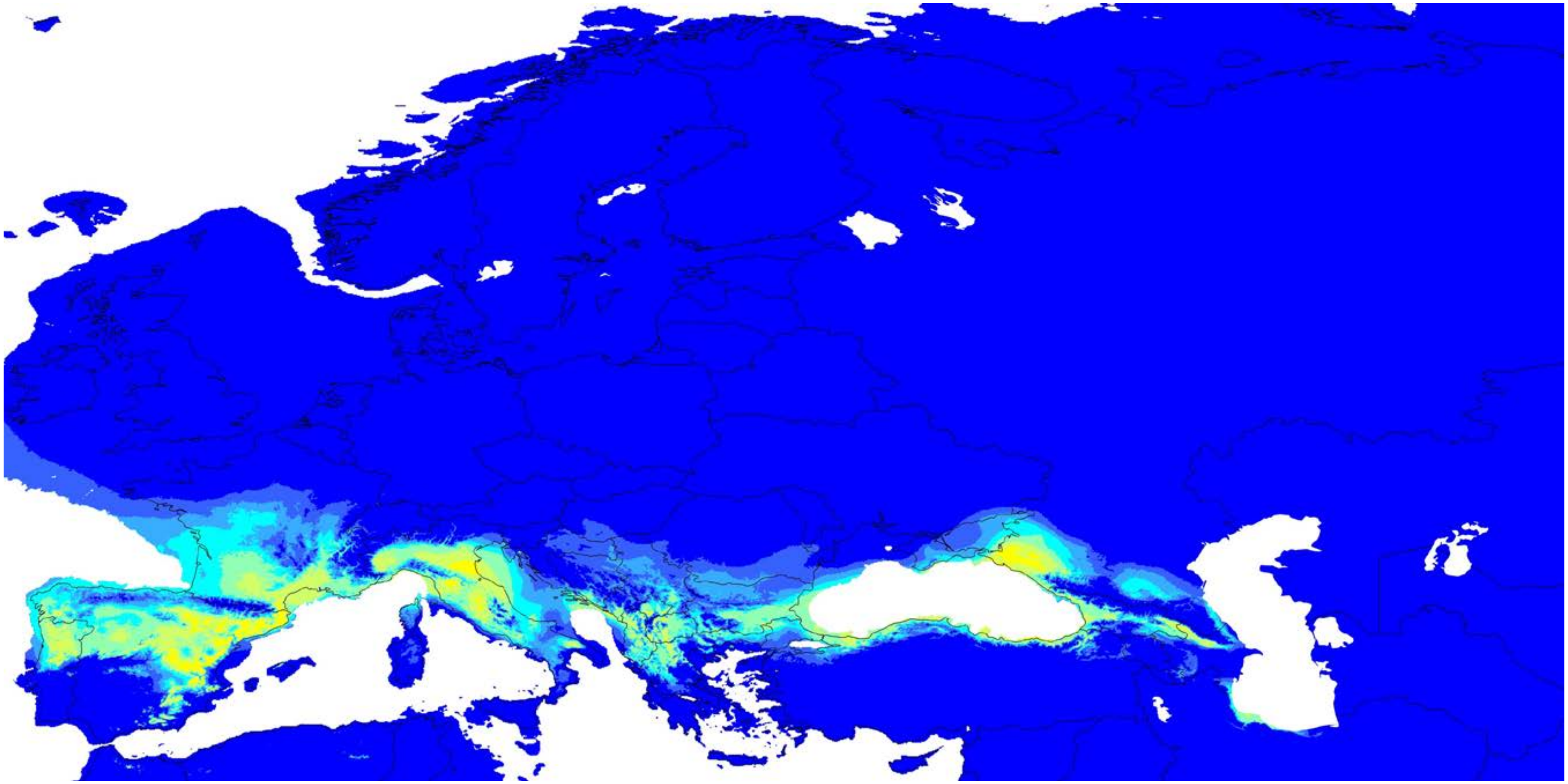

CCSM *T. dobrogicus*

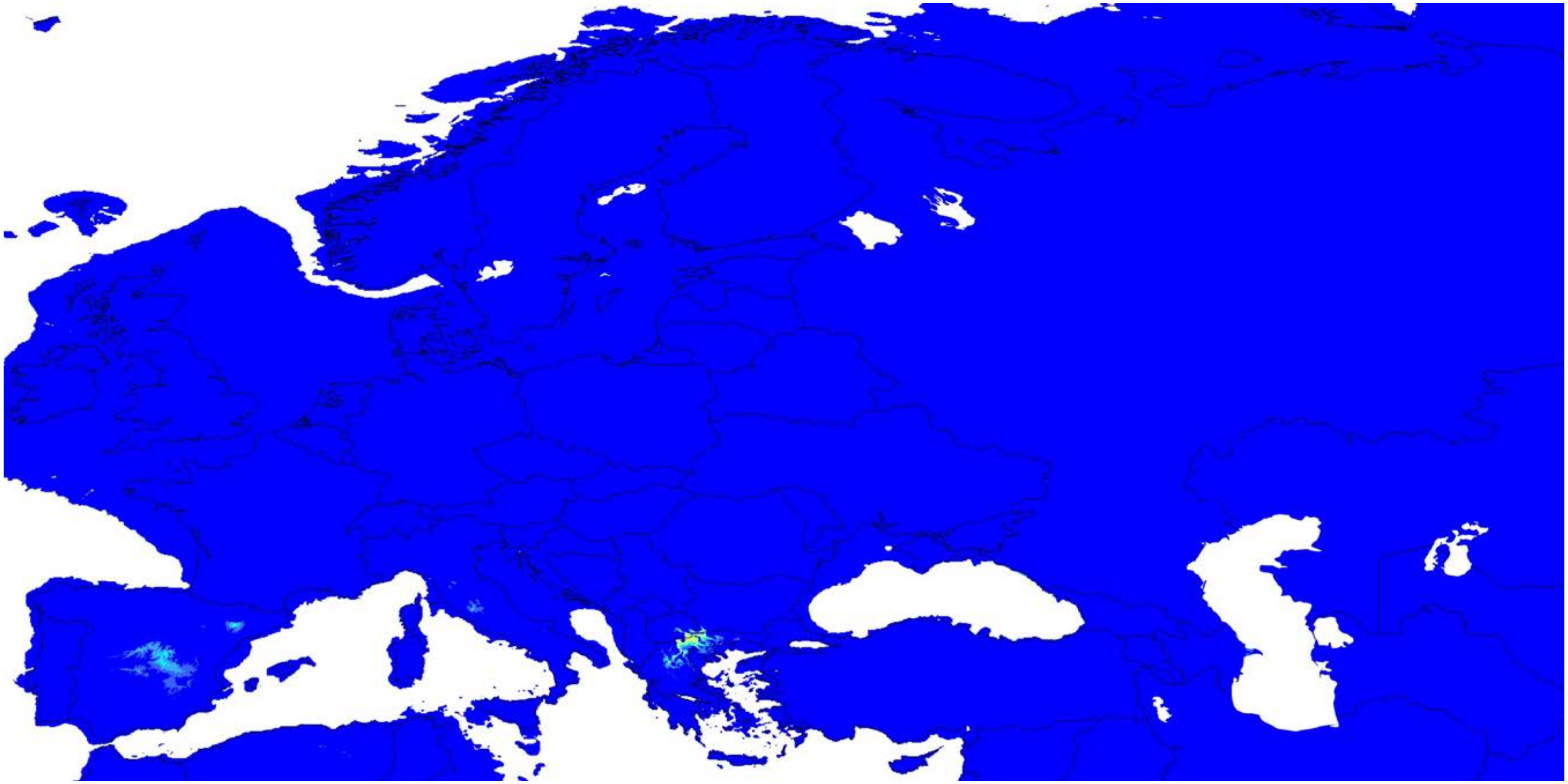

CCSM *T. karelinii* western species

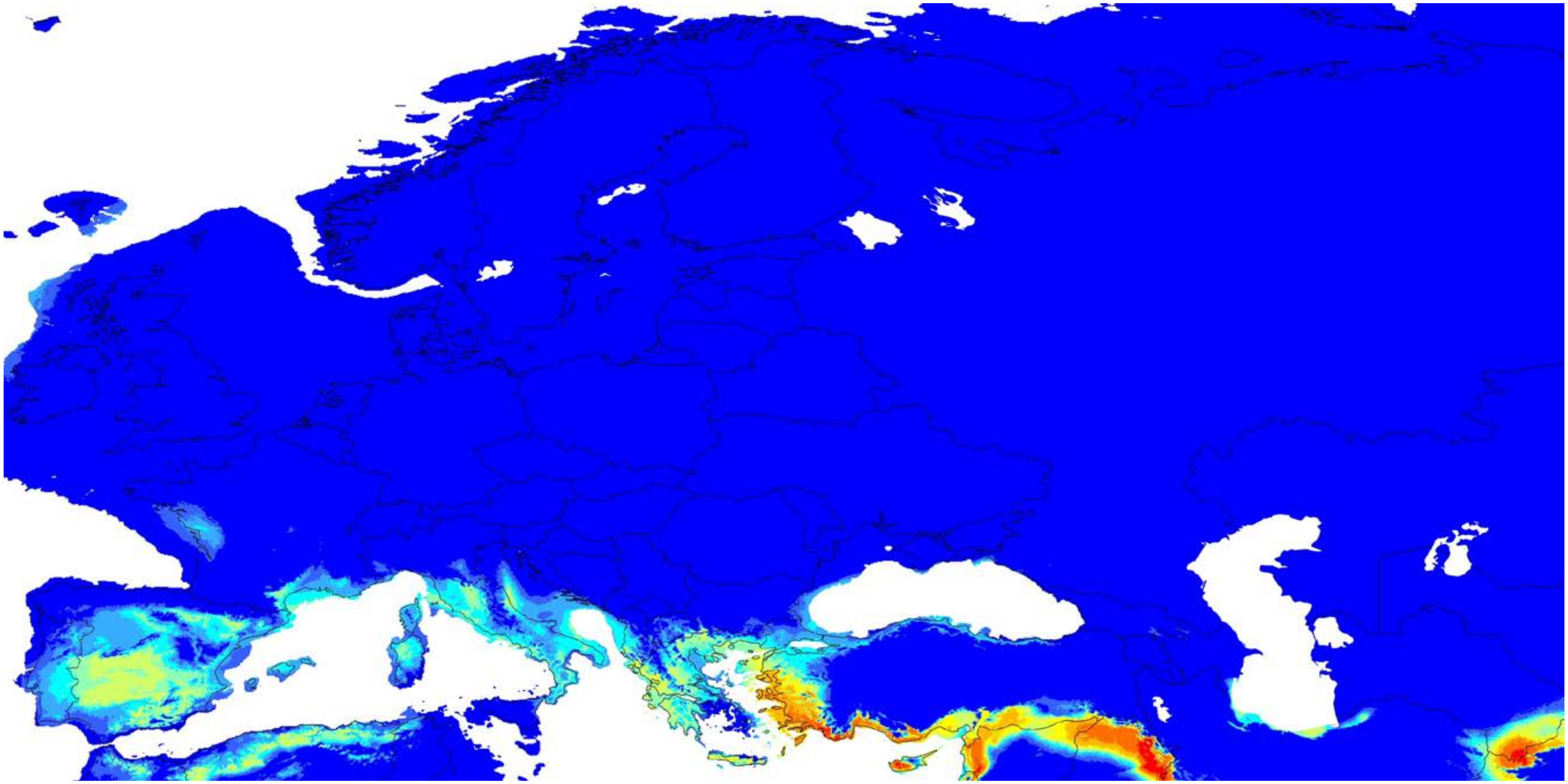

CCSM *T. karelinii* central species

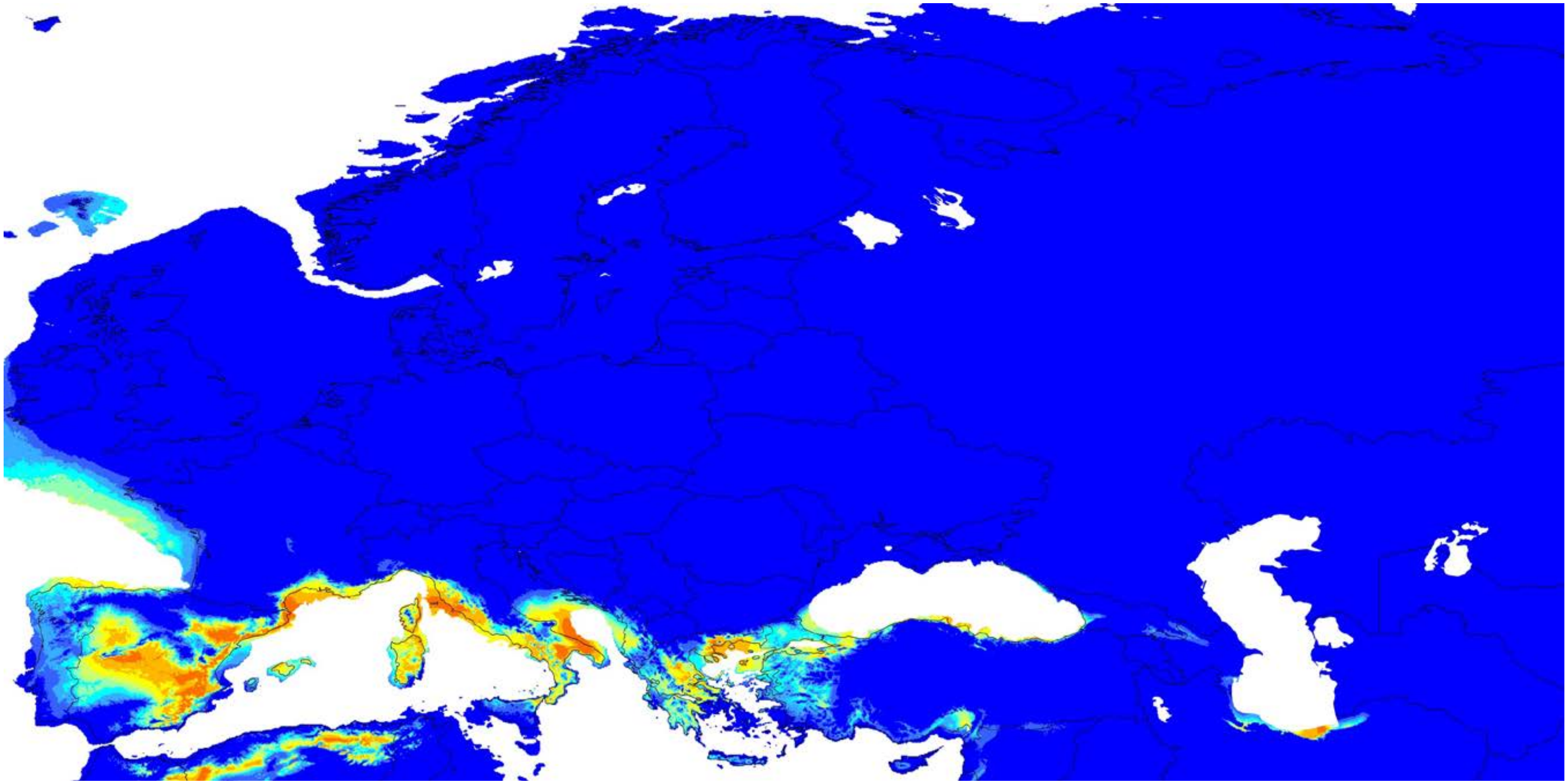

CCSM *T. karelinii* eastern species

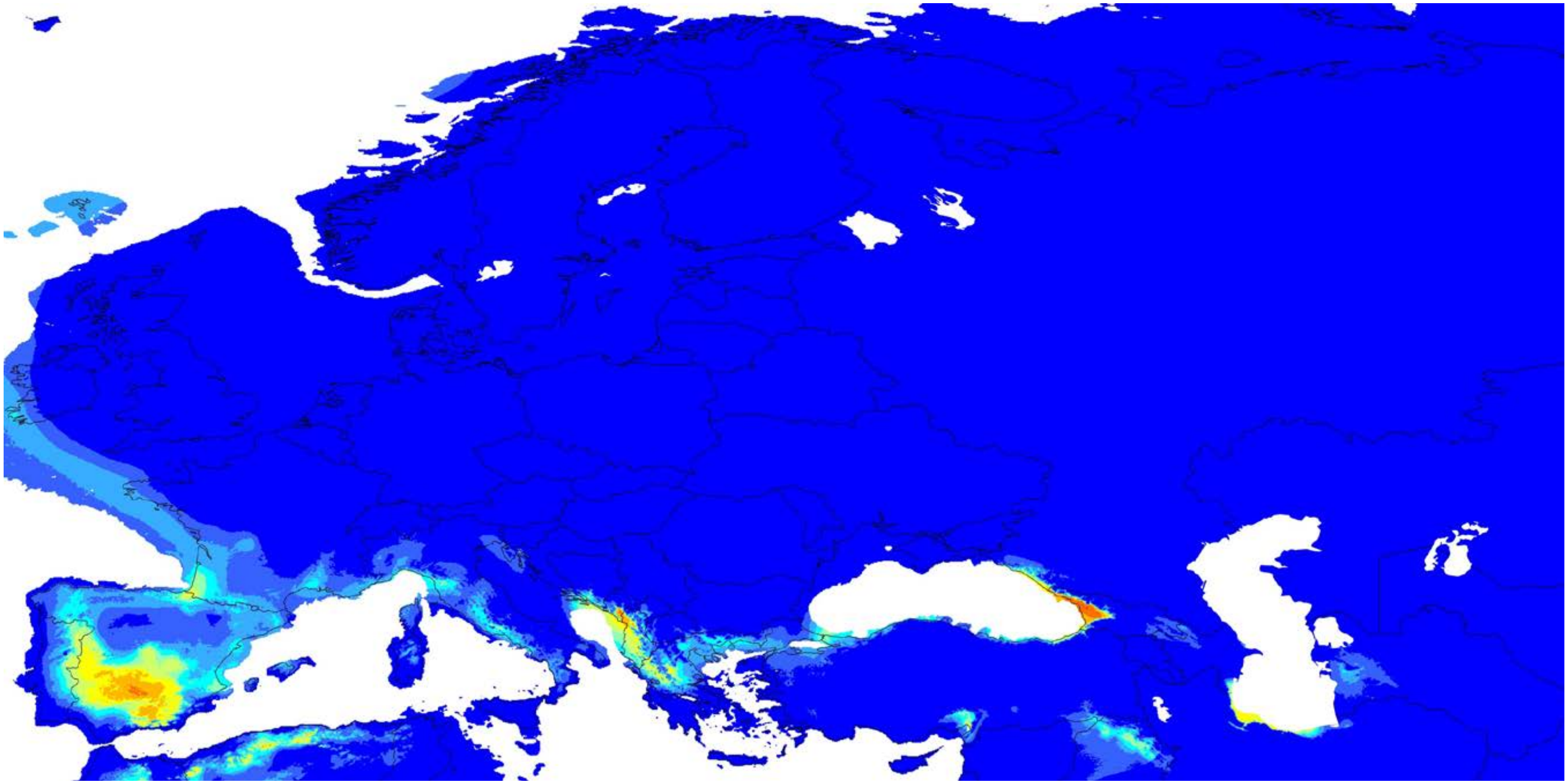

CCSM *T. macedonicus*

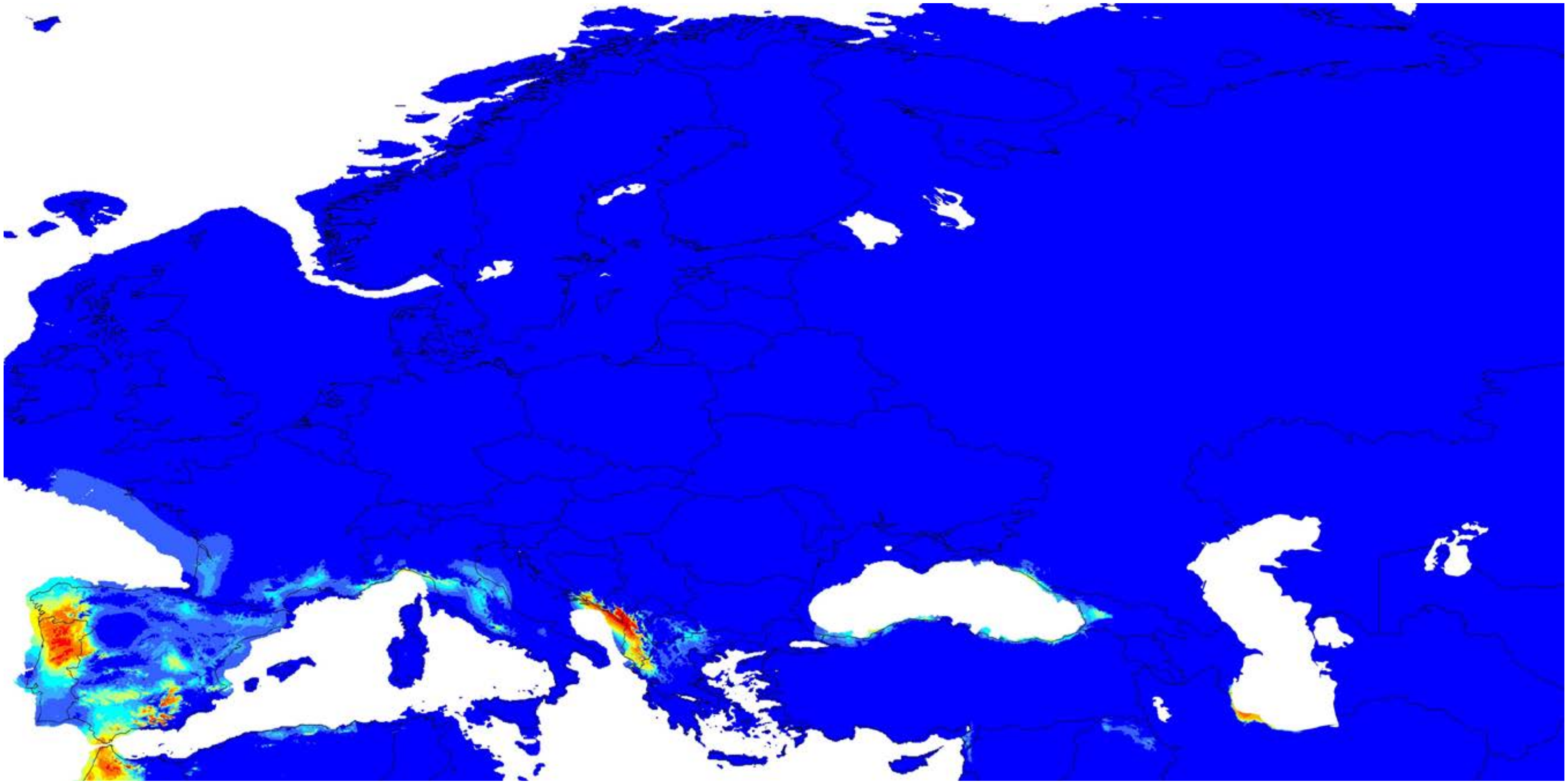

CCSM *T. marmoratus*

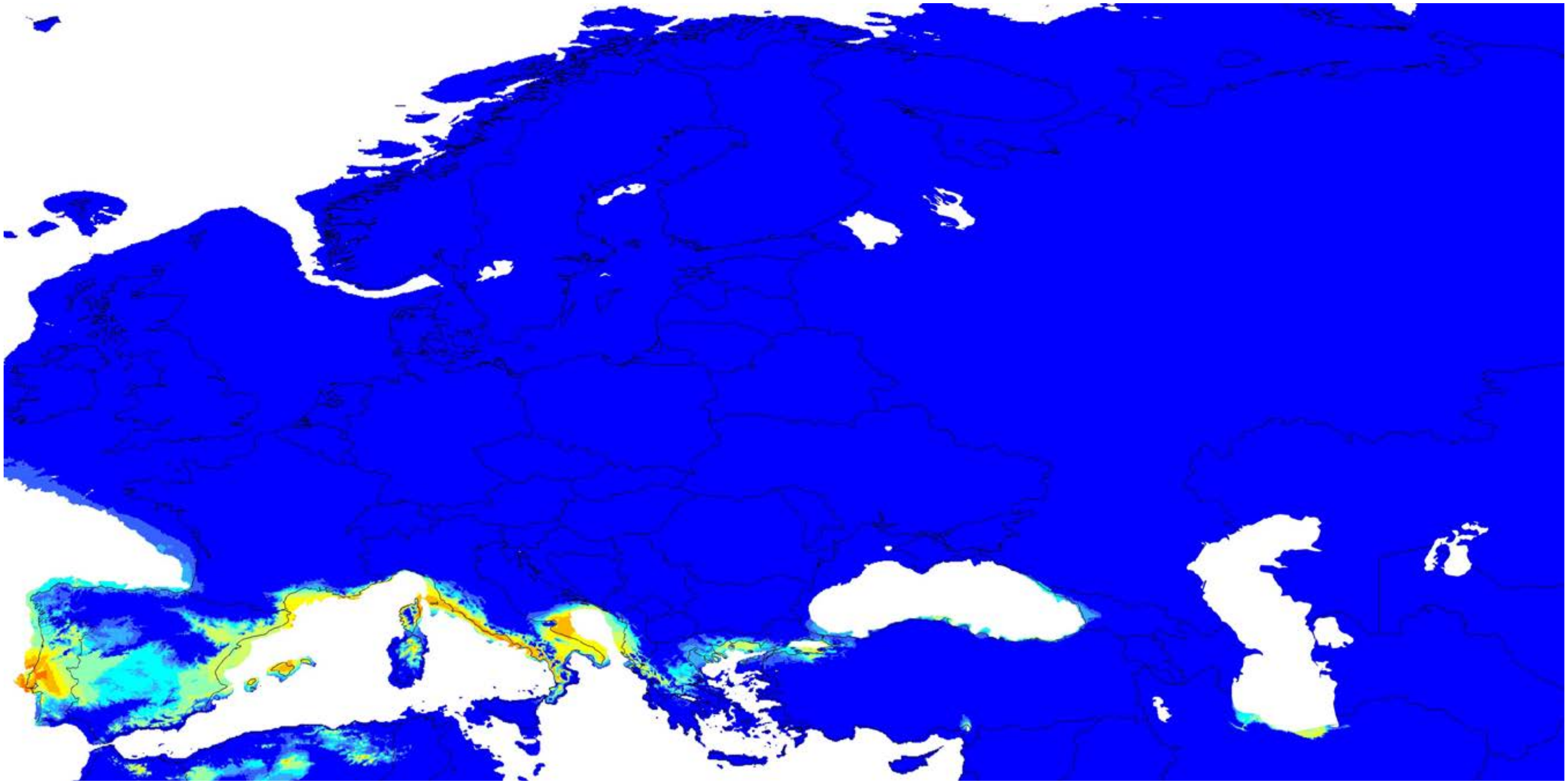

CCSM *T. pygmaeus*

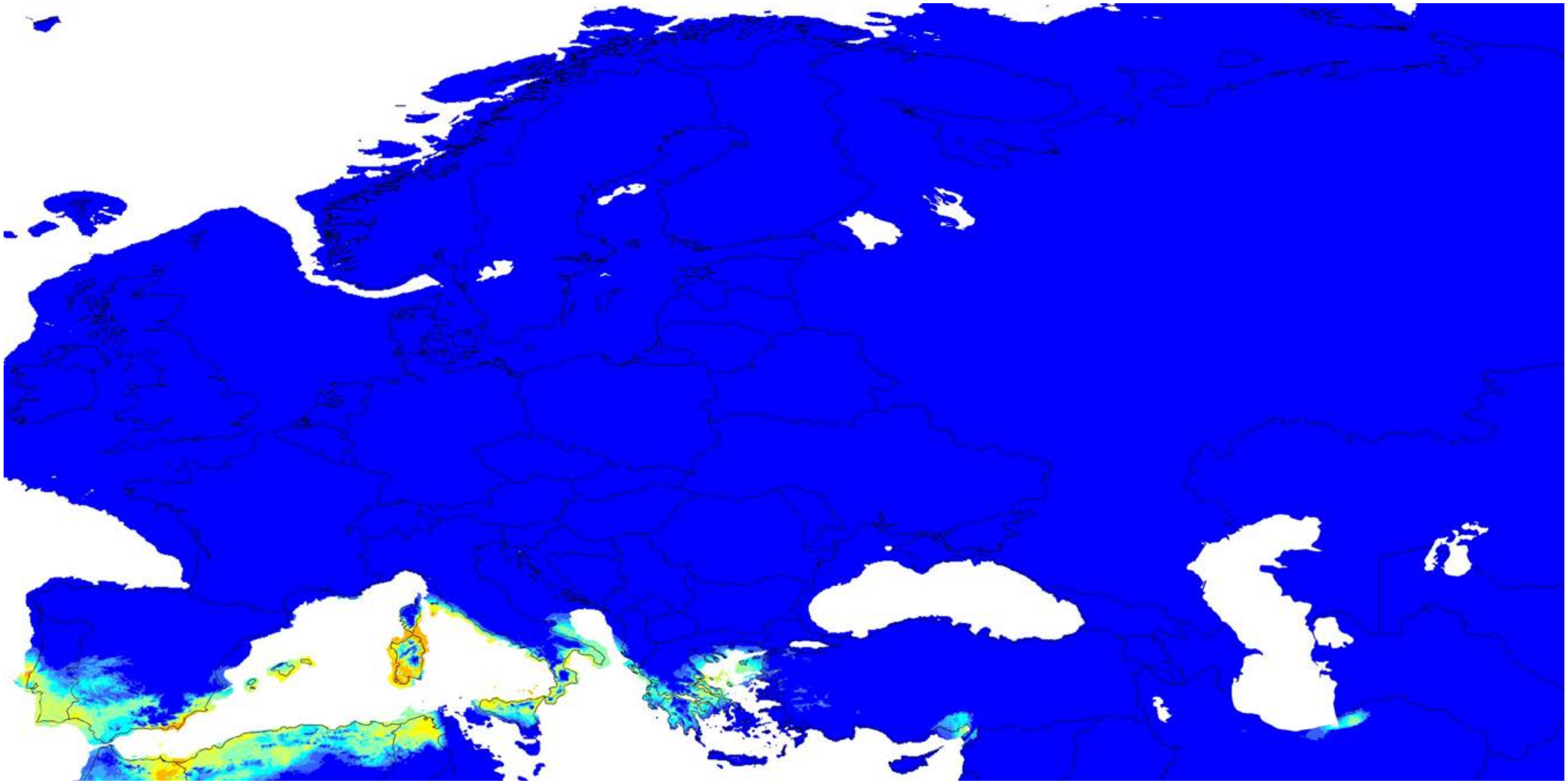

CURRENT *T. carnifex*

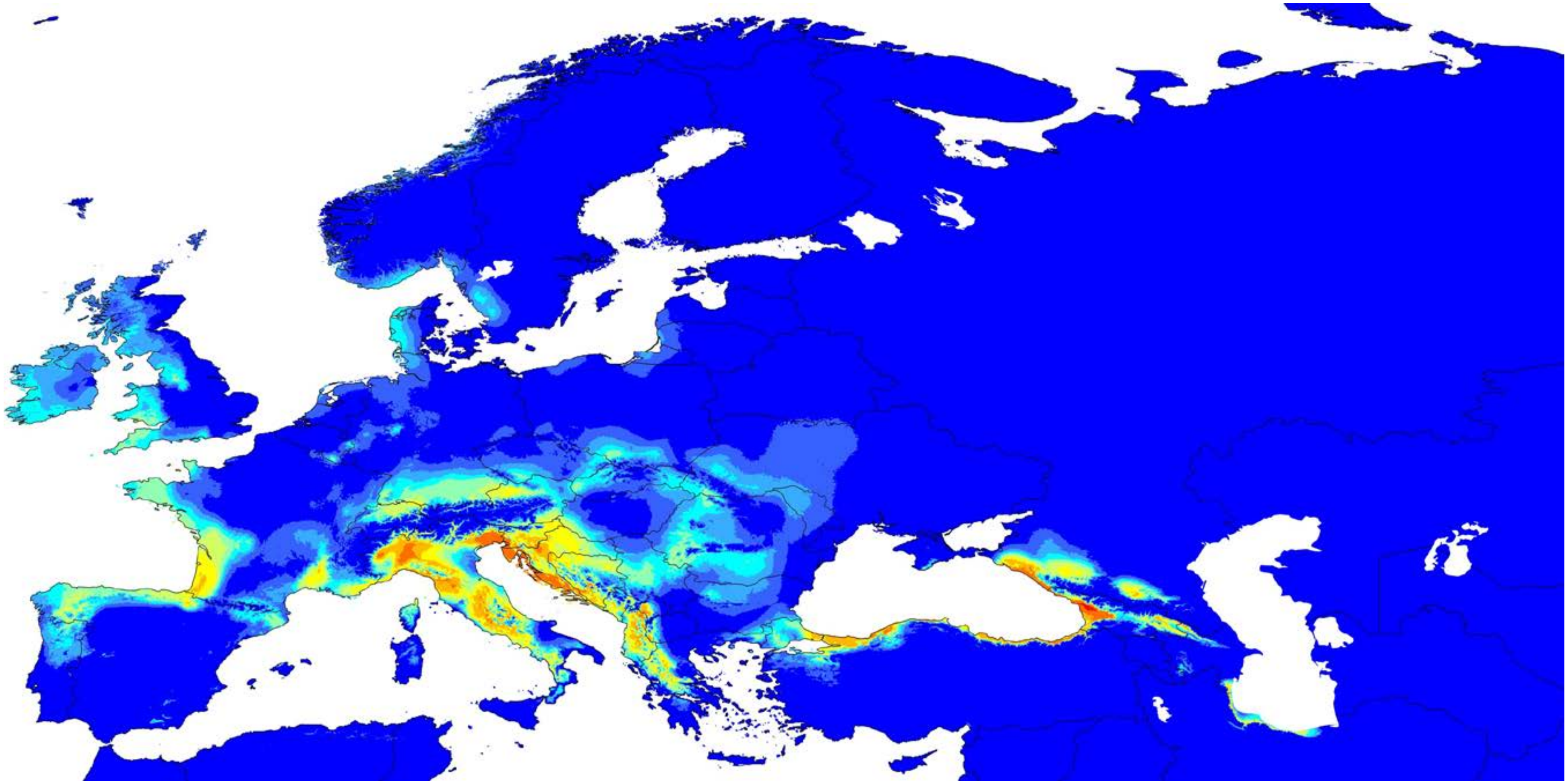

CURRENT *T. cristatus*

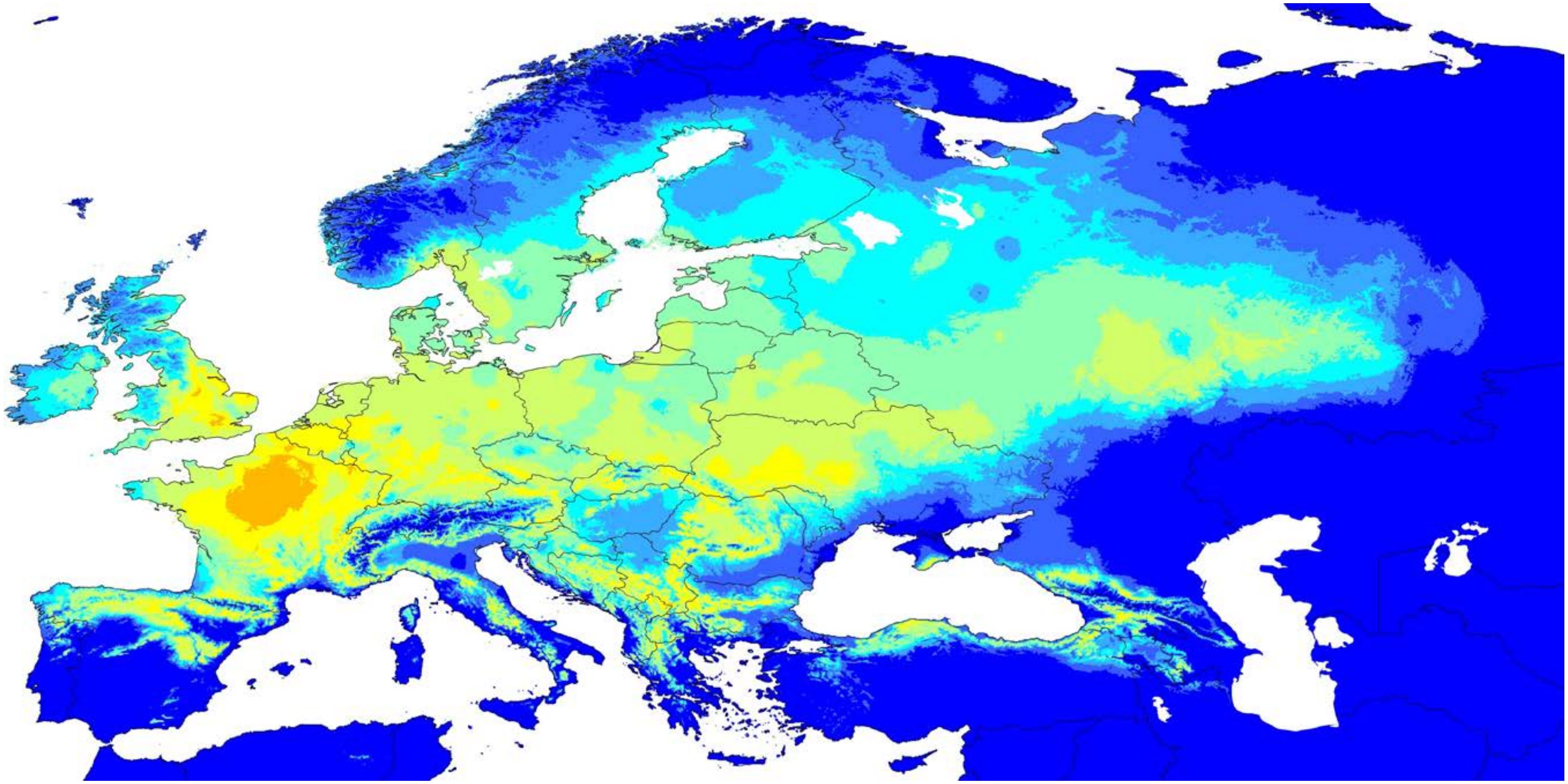

CURRENT *T. dobrogicus*

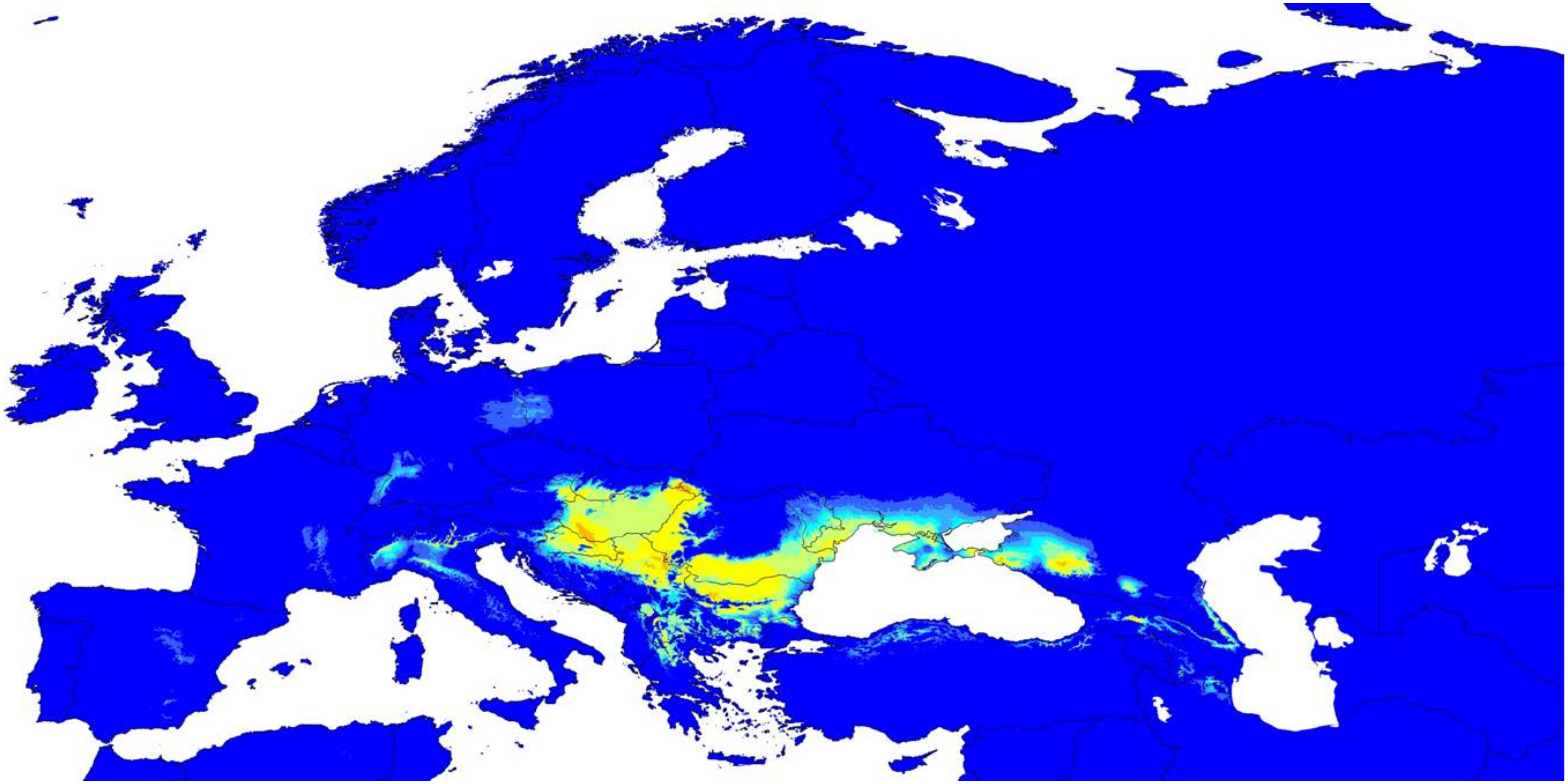

CURRENT *T. karelinii* western species

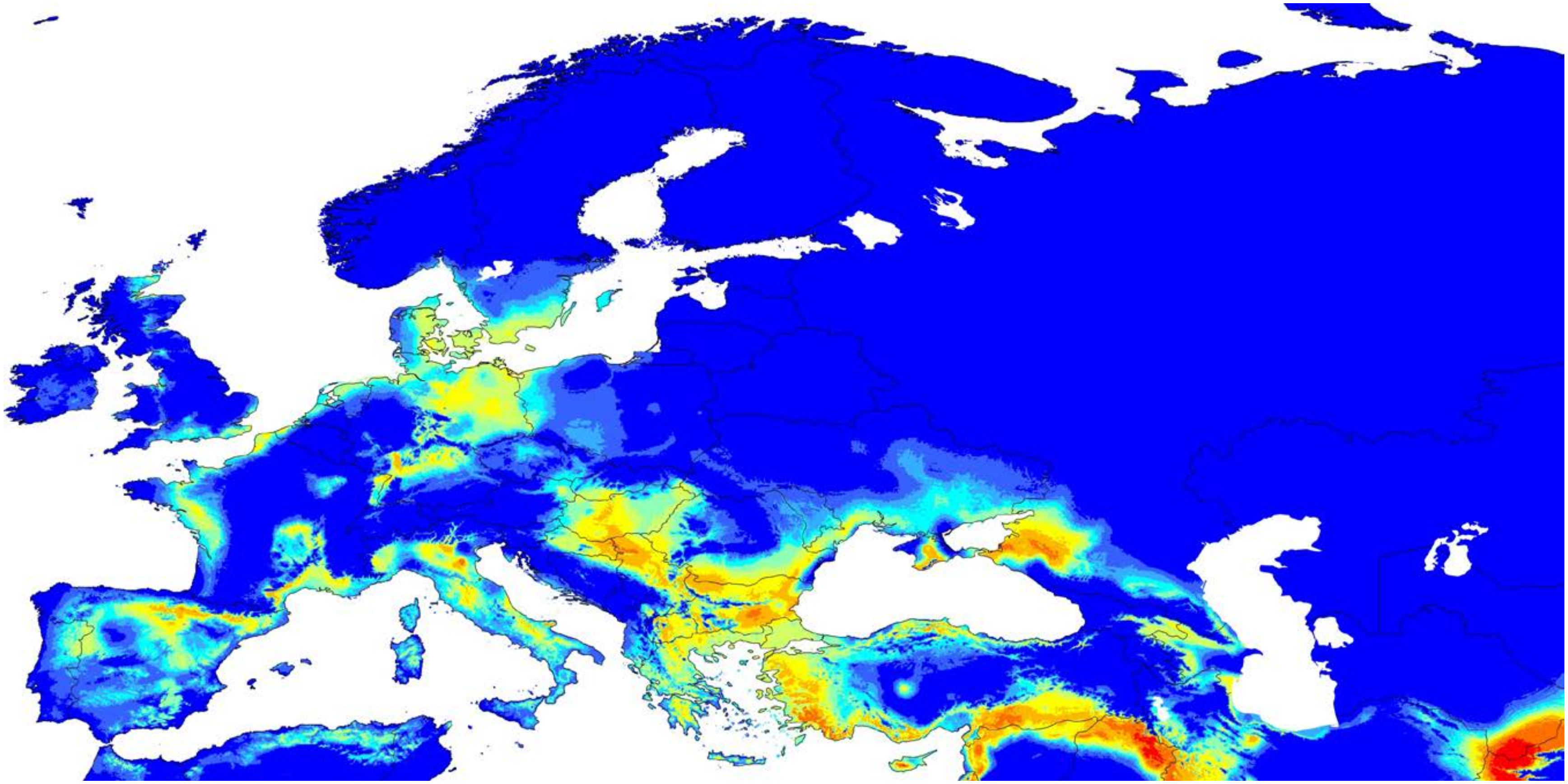

CURRENT *T. karelinii* central species

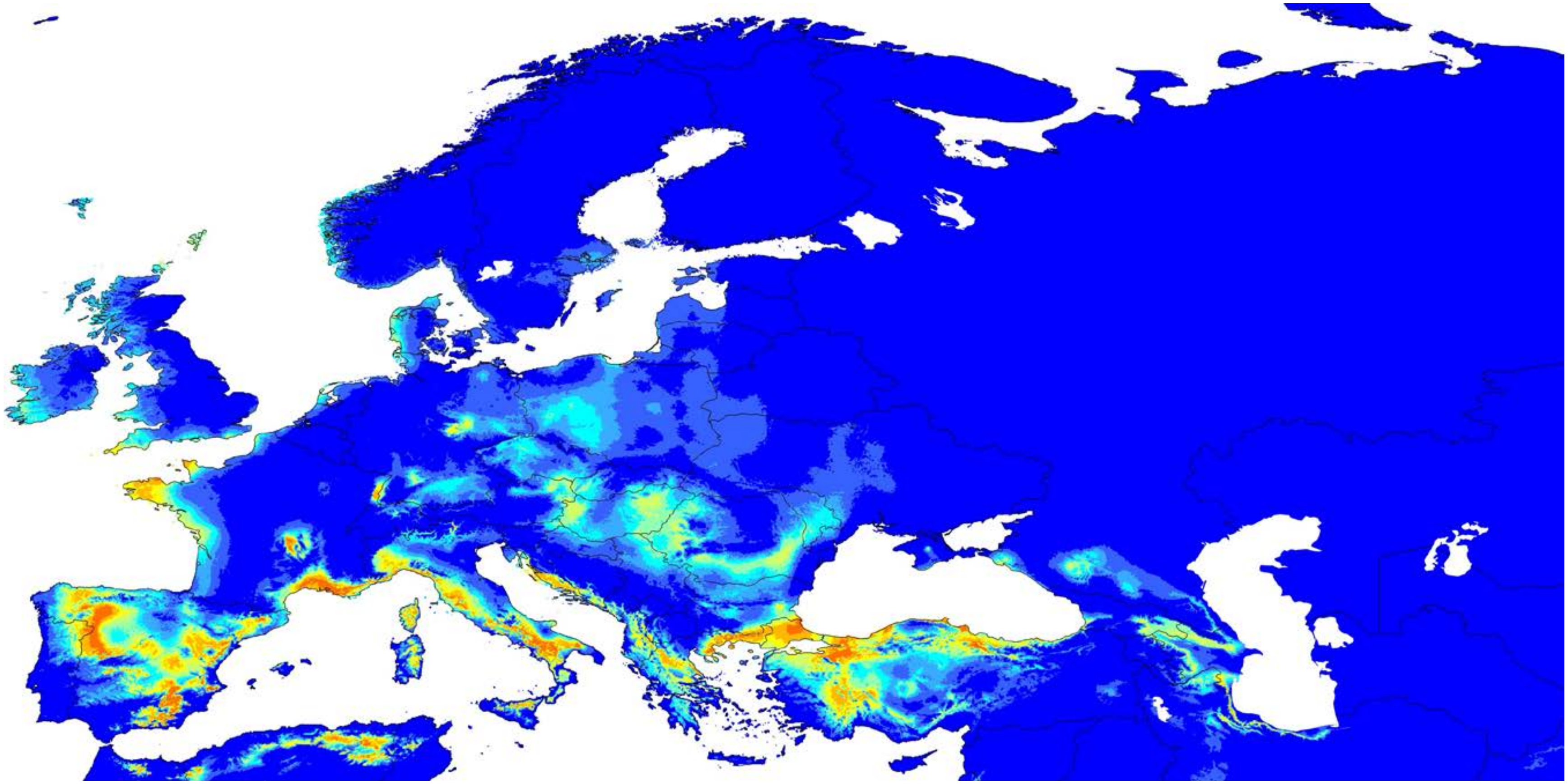

CURRENT *T. karelinii* eastern species

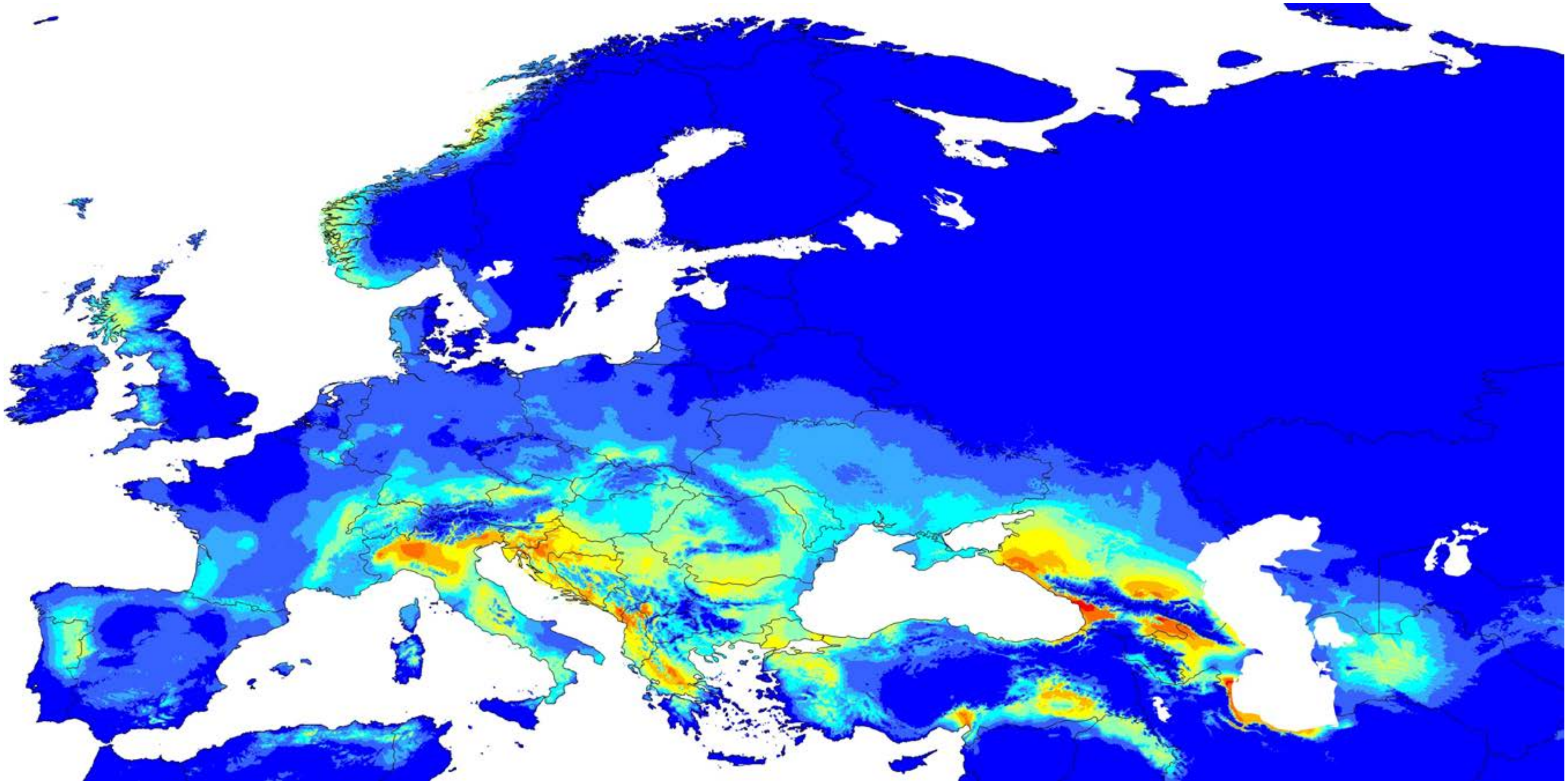

CURRENT *T. macedonicus*

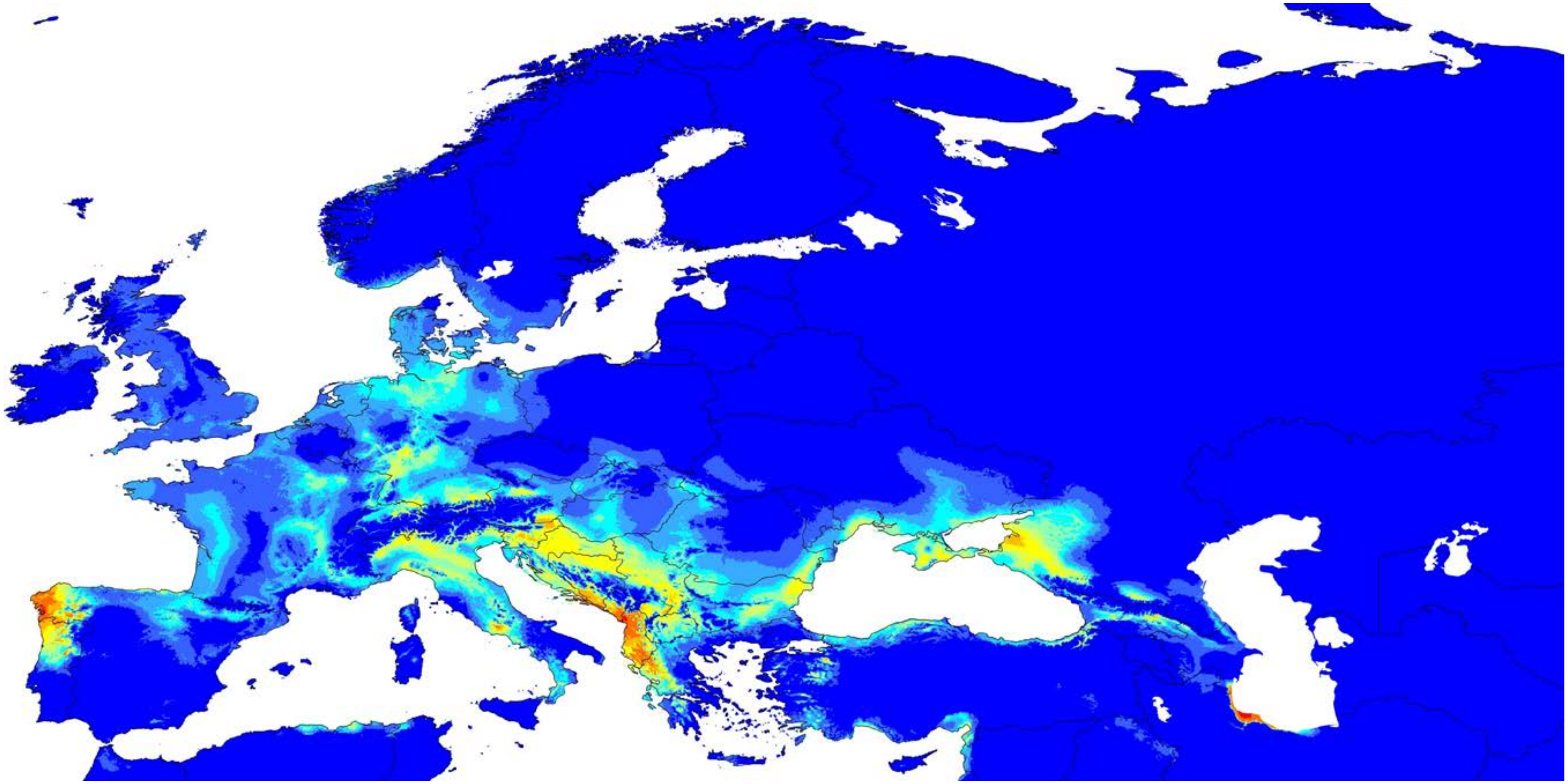

CURRENT *T. marmoratus*

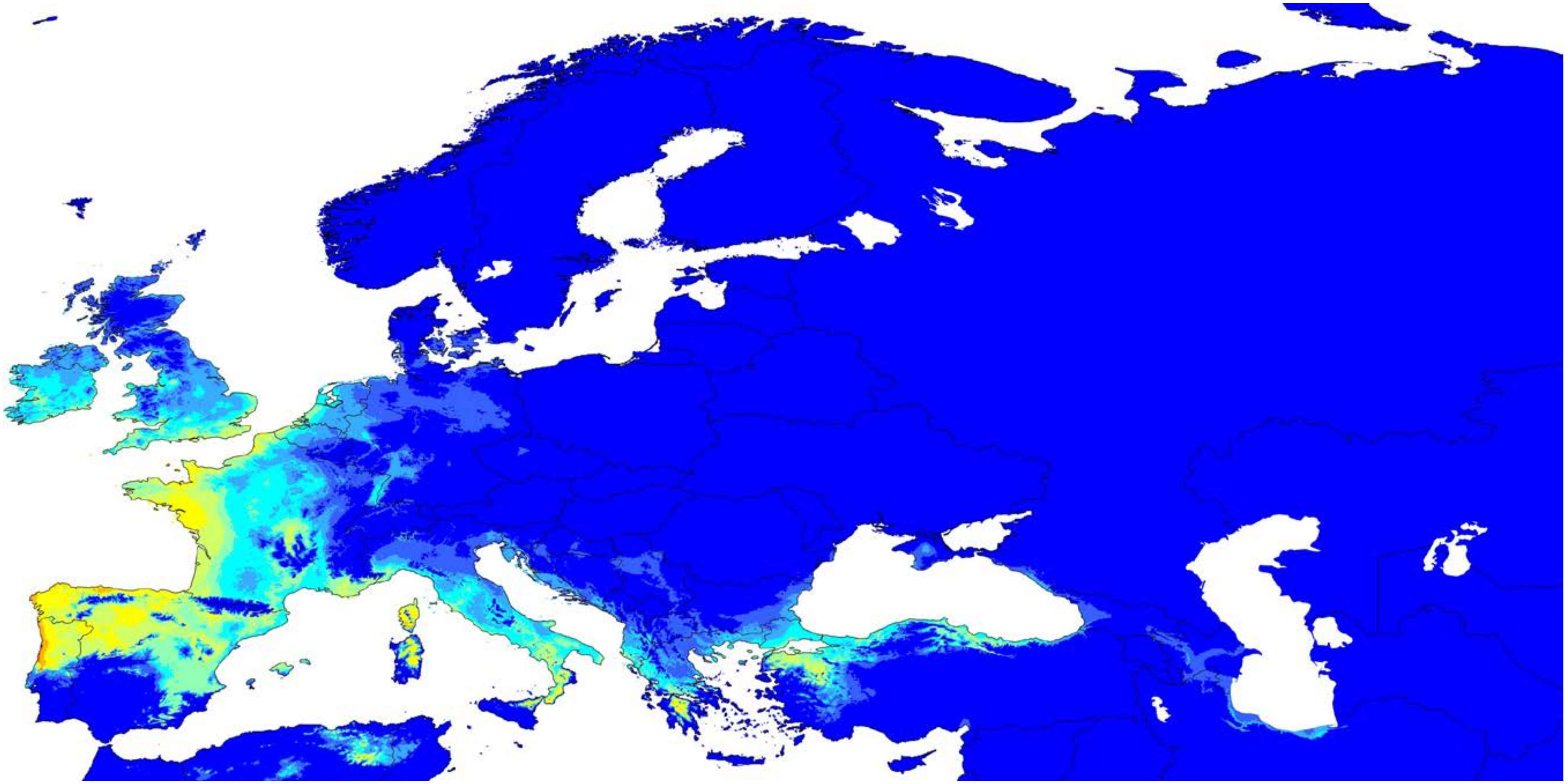

CURRENT *T. pygmaeus*

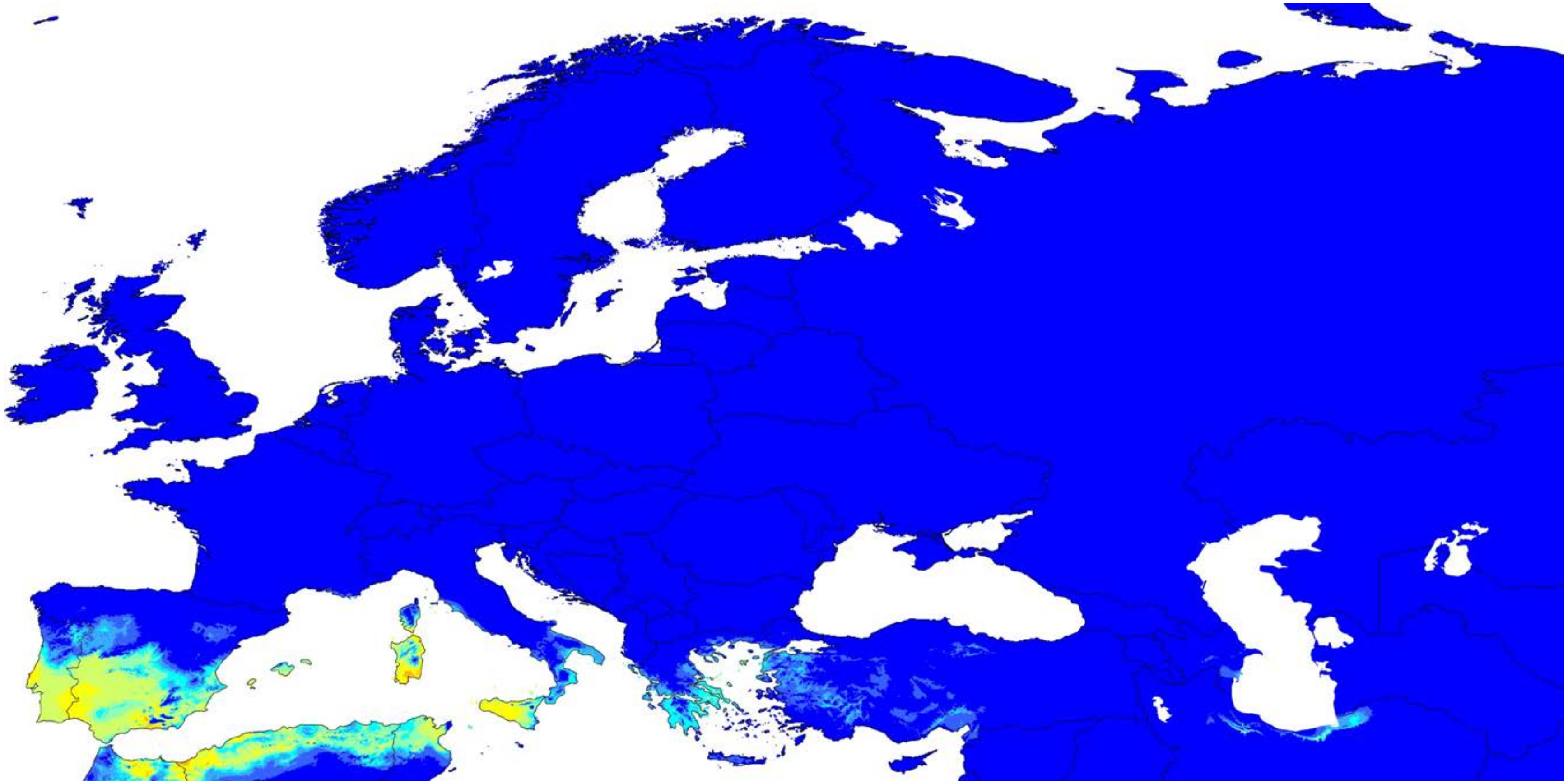

Supplement: Additional file 7 — Full species distribution models. The species distribution model of each Triturus species projected for Last Glacial Maximum and current climate conditions, not cut according to the current species ranges. [file 1742-9994-10-13-S7.pdf]
